# Protein Amphipathic Helix Insertion: A Mechanism to Induce Membrane Fission

**DOI:** 10.3389/fcell.2019.00291

**Published:** 2019-12-10

**Authors:** Mikhail A. Zhukovsky, Angela Filograna, Alberto Luini, Daniela Corda, Carmen Valente

**Affiliations:** Institute of Biochemistry and Cell Biology, National Research Council, Naples, Italy

**Keywords:** membrane fission, membrane scission, fission-inducing protein, amphipathic helix, shallow insertion, lipid cofactor, lipid-binding site, neck-hemifission model

## Abstract

One of the fundamental features of biomembranes is the ability to fuse or to separate. These processes called respectively membrane fusion and fission are central in the homeostasis of events such as those related to intracellular membrane traffic. Proteins that contain amphipathic helices (AHs) were suggested to mediate membrane fission via shallow insertion of these helices into the lipid bilayer. Here we analyze the AH-containing proteins that have been identified as essential for membrane fission and categorize them in few subfamilies, including small GTPases, Atg proteins, and proteins containing either the ENTH/ANTH- or the BAR-domain. AH-containing fission-inducing proteins may require cofactors such as additional proteins (e.g., lipid-modifying enzymes), or lipids (e.g., phosphatidylinositol 4,5-bisphosphate [PtdIns(4,5)P_2_], phosphatidic acid [PA], or cardiolipin). Both PA and cardiolipin possess a cone shape and a negative charge (−2) that favor the recruitment of the AHs of fission-inducing proteins. Instead, PtdIns(4,5)P_2_ is characterized by an high negative charge able to recruit basic residues of the AHs of fission-inducing proteins. Here we propose that the AHs of fission-inducing proteins contain sequence motifs that bind lipid cofactors; accordingly (K/R/H)(K/R/H)xx(K/R/H) is a PtdIns(4,5)P_2_-binding motif, (K/R)x_6_(F/Y) is a cardiolipin-binding motif, whereas KxK is a PA-binding motif. Following our analysis, we show that the AHs of many fission-inducing proteins possess five properties: (a) at least three basic residues on the hydrophilic side, (b) ability to oligomerize, (c) optimal (shallow) depth of insertion into the membrane, (d) positive cooperativity in membrane curvature generation, and (e) specific interaction with one of the lipids mentioned above. These lipid cofactors favor correct conformation, oligomeric state and optimal insertion depth. The most abundant lipid in a given organelle possessing high negative charge (more negative than −1) is usually the lipid cofactor in the fission event. Interestingly, naturally occurring mutations have been reported in AH-containing fission-inducing proteins and related to diseases such as centronuclear myopathy (amphiphysin 2), Charcot-Marie-Tooth disease (GDAP1), Parkinson’s disease (α-synuclein). These findings add to the interest of the membrane fission process whose complete understanding will be instrumental for the elucidation of the pathogenesis of diseases involving mutations in the protein AHs.

## Introduction

Membrane fission (or scission), the process opposite to membrane fusion, consists in the splitting of one membrane into two separate membranes; instead, membrane fusion is a process by which two biological membranes converge into one membrane (Falanga et al., 2009; Kozlov et al., 2010; Scott and Youle, 2010; Knorr et al., 2017). Several examples of membrane fission processes can be found in the literature: cell division (Bohuszewicz et al., 2016; Caspi and Dekker, 2018; Stoten and Carlton, 2018), endocytosis (Renard et al., 2015; Ferreira and Boucrot, 2018; Kaksonen and Roux, 2018; Mettlen et al., 2018; Sandvig et al., 2018; Genet et al., 2019), caveolae biogenesis (Parton et al., 2006; Kirkham et al., 2008; Ariotti et al., 2015), budding of vesicles from endomembranes (Lee et al., 2005; Krauss et al., 2008; Hagen et al., 2015; Park et al., 2019), nuclear envelope repair (Isermann and Lammerding, 2017), neuron pruning (Loncle et al., 2015), mitochondrial division (Adachi et al., 2016; Huber et al., 2016; Pagliuso et al., 2018; Tandler et al., 2018; Tilokani et al., 2018; Agrawal and Ramachandran, 2019; Irajizad et al., 2019; Yoshida and Mogi, 2019), plastid division (Osteryoung and Pyke, 2014; Yoshida, 2018; Yoshida and Mogi, 2019), peroxisome fission (Huber et al., 2016; Schrader et al., 2016; Su et al., 2018), endosome fission (Daly and Cullen, 2018; Hoyer et al., 2018; Kitamata et al., 2019), vacuole fission (Gopaldass et al., 2017), Golgi membrane fission (Weigert et al., 1999; Hidalgo Carcedo et al., 2004; Bonazzi et al., 2005; Yang et al., 2005, 2008, 2011; Corda et al., 2006; Colanzi et al., 2007; Valente et al., 2013), macropinocytosis (Liberali et al., 2008), macroautophagy (Knorr et al., 2015; Yu and Melia, 2017; Osawa et al., 2019), microautophagy (Uttenweiler and Mayer, 2008; Li et al., 2012), thylakoid membrane remodeling (Chuartzman et al., 2008), formation of multivesicular bodies (Piper and Katzmann, 2007), sporulation in bacteria (Doan et al., 2013; Gifford and Meyer, 2015); formation of bacterial membrane vesicles (Eriksson et al., 2009; Schwechheimer and Kuehn, 2015; Bitto and Kaparakis-Liaskos, 2017), bacterial chromatophores (Bohuszewicz et al., 2016) and bacterial magnetosomes (Uebe and Schüler, 2016); formation of membrane vesicles in archaea (Ellen et al., 2010), virus budding from the cell (Rossman and Lamb, 2013; Adu-Gyamfi et al., 2015; Bigalke and Heldwein, 2015; Herneisen et al., 2017). Most of these membrane fission reactions are mediated by specialized proteins (Campelo and Malhotra, 2012; Frolov et al., 2015; Renard et al., 2015) that include the AH-containing proteins (see, e.g., Antonny et al., 1997; Farsad et al., 2001; Boucrot et al., 2012; Martyna et al., 2017).

Based on the many examples of membrane fission driven by AH insertion into the membrane bilayer, we have summarized in this review the known AH-containing proteins and related mechanisms linked to the fission event. Following a brief outline on the hemifission intermediate, we here propose to divide the membrane fission processes in four classes based on direct/indirect energy consumption and on the budding toward the cytosol or away from the cytosol. We then discuss the fission events mediated by the AH-containing proteins and underline the role of specific lipid cofactors and of the oligomeric state. We also report the naturally occurring mutations of these proteins.

## Protein-Mediated Membrane Fission

### Stages of Fission Process: “Neck-Hemifission” Model

Membrane fission proceeds through the following steps: membrane neck intermediate, hemifission intermediate, and two separate membrane formation (Corda et al., 2002; Kozlovsky and Kozlov, 2003; Bashkirov et al., 2008; Campelo and Malhotra, 2012; Frolov et al., 2015; Antonny et al., 2016; McDargh and Deserno, 2018; Pannuzzo et al., 2018). Hemifission is an intermediate state where the proximal monolayer (also called contacting monolayer) of the bilayer forming the neck coalesces, thus breaking the inner volume into two parts, while the distal monolayer remains continuous. Separation of distal monolayers completes the fission process. The existence of hemifission intermediate is confirmed by experimental observation that membrane fission is non-leaky (Bashkirov et al., 2008).

Neck and hemifission intermediates of the membrane fission process resemble pore and hemifusion intermediates of the opposite membrane fusion process. Membrane fusion proceeds via an intermediate named stalk, a lipidic hourglass-shaped connection between the contacting membrane leaflets. As a result of the radial expansion of the stalk, a hemifusion diaphragm forms. This intermediate is a bilayer formed by two distal leaflets of the fusing membranes. Finally, a fusion pore forms and establishes continuity between aqueous spaces of fusing membrane-enclosed compartments. The difference between membrane neck (sometimes referred to as “fission pore”) and fusion pore is that neck narrows, while fusion pore expands. By analogy with the “stalk-pore model” of membrane fusion (Chernomordik and Kozlov, 2008; Martens and McMahon, 2008; Kielian, 2014; Podbilewicz, 2014; Zhukovsky et al., 2019a; and references therein), we will call this membrane fission pathway as “neck-hemifission model.”

### Classes of Membrane Fission Reactions

Renard et al. (2018) suggested to classify membrane fission mechanisms into two main categories: active fission (with the direct consumption of cellular energy by nucleoside triphosphate hydrolysis) and passive fission (without the direct use of energy). Many membrane fission processes are dependent on the interaction of fission-inducing proteins with specific lipid molecules (see below). In some cases, passive fission might be energized indirectly, via the energy used in the synthesis of these lipid cofactors (Gopaldass et al., 2017).

**FIGURE 1:**
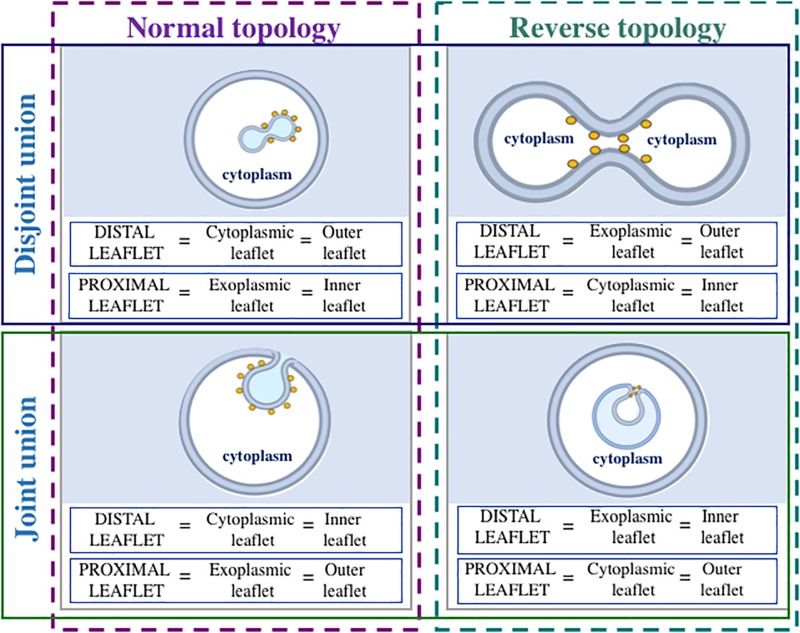
Nomenclature of leaflets (monolayers) during different kinds of membrane fission reaction. Only membranes decorated with fission-inducing proteins (yellow circles) are considered. When membrane fission takes place, separation of proximal leaflets is followed by separation of distal leaflets. During normal topology fission (membrane-enclosed compartment buds toward the cytoplasm), distal leaflet is cytoplasmic, whereas proximal leaflet is exoplasmic. During reverse topology fission (membrane-enclosed compartment buds away from the cytoplasm), distal leaflet is exoplasmic, and proximal leaflet is cytoplasmic. During both normal and reverse topology fission reactions, disjoint or joint union of membrane-enclosed compartments can form. If disjoint union forms, outer leaflet is distal, and inner leaflet is proximal. When joint union forms, outer leaflet is proximal, whereas inner leaflet is distal. Fission-inducing proteins are usually present on the cytoplasmic side of the membrane.

**FIGURE 2:**
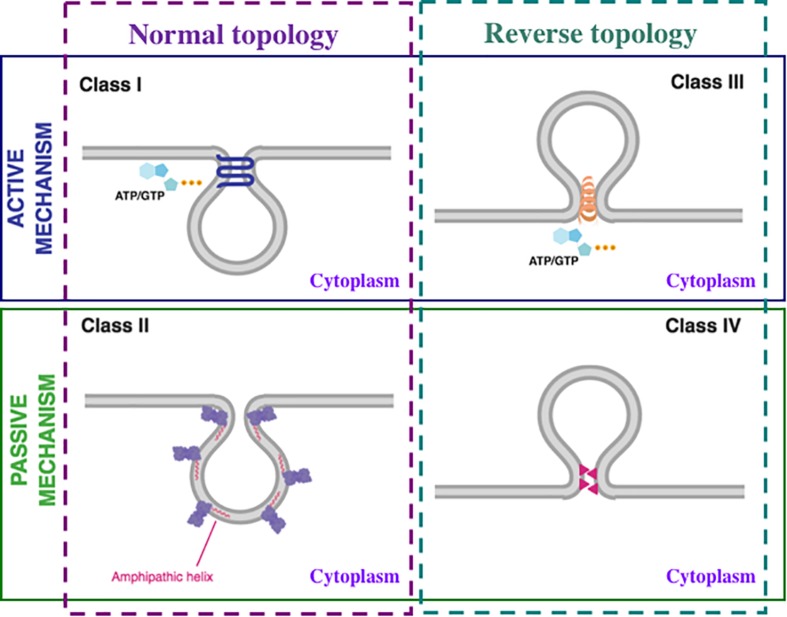
Mechanisms of membrane fission. Membrane fission mechanisms can be classified as active, that require consumption of cellular energy by nucleoside triphosphate (ATP or GTP) hydrolysis (upper panel; blue line) or passive that do not require the direct use of energy (lower panel; green line). Each of these above-mentioned categories can be further divided into two types: normal topology, when the vesicle buds toward the cytoplasm (left panel; purple dashed line) and reverse topology, when the vesicle buds away from the cytoplasm (right panel; dark green dashed line). Consequently, membrane fission mechanisms are divided into four classes: Class I, active mechanism with normal topology; Class II, passive mechanism with normal topology; Class III, active mechanism with reverse topology; Class IV, passive mechanism with reverse topology. The details are in the text.

Moreover, membrane fission processes can be classified into two types: “normal topology fission” (membrane-enclosed compartments bud toward the cytosol) and “reverse topology fission” (membrane-enclosed compartments bud away from the cytosol) (Votteler and Sundquist, 2013; Frolov et al., 2015; Schöneberg et al., 2017; Caspi and Dekker, 2018; Snead and Stachowiak, 2018). During normal topology fission, distal monolayer of a membrane-enclosed compartment is cytoplasmic, whereas proximal monolayer is exoplasmic (Figure 1). During reverse topology fission, distal monolayer is exoplasmic, and proximal monolayer is cytoplasmic (Figure 1). We would like to point out that after “normal topology fission,” disjoint union of two membrane-enclosed compartments (as in the case of formation of post-Golgi carriers) or joint union (as in the case of endocytosis) can form. Similarly, after “reverse topology fission,” disjoint union (as in the case of cell division) or joint union [as in the case of herpesvirus primary envelopment (Bailer, 2017; Klupp et al., 2018) or formation of multivesicular bodies (Piper and Katzmann, 2007)] can form (Figure 1). Based on these considerations, we propose to divide membrane fission processes into four classes (Figure 2):

Class I: Active mechanism, normal topology. Examples: fission mediated by the large GTPase dynamin and other members of the dynamin superfamily (Daumke and Praefcke, 2016; Ramachandran and Schmid, 2018; Ford and Chappie, 2019; Jimah and Hinshaw, 2019) and by the small AH-containing GTPases Arf1 (Krauss et al., 2008; Beck et al., 2011; Bottanelli et al., 2017; Dodonova et al., 2017) and Sar1 (Bielli et al., 2005; Lee et al., 2005; Bacia et al., 2011; Hariri et al., 2014; Hanna et al., 2016; Melero et al., 2018).

Class II: Passive mechanism, normal topology. Examples: fission mediated, in the absence of nucleoside triphosphate hydrolysis, by *Bacillus subtilis* FisB (Doan et al., 2013), C-terminal Binding Protein 1 Short form/Brefeldin A ADP-Ribosylation substrate (CtBP1-S/BARS) (Spanò et al., 1999; Weigert et al., 1999; Hidalgo Carcedo et al., 2004; Valente et al., 2005, 2012; Liberali et al., 2008; Pagliuso et al., 2016; Zhukovsky et al., 2019b), and by numerous AH-containing proteins, such as endophilins (Rostovtseva et al., 2009; Ambroso et al., 2014; Genet et al., 2019), amphiphysins (Wu and Baumgart, 2014; Snead et al., 2019), epsins (Ford et al., 2002; Boucrot et al., 2012; Brooks et al., 2015), α-synuclein (Nakamura et al., 2011; Braun et al., 2017; Pozo Devoto and Falzone, 2017; Fakhree et al., 2019), GDAP1 (Huber et al., 2016), ankyrin repeats and KH domain-containing protein 1 (ANKHD1) (Kitamata et al., 2019), *Saccharomyces cerevisiae* Atg18 (Gopaldass et al., 2017), *Agrobacterium tumefaciens* PmtA (Danne et al., 2017b), EcMurG (van den Brink-van der Laan et al., 2003), *Acholeplasma laidlawii* MGS (Eriksson et al., 2009; Ariöz et al., 2014; Ge et al., 2014) and DGS (Eriksson et al., 2009).

Class III: Active mechanism, reverse topology. Examples: fission mediated by the ESCRT machinery (Chiaruttini and Roux, 2017; Schöneberg et al., 2017; Stoten and Carlton, 2018; Ahmed et al., 2019; Gatta and Carlton, 2019), bacterial cell division (Bohuszewicz et al., 2016; Vedyaykin et al., 2019) and archaeal cell division (Caspi and Dekker, 2018). Of note, Vps4 ATPase (Monroe et al., 2014; Caspi and Dekker, 2018), BDLP1 containing N-terminal GTPase domain (Bohuszewicz et al., 2016), and CdvC containing ATPase subunit (Caspi and Dekker, 2018) proteins provide energy for the ESCRT pathway, bacterial cell division, and archaeal cell division, respectively. Interestingly, archaeal CdvC is the homolog of eukaryotic Vps4 (Caspi and Dekker, 2018).

Class IV: Passive mechanism, reverse topology. Examples: fission mediated by the herpesvirus fission machine (Bigalke and Heldwein, 2015), influenza A virus AH-containing protein M2 (Herneisen et al., 2017; Martyna et al., 2017; Madsen et al., 2018), Ebola virus VP40 (Soni and Stahelin, 2014; Adu-Gyamfi et al., 2015).

Although in Renard et al. (2018) CtBP1-S/BARS-mediated fission is classified as an active fission mechanism, we propose that this process should be considered as passive, because no direct consumption of energy by nucleoside triphosphate hydrolysis takes place during this process.

Any membrane fission reaction belongs to the category of active or passive fission and almost all fission processes can be classified as normal topology or reverse topology fission. Moreover, only very few fission processes (such as division of IMMs) are mediated by proteins not residing in the cytosol. Therefore, with very few exceptions, all fission reactions belong to one of the four classes described above. Moreover, many fission reactions mediated by AH-containing proteins are very diverse. However, assigning of these reactions to one of four classes I–IV will allow to find similarities in fission processes which seem to be completely different.

## Fission Driven by Amphipathic Helix-Containing Proteins

Amphipathicity is the segregation of hydrophobic and hydrophilic amino acid residues between the two opposite faces of the protein α-helix, a distribution well suited for membrane binding (Drin and Antonny, 2010; Giménez-Andrés et al., 2018). A hydrophobic moment was introduced (Eisenberg et al., 1982, 1984) to estimate whether a protein sequence, when considered as helical, exhibits one polar and one hydrophobic face (Drin and Antonny, 2010). A large value of hydrophobic moment suggests that the protein sequence can be folded as AH perpendicularly to its axis (Eisenberg et al., 1982).

Various fission reactions are known to be driven by the large GTPase dynamin and other mechanoenzymes belonging to the dynamin superfamily (Daumke and Praefcke, 2016; Ramachandran and Schmid, 2018; Ford and Chappie, 2019; Jimah and Hinshaw, 2019; and references therein). In other fission processes that are not dependent on mechanoenzymes, proteins that contain AHs often play an important role (see, e.g., Antonny et al., 1997; Farsad et al., 2001; Boucrot et al., 2012; Martyna et al., 2017; and references therein).

Amphipathic helices are involved in many biological processes such as protein–protein interactions or interaction with biomembranes (Segrest et al., 1990, 1992). When an AH binds to membrane, it orients itself parallel to the membrane plane. The hydrophobic side of this helix inserts into the interior of the bilayer, while residues of the hydrophilic side interact with the lipid headgroups (Drin and Antonny, 2010; Roberts et al., 2013). The insertion of AHs into the bilayer promotes membrane curvature thus supporting the fission reaction (Campelo et al., 2008; Martyna et al., 2017).

Moreover, some protein segments (named “AH motifs”) of membrane-binding proteins are not completely helical in solution, but become AHs upon interaction with the membrane (Cornell and Taneva, 2006; Drin and Antonny, 2010; Ambroso et al., 2014; Chong et al., 2014; Horchani et al., 2014; Barneda et al., 2015; Frolov et al., 2015; and references therein). The presence of anionic lipids in the membrane often promotes the AH folding (see, for example, Davidson et al., 1998; Kweon et al., 2006; Fernandes et al., 2008; Horchani et al., 2014; Brady et al., 2015; Mizuno et al., 2017; Ryan et al., 2018).

The ability to sense membrane curvature by some proteins favors their interaction with curved membranes. This process not always leads to curvature generation. However, other AHs are able to induce curvature (Drin and Antonny, 2010; Wilz et al., 2011), an event required for proteins mediating membrane fission. The basic principles governing membrane curvature generation remain to be clarified (Khattree et al., 2013).

Amphipathic helices characterized by the presence of basic residues at the polar-non-polar interface are well suited for interaction with membranes (Mishra and Palgunachari, 1996; Davidson et al., 1998; Polozov et al., 1998; Mozsolits et al., 2004), especially with those containing negatively charged phospholipids by electrostatic attraction (Polozov et al., 1998). It has been hypothesized that interfacial positively charged residues help to anchor the AH in the lipid bilayer (Mishra and Palgunachari, 1996). We would like to emphasize that the AHs of many fission-inducing proteins contain at least two basic residues (Arg and/or Lys) each, at the polar-non-polar interface. Examples are: H0 helix of endophilin A1 contains Lys7 and Lys16 (Ambroso et al., 2014); AH of PmtA contains Lys6, Arg8, Lys12 (Danne et al., 2017b); AH of Atg18 contains Arg372 and Arg377 (Gopaldass et al., 2017). In PmtA, Arg8 and Lys12 are involved in protein attachment to anionic lipids (Danne et al., 2017b). Overall, the AHs of many fission proteins contain at least three positively charged residues. On the contrary, the AHs of proteins that sense but do not generate membrane curvature, such as ArfGAP1, GMAP-210, Kes1p, often contain not more than one basic residue (Drin and Antonny, 2010).

Campelo et al. (2008), similarly to Zemel et al. (2008), predicted that shallow (penetrating ca. 40% of the monolayer thickness) insertion of AH is particularly effective in the generation of membrane curvature. Since AHs and AH-containing protein segments are particularly suitable as membrane curvature-generating inclusions, their shallow insertions were suggested to be sufficient to mediate membrane fission (Boucrot et al., 2012).

The insertion depth of the AHs of fission-inducing proteins was studied experimentally. Using two hydrophobic quenchers of tryptophan fluorescence (shallow quencher and deep quencher), Hanna et al. (2016) detected shallow penetration of the Sar1B AH into the membrane, just below the hydrophilic headgroups. EPR measurements demonstrated shallow insertion of the AHs of endophilin A1 (Gallop et al., 2006), epsin (Lai et al., 2012), and α-synuclein (Jao et al., 2008) that embed at the level of lipid phosphate groups (Gallop et al., 2006; Lai et al., 2012) or just below this level (Jao et al., 2008). According to molecular dynamics simulations, the insertion depth of AHs from epsin and Sar1p is lower than that of Arf1, and these helices from epsin and Sar1p generate higher membrane curvature than the AH from Arf1 (Li, 2018). Interaction of the AHs of fission-inducing proteins with specific lipids might ensure the optimal insertion depth for the generation of membrane curvature and for the interaction with other fission-inducing proteins, as discussed below.

An additional mechanism foresees a cooperativity among proteins where the first insertion of an AH from a given protein able to sense membrane curvature (i.e., to bind selectively to curved membranes) favors the insertion of additional AHs (Gallop et al., 2006; Lundmark et al., 2008; Miller et al., 2015; Hanna et al., 2016; Martyna et al., 2017). In turn, these AHs induce more membrane curvature facilitating the insertion of additional AHs. Due to this positive cooperativity in membrane curvature generation, the runaway process leading to membrane fission might take place once a critical concentration of AH-containing fission-inducing proteins is reached (Miller et al., 2015).

Amphipathic helix-containing fission-inducing proteins are recognized as a separate superfamily of scission factors (Gallop et al., 2006; Boucrot et al., 2012; Martyna et al., 2017). Among these proteins there are: Arf1 (Krauss et al., 2008; Beck et al., 2011; Bottanelli et al., 2017; Dodonova et al., 2017), Sar1 (Bielli et al., 2005; Lee et al., 2005; Bacia et al., 2011; Hariri et al., 2014; Hanna et al., 2016; Melero et al., 2018), epsins (Ford et al., 2002; Boucrot et al., 2012; Brooks et al., 2015), CALM (Miller et al., 2015), endophilin A1 (Gallop et al., 2006; Ambroso et al., 2014), endophilin A2 (Boucrot et al., 2015; Renard et al., 2015; Simunovic et al., 2017; Genet et al., 2019), endophilin A3 (Boucrot et al., 2012), endophilin B1 (Rostovtseva et al., 2009; Takahashi et al., 2016), mammalian amphiphysin 1 (Snead et al., 2019) and amphiphysin 2 (Wu and Baumgart, 2014), *Drosophila* amphiphysin (Isas et al., 2015), PICK1 (Karlsen et al., 2015), α-synuclein (Nakamura et al., 2011; Braun et al., 2017; Pozo Devoto and Falzone, 2017; Fakhree et al., 2019), GDAP1 (Huber et al., 2016), caveolin-1 (Parton et al., 2006; Kirkham et al., 2008; Ariotti et al., 2015), ANKHD1 (Kitamata et al., 2019), peroxins Pex11B (Yoshida et al., 2015) and Pex11p (Opaliński et al., 2011; Su et al., 2018), yeast PROPPIN Atg18 (Gopaldass et al., 2017), Snf7 belonging to the ESCRT complex (Buchkovich et al., 2013), influenza A virus M2 (Rossman et al., 2010; Roberts et al., 2013; Herneisen et al., 2017; Madsen et al., 2018), and bacterial enzymes PmtA (Danne et al., 2017b), MurG (van den Brink-van der Laan et al., 2003; Lind et al., 2007; Albesa-Jové et al., 2014), MGS (Lind et al., 2007; Eriksson et al., 2009; Ge et al., 2014), and DGS (Eriksson et al., 2009). Unlike wt proteins, mutants of Arf1 (Beck et al., 2011), Sar1 (Lee et al., 2005), endophilin A1 (Farsad et al., 2001; Masuda et al., 2006; Mim et al., 2012), *Drosophila* amphiphysin (Yoon et al., 2012), mammalian amphiphysin 1 (Farsad et al., 2001), Atg18 (Gopaldass et al., 2017) lacking AHs were unable to generate membrane curvature.

Increasing of the hydrophobic moment favors folding of AH motif into an α-helix and binding to membrane (Drin and Antonny, 2010). Thus, it is not surprizing that mutations of hydrophobic residues, within AHs, to Ala or to hydrophilic residues (substitutions that decrease hydrophobic moment) inhibit the ability of fission proteins such as Arf1 (Krauss et al., 2008), Sar1p (Lee et al., 2005), endophilin A1 (Farsad et al., 2001; Gallop et al., 2006; Masuda et al., 2006; Suresh and Edwardson, 2010), epsin (Ford et al., 2002; Boucrot et al., 2012), Pex11p (Opaliński et al., 2011), Pex11B (Yoshida et al., 2015), yeast Atg2 (Kotani et al., 2018), mammalian Atg2A (Tamura et al., 2017), caveolin-1 (Parton et al., 2006; Kirkham et al., 2008; Ariotti et al., 2015), M2 (Rossman et al., 2010; Roberts et al., 2013), PmtA (Danne et al., 2017b), to generate membrane curvature. On the contrary, mutations of hydrophobic residues to more bulky hydrophobic Trp (substitutions that increase hydrophobic moment) promoted the ability of Arf1 (Krauss et al., 2008), epsin (Ford et al., 2002; Boucrot et al., 2012), and Pex11p (Opaliński et al., 2011) to induce membrane curvature, see Table 1.

**TABLE 1:** Mutations in amphipathic helices of fission-inducing proteins that inhibit or promote the ability of these proteins to generate membrane curvature.

**Protein**	**Amino acid mutations and effects**	**Amphipathic helix**
Arf1	I4Q, I4Q/F5E: reduced tubulation (Krauss et al., 2008). F5W, numerous membrane tubules at a frequency approx. three times higher than wt (Krauss et al., 2008)	G2-R19 (Krauss et al., 2008)
Sar1 (mammalian)	Y9F/G11P/S14F: deficiency in vesicle release (Bielli et al., 2005)	M1-F18 (Bielli et al., 2005)
Sar1p (yeast)	W4A, I6A/F7A, W9A, F10A: deficient in tubulation (Lee et al., 2005)	M1-G18 (Lee et al., 2005)
Endophilin A1	F10E: reduced tubulation (Farsad et al., 2001; Gallop et al., 2006). K76E/R78E: tubulation decreased (Gallop et al., 2006). A66D: lost ability to form tubes (Masuda et al., 2006). A63S/A66S, A63S/A66S/M70Q: reduced number of tubes (Masuda et al., 2006). A66W: switched the bilayer deformation to vesiculation (Masuda et al., 2006; Suresh and Edwardson, 2010)	H0 helix: S2-K16; insert helix: A63-G79 (Ambroso et al., 2014)
Epsin	L6E: less efficient vesiculation (Boucrot et al., 2012), L6Q: failed to tubulate, L6H: less markedly tubulation (Ford et al., 2002). L6W: increase in vesiculation (Ford et al., 2002; Boucrot et al., 2012)	S4-V14 (Lai et al., 2012)
Caveolin-1	S80E, F81E, W85E: reduction in the potential to perform fission (Ariotti et al., 2015)	S80-T95 (Parton et al., 2006; Kirkham et al., 2008)
Atg2A (mammalian)	P1750E/V1754E/F1758E/F1761E/L1765E: defects in autophagy (Tamura et al., 2017)	P1750-L1767 (Tamura et al., 2017)
Atg2 (yeast)	F1352D/I1355D: defects in autophagy (Kotani et al., 2018)	A1347-E1373 (Kotani et al., 2018)
Pex11B (mammalian)	L58E/L61E/A65E, L59P/D66P: lack fission activity (Yoshida et al., 2015)	S53-S70 (Yoshida et al., 2015)
Pex11p (fungal)	I69W/A83W: increase in tubulation (Opaliński et al., 2011). I69E/I72E/F75E, M70P/E77P: tubulation was completely impaired (Opaliński et al., 2011). F75R/H78R/L79R: lower ability to deform liposomes (Su et al., 2018)	T66-A83 (Opaliński et al., 2011)
AtPmtA (bacterial)	F13A/V24A: abolished membrane-remodeling activity (Danne et al., 2017b)	K6-V24 (Danne et al., 2017b)
M2 from influenza A virus	F47A/F48A/I51A/Y52A/F55A (Rossman et al., 2010), in five residues (F47, F48, I51, Y52, F55) two were mutated to alanine at a time (Roberts et al., 2013): loss of scission	R45-G62 (Rossman et al., 2010)

Substitution of positively charged residues of the AH hydrophilic face for negatively charged residues leads to repulsion from similarly charged membrane and, thus, to the reduced ability of AH to induce membrane curvature. This conclusion was confirmed experimentally. Mutations of basic Lys and Arg to negatively charged Glu within AH of endophilin A1 (Gallop et al., 2006) inhibited the ability of this fission-inducing protein to generate membrane curvature.

The influence of the mutations of AH residues on the ability of AH-containing proteins to generate membrane curvature is summarized in Table 1.

Some of AH-containing fission-inducing proteins, such as the small GTPases Arf1 and Sar1, mediate class I fission reactions. Other proteins, such as yeast Atg18 and bacterial enzymes PmtA, MurG, MGS, DGS, drive class II fission processes. Snf7 protein is involved in class III membrane fission reactions of the ESCRT pathway. Influenza virus M2 mediates class IV fission reaction. However, some AH-containing proteins, such as endophilin B1 and amphiphysin 1, could be involved, in complex with mechanoenzymes belonging to the dynamin superfamily, in class I fission reactions (Takahashi et al., 2016; Takeda et al., 2018), or, in the absence of mechanoenzymes, in class II fission reactions (Rostovtseva et al., 2009; Snead et al., 2019). Hence, belonging to a certain class is a property of the process, not a property of the protein molecule.

We should keep in mind that some membrane fission-inducing protein complexes, such as herpesvirus fission machine (Bigalke and Heldwein, 2015; Lorenz et al., 2015; Klupp et al., 2018), Ebola virus fission protein VP40 (Soni and Stahelin, 2014; Adu-Gyamfi et al., 2015), and FisB that mediates fission during sporulation in *B. subtilis* (Doan et al., 2013), contain neither proteins belonging to the dynamin superfamily nor AH-containing proteins.

## Subfamilies of Amphipathic Helix-Containing Fission-Inducing Proteins

At least four subfamilies of proteins containing AHs able to drive membrane fission can be identified so far: small GTPases, ENTH/ANTH domain-containing proteins, BAR domain-containing proteins, and Atg proteins. Moreover, we suppose that synuclein family of proteins can be also considered as a subfamily of AH-containing fission-inducing proteins.

### Small GTPases

The small GTPases Sar1 (Bielli et al., 2005; Lee et al., 2005; Bacia et al., 2011; Hariri et al., 2014; Hanna et al., 2016; Melero et al., 2018) and Arf1 (Krauss et al., 2008; Beck et al., 2011; Bottanelli et al., 2017; Dodonova et al., 2017) are involved in the intracellular trafficking of proteins and lipids in COP-coated vesicles (Graham and Kozlov, 2010; Adolf et al., 2013; Cevher-Keskin, 2013; Yorimitsu et al., 2014; and references therein). Sar1 plays a role in the COPII-mediated anterograde trafficking from the ER to the Golgi apparatus, whereas Arf1 is involved in COPI-mediated retrograde trafficking from the Golgi to the ER, as well as in the intra-Golgi transport. Sar1 and Arf1, as well as other small GTPase family proteins, share structural similarities. These two proteins are highly conserved in evolution. Yeast *S. cerevisiae* has one Sar1, whereas mammals have two Sar1 paralogs, denoted Sar1A and Sar1B in humans (Loftus et al., 2012). Sar1 and Arf1 function as molecular switches. They are cytosolic and inactive when bound to GDP. Upon exchange of GDP to GTP, these small GTPases undergo a conformational change into their active form. In this way, the N-terminal AH becomes exposed. This helix has one hydrophobic face, which requires engagement in a hydrophobic environment and thus it inserts into the cytoplasmic-membrane leaflet (Graham and Kozlov, 2010; Adolf et al., 2013). The AH of Arf1 is myristoylated, unlike the AH of Sar1. Membrane-bound Sar1/Arf1 recruit coat proteins and initiate the formation of COP-coated vesicles. Insertion of the AHs of Sar1 and Arf1 into the membrane drives fission and, in turn, the release of these COP-coated vesicles (Bielli et al., 2005; Lee et al., 2005; Krauss et al., 2008; Hanna et al., 2016).

### ENTH/ANTH Domain-Containing Proteins

Epsins are highly conserved fission-inducing proteins that contain the N-terminal ENTH domain (Sen et al., 2012). Epsins play a role in the fission of CCVs during CME (Ford et al., 2002; Boucrot et al., 2012; Brooks et al., 2015). Four epsin paralogs are known in mammals: epsin 1 to 3 and epsin-related (EpsinR) (Takatori and Tomita, 2018). The role of epsin 1 in membrane curvature generation is well known (Ford et al., 2002; Kweon et al., 2006), although epsin 2 and epsin 3 are also involved in CME (Boucrot et al., 2012). The ENTH domain is approximately 140 residues long and has a compact globular structure of seven α-helices followed by an eighth helix that is not aligned with the other seven (Sen et al., 2012). Upon interaction with PtdIns(4,5)P_2_-containing membrane, ENTH domain of epsin 1 undergoes a conformational change that leads to the formation of an additional N-terminal α-helix denoted helix 0 (H0) (Ford et al., 2002) consisting of residues Met1-Tyr17 (Martyna et al., 2017), or residues Ser4–Val14, as reported by Lai et al. (2012). This is the helix inserting into the membrane. Primary sequences of the N-termini (residues 1–17) of human epsins 1, 2, 3 are very similar. It has been proposed that insertion of H0 helix into the bilayer induces membrane curvature required for fission (Ford et al., 2002; Boucrot et al., 2012), although recent study raised doubts on this mechanism and suggested that, instead, crowding of intrinsically disordered protein domains is able to drive fission (Snead et al., 2017).

Dynamin plays an important role in the fission of CCVs. Nevertheless, epsin is able to mediate CCV fission in dynamin-depleted cells. Boucrot et al. (2012) suggested that epsin molecules present at the neck of a budding vesicle might provide the force that destabilizes the neck of budding vesicle, thus leading to fission.

It should be emphasized that the role of epsins in the formation and fission of CCVs is somewhat similar to the role of Sar1 and Arf1 in the formation and fission of COP vesicles (Boucrot et al., 2012; Adolf et al., 2013). In both cases, an external trigger leads to the exposure of an AH that inserts into the cytosolic leaflet of the membrane and is assumed to drive the fission of vesicles. In case of small GTPases, exchange of GDP for GTP plays a role of such a trigger, whereas in case of epsin, AH exposure is triggered by the interaction with PtdIns(4,5)P_2_-containing membrane. Epsin, as well as small GTPases, might create a high-energy state at the neck of budding vesicle, and this state is relaxed by fission of this vesicle from the donor membrane (Boucrot et al., 2012; Adolf et al., 2013). Moreover, small GTPases are responsible for recruitment of COP proteins, whereas epsin is involved in clathrin coat assembly.

The CALM protein, also known as PICALM, is also involved in fission of CCVs during CME (Miller et al., 2015). CALM contains a N-terminal ANTH domain that is approximately twice as big as the ENTH domain, but has the same core of α-helices (Duncan and Payne, 2003; Legendre-Guillemin et al., 2004; Takatori and Tomita, 2018; Kaneda et al., 2019). ENTH and ANTH domains are so similar that several ANTH domains were originally designated ENTH domains, although later these two domains were subclassified as two distinct domains, ENTH and ANTH (Ford et al., 2002). Structurally, ENTH and ANTH domains are similar to the VHS domain (De Craene et al., 2012). Most ENTH and ANTH domains are lipid-binding domains, they interact specifically with phosphoinositides present in the membrane. However, the mechanisms of the interaction of ENTH and ANTH domains with PtdIns(4,5)P_2_ are quite different (Kaneda et al., 2019; and references therein).

ANTH domain-containing protein CALM, like ENTH domain-containing protein epsin 1, are characterized by an N-terminal AH that is supposed to insert into the membrane and to play a role in membrane fission (Miller et al., 2015). Proteins containing ENTH/ANTH domains (epsins 1, 2, 3 and CALM) are somewhat similar and can be classified as separate subfamily of AH-containing fission-inducing proteins. Possibly, the plant ANTH domain-containing protein AP180 also belongs to this subfamily (Kaneda et al., 2019). Similarly, new fission proteins belonging to this subfamily might be discovered in the future, based on the identification of AHs in their structure.

### BAR Domain-Containing Proteins

The BAR domain is found in many proteins implicated in various cellular processes, most of which are related to membrane remodeling (Stanishneva-Konovalova et al., 2016; Carman and Dominguez, 2018; Nishimura et al., 2018; Simunovic et al., 2019). BAR domains typically form banana-shaped homodimers, where each protomer consists of three-helix antiparallel coiled-coil structure. Because of its curved shape, the BAR domain is able to sense membrane curvature, i.e., to bind preferentially to curved membranes. Thus, the concave shape of the BAR-domain dimer is positively charged and interacts with the negatively charged cytoplasmic monolayer of biomembranes. Most BAR domain proteins also contain auxiliary domains involved in the interactions with other proteins or with membranes (Mim et al., 2012; Carman and Dominguez, 2018). Based on their structural properties, BAR domain proteins can be classified into three main groups: a classical BAR (including N-BAR), F-BAR, and I-BAR (Ahmed et al., 2010; Stanishneva-Konovalova et al., 2016; Nishimura et al., 2018). Proteins belonging to the N-BAR subgroup contain an N-terminal sequence H0 that folds into an AH upon membrane binding. Endophilins (Kjaerulff et al., 2011) and amphiphysins (Wu et al., 2009; Prokic et al., 2014) belong to the N-BAR subgroup and thus contain these H0-AH motifs. Moreover, endophilins contain an additional AH, known as H1I (helix 1 insert). In mammals endophilins are encoded by five genes: endophilins A1, A2, A3, B1, B2, whereas amphiphysins are encoded by two genes: amphiphysin 1 and amphiphysin 2 (also known as BIN1) (Kjaerulff et al., 2011; Prokic et al., 2014).

Bin/Amphiphysin/Rvs domain protein PICK1 contains an internal AH (Karlsen et al., 2015; Herlo et al., 2018). Mammalian endophilins A1 (Gallop et al., 2006; Ambroso et al., 2014), A2 (Boucrot et al., 2015; Renard et al., 2015; Simunovic et al., 2017; Genet et al., 2019), A3 (Boucrot et al., 2012) and B1 (Rostovtseva et al., 2009; Takahashi et al., 2016), mammalian amphiphysin 1 (Snead et al., 2019) and amphiphysin 2 (Wu and Baumgart, 2014), *Drosophila* amphiphysin (Isas et al., 2015), as well as mammalian PICK1 (Karlsen et al., 2015) are all involved in membrane fission. The AHs were suggested to generate membrane curvature and to play a role in membrane fission mediated by endophilins (Gallop et al., 2006; Masuda et al., 2006; Boucrot et al., 2012; Yoon et al., 2012) and amphiphysins (Peter et al., 2004; Boucrot et al., 2012; Yoon et al., 2012; Wu and Baumgart, 2014). Moreover, a higher number of AHs in endophilins compared with amphiphysins was suggested to correlate with increased ability to penetrate the membrane and to drive membrane fission (Boucrot et al., 2012; Yoon et al., 2012). However, other studies question the role of these AHs in membrane curvature generation (e.g., see Chen et al., 2016). The BAR domains were suggested to restrict (Boucrot et al., 2012) or to promote membrane fission (Snead et al., 2019). Finally, Snead et al. (2019) reported that the steric pressure among disordered domains of amphiphysin 1 plays a role in fission. Based on these reports, the molecular mechanism taking place in endophilin- and amphiphysin-induced fission remains to be fully elucidated.

Like AH motif of epsin (Ford et al., 2002), AH motifs of BAR domain-containing fission-inducing proteins endophilins and amphiphysins are also disordered in solution, but become α-helical upon interaction with the membrane (Löw et al., 2008; Jao et al., 2010). In this they differ from the fission-inducing small GTPases Arf1 and Sar1, whose AHs are already formed but are buried in the GDP-bound conformation, and only become exposed upon exchange of GDP to GTP (see above).

Often, endophilins mediate or inhibit fission cooperatively with the proteins belonging to the dynamin superfamily. Endophilin A2 and dynamin contribute to the fission of Shiga-toxin-induced tubules (Renard et al., 2015). In a dynamin-dependent manner, endophilins A2 and A1 mediate clathrin-independent internalization route named FEME (Boucrot et al., 2015). Endophilin A1 inhibits dynamin-mediated membrane fission via intercalation between turns of the dynamin helix (Hohendahl et al., 2017). Endophilin B1 and dynamin 2 cooperatively induce the fission of Golgi membranes during autophagy (Takahashi et al., 2016), while the dynamic clustering of dynamin-amphiphysin 1 rings was suggested to regulate fission of large unilamellar vesicles (Takeda et al., 2018).

Interestingly, predicted curved structure of the ten C-terminal ankyrin repeats of AH-containing fission-inducing protein ANKHD1 is similar to the lipid-binding surface of BAR domain-containing proteins (Kitamata et al., 2019).

### Atg Proteins

Two Atg proteins, Atg2 and Atg18, contain AHs and mediate membrane fission (Gopaldass et al., 2017; Tamura et al., 2017; Kotani et al., 2018). Macroautophagy (hereinafter called autophagy) is a degradation process highly conserved from yeast to mammals (Feng et al., 2014; Knorr et al., 2015; Yu and Melia, 2017; Osawa et al., 2019; and references therein). During autophagy, a bowl-shaped membrane vesicle called phagophore appears, engulfs a part of the cytoplasm (including cytotoxins, damaged organelles, and invasive microbes), and expands. Finally, the open end of phagophore closes, and thus, the inner and outer bilayers become separate entities, and a double-membrane vesicle called autophagosome forms. Closure of the phagophore, leading to the autophagosome formation, is a membrane fission event (Knorr et al., 2015; Yu and Melia, 2017); and, moreover, is a reverse-topology fission event (Schöneberg et al., 2017). Finally, the outer membrane of the autophagosome fuses with a lysosome or vacuole, and captured cytoplasmic contents, together with the inner membrane of the autophagosome, is degraded by hydrolases. The resulting materials, such as amino acids, are released to the cytosol and recycled.

Many Atg proteins are required for the formation of autophagosome. Atg2 is one of these proteins. Two mammalian homologs of yeast Atg2 were identified: Atg2A and Atg2B (Velikkakath et al., 2012). Yeast Atg2 (Kotani et al., 2018) as well as mammalian Atg2 proteins (Velikkakath et al., 2012; Tamura et al., 2017; Tang et al., 2017) play a role in autophagosome formation. Two mammalian Atg2 homologs, Atg2A and Atg2B, have redundant functions in autophagy (Tamura et al., 2017). In the absence of mammalian Atg2A and Atg2B, the closure of autophagosomes is impaired (Velikkakath et al., 2012; Tang et al., 2017), indicating that these proteins are involved in membrane fission event, although other molecules might also be required for this process. Both yeast Atg2 (Kotani et al., 2018) and mammalian Atg2A (Tamura et al., 2017; Chowdhury et al., 2018) contain AHs, and the AH of yeast Atg2 corresponds to the AH of mammalian Atg2A (Kotani et al., 2018). These AHs are required for autophagosome formation (Tamura et al., 2017; Kotani et al., 2018). Mutations of hydrophobic residues of AH from human Atg2A to negatively charged residues abolish membrane-binding capability (Chowdhury et al., 2018) and causes defects in autophagy (Tamura et al., 2017). Similarly, mutations of hydrophobic residues of yeast Atg2 to negatively charged Asp inhibit autophagy (Kotani et al., 2018). From these observations, it derives that yeast and mammalian homologs of Atg2 belong to the family of AH-containing membrane fission-inducing proteins.

Autophagy-related proteins are classified into six functional groups (Velikkakath et al., 2012; Osawa et al., 2019). Atg2 and Atg18 belong to the same group. Atg18 forms a complex with Atg2 and also plays a role in autophagosome formation (Kotani et al., 2018). On top of its role in autophagy, Atg18 also mediates vacuole fission (Gopaldass et al., 2017). Amphipathic α-helical character of the Atg18 segment (residues ∼Pro366-Ser383) is required for Atg18-driven vacuole fission (Gopaldass et al., 2017). Vacuole fission is a normal topology fission event, and, hence, Atg18 is an interesting kind of protein that is involved in fission processes of two opposite topologies: formation of autophagosome (reverse topology) and vacuole fission (normal topology). It is noteworthy that Atg18 displays different requirements in these two fission processes. In vacuole fission, AH is crucial, whereas its binding partner Atg2 is dispensable. In autophagy, interaction of Atg18 with Atg2 is necessary, whereas the Atg18 AH is of moderate importance (Gopaldass et al., 2017). However, the ability of Atg18 to bind phosphatidylinositol 3-phosphate (PtdIns3P) is required for both processes: autophagy (Kotani et al., 2018) and vacuole fission (Gopaldass et al., 2017).

Interestingly, the AH motif of Atg18, similar to the AH motifs of epsins, endophilins and amphiphysins, is unstructured in solution, but transforms into an AH upon membrane binding (Gopaldass et al., 2017). Whereas small GTPases Arf1 and Sar1, ENTH/ANTH domain fission-inducing proteins, endophilins and amphiphysins contain N-terminal AH (see above), AHs of Atg proteins, Atg2 and Atg18, similar to the AH of PICK1, are internal and far, in primary sequence, from the N-terminus.

### Synucleins

Mitochondria are double-membrane-bound organelles that constantly undergo fusion and fission events, referred to as mitochondrial dynamics (Tilokani et al., 2018). Fission of OMM occurs at the ER contact sites. Protein adaptors, such as MFF and MiD49 and MiD51, recruit Drp1, a soluble protein belonging to the dynamin superfamily. Drp1 mediates OMM constriction, whereas final step, namely scission, is mediated by another mechanoenzyme, dynamin2 (Dnm2). Other proteins, such as INF2, Spire1C, actin, and Myosin IIA play a role in this fission process (Pagliuso et al., 2018; Tilokani et al., 2018; and references therein). Simplified model for OMM fission is shown in Figure 4 of Tilokani et al. (2018). Fission of IMM occurs independently of OMM fission (Agrawal and Ramachandran, 2019). Mechanisms regulating constriction of IMM are poorly understood.

Proteins belonging to the synuclein family (Lavedan, 1998), namely α-synuclein (Kamp et al., 2010; Nakamura et al., 2011; O’Donnell et al., 2014; Martinez et al., 2018) and β-synuclein (Nakamura et al., 2011; Taschenberger et al., 2013), are involved in mitochondrial fragmentation. α-Synuclein contains a uniquely long α-11/3 AH that forms upon binding to a lipid bilayer (George et al., 1995; Davidson et al., 1998; Drin and Antonny, 2010; Braun et al., 2017). Similar AHs are present also in β-synuclein and γ-synuclein (Ducas and Rhoades, 2012). β-Synuclein mediates less fragmentation than α-synuclein, whereas γ-synuclein causes very little if any fragmentation (Nakamura et al., 2011). Therefore, in this section we mostly discuss α-synuclein.

α-Synuclein is closely associated with the development of Parkinson’s disease. The function of this protein is not well understood. As already mentioned, α-synuclein drives fragmentation of mitochondria (Kamp et al., 2010; Nakamura et al., 2011; O’Donnell et al., 2014; Martinez et al., 2018). Uncontrolled mitochondrial fragmentation might contribute to the development of Parkinson’s disease (Varkey et al., 2010; Panchal and Tiwari, 2019) and, importantly, duplication and triplication of *SNCA* (α-synuclein) gene cause a severe form of this disease (Olgiati et al., 2015; Konno et al., 2016). The authors of Kamp et al. (2010) and Nakamura et al. (2011) hypothesized that fragmentation of mitochondria might be independent of Drp1, whereas Martinez et al. (2018) reported that Drp1 is required for α-synuclein-mediated mitochondrial fragmentation. α-Synuclein might drive fragmentation of mitochondria by decreasing the rate of fusion or, alternatively, by promoting fission (Nakamura, 2013). Kamp et al. (2010) hypothesized that the influence of this protein on mitochondrial dynamics is based on inhibition of fusion, whereas Nakamura et al. (2011) suppose that, quite opposite, α-synuclein acts by promoting membrane fission. The latter hypothesis is supported by the fact that α-synuclein is able to convert large vesicles (liposomes) into smaller vesicles, i.e., mediate membrane fission (Varkey et al., 2010; Nakamura et al., 2011; Fakhree et al., 2019). AH of α-synuclein contributes to the generation of membrane curvature (Braun et al., 2014, 2017; Fakhree et al., 2019). Thus, it is reasonable to hypothesize that α-synuclein and, possibly, also β-synuclein belong to the large family of AH-containing fission-inducing proteins.

In summary, in most AH-containing fission-inducing proteins AHs were shown or suggested to play an important role in various fission events. As indicated, the AH penetration in the lipid bilayer can be the starting point of the fission process, followed by the recruitment of other proteins that mediate the final fission event (Sundborger et al., 2011; Meinecke et al., 2013). Shallow insertion of AHs into the membrane was suggested to drive membrane fission (Boucrot et al., 2012). However, few recent publications of Jeanne Stachowiak’s group consider also an alternative mechanism foreseeing that the steric pressure generated by bulky disordered domains can drive membrane fission (Snead et al., 2017, 2019; Snead and Stachowiak, 2018), whereas the role of insertions, such as AHs, is to anchor proteins to the membrane (Snead et al., 2017). Whether the different mechanisms coexist or one prevails over the others remains to be defined.

## The Role of Lipid Cofactors in Membrane Fission Driven by Amphipathic Helix-Containing Proteins

### Lipid Cofactors: An Overview

**FIGURE 3:**
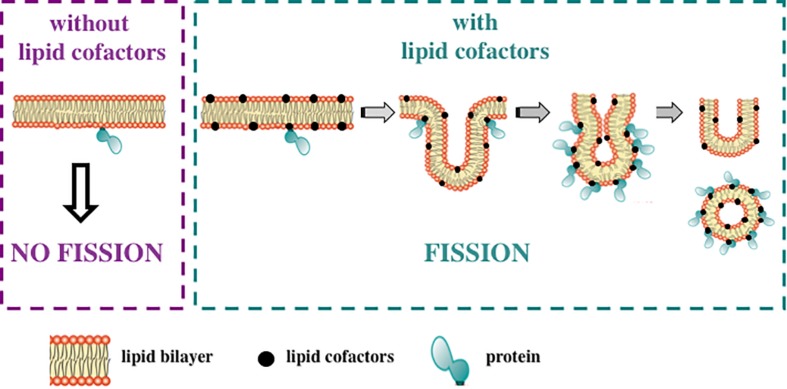
Lipid cofactors are required for fission-inducing proteins. Many AH-containing and AH-free fission-inducing proteins need specific lipids, named lipid cofactors, to promote and complete membrane fission. Thus, the ability of the fission-inducing proteins to support this process is strictly dependent on membrane lipid composition. Of note, proteins are not able to induce membrane fission in the absence of these lipids (left panel; purple dashed line). Conversely, in the presence of such lipid cofactors, fission occurs through the following steps: membrane neck, hemifission and formation of two separate membranes (right panel; green dashed line). Lipid cofactors include: PtdIns(4,5)P_2_, PA, cardiolipin, MMPE, PtdIns3P, PtdIns(3,5)P_2_ and cholesterol. See text for details.

Many AH-containing fission-inducing proteins require one or two specific lipids to generate membrane curvature and to complete the fission process (Figure 3). Most of these lipids are anionic. Such lipids could be named lipid cofactors (Ramachandran, 2018), or lipid factors (Danne et al., 2017b), or lipid ligands (Gopaldass et al., 2017). Examples of lipid cofactors of AH-containing fission proteins are: (1) PtdIns(4,5)P_2_ for epsin (Kweon et al., 2006; Boucrot et al., 2012; Lai et al., 2012), mammalian amphiphysin 2 (Lee et al., 2002; Wu and Baumgart, 2014), *Drosophila* amphiphysin (Yoon et al., 2012), and mammalian endophilin A1 (Yoon et al., 2012); (2) cardiolipin and MMPE for PmtA (Danne et al., 2017b); (3) PtdIns3P and PtdIns(3,5)P_2_ for Atg18 (Gopaldass et al., 2017; Scacioc et al., 2017); (4) cholesterol for M2 protein from influenza A virus (Ekanayake et al., 2016; Elkins et al., 2017, 2018; Herneisen et al., 2017; Pan et al., 2019; and references therein), see Table 2.

**TABLE 2:** Examples of lipid cofactors of fission-inducing proteins containing amphipathic helices.

**Protein**	**Lipid cofactors**	**Membrane interaction**	**Generation of membrane curvature**	**Topology of fission**
Epsin	PtdIns(4,5)P_2_	H0 of epsin interacts with PtdIns(4,5)P_2_- containing, but not with PS-containing liposomes (Kweon et al., 2006)	Vesiculation occurs on PtdIns(4,5)P_2_-containing membranes but not on PS-containing membranes (Boucrot et al., 2012)	Normal
Mammalian amphiphysin 2	PtdIns(4,5)P_2_	Binds specifically to liposomes containing PtdIns(4,5)P_2_ and to lesser extent PtdIns4P, but not PS, PtdIns, PtdIns(3,4)P_2_, PtdIns(3,5)P_2_ (Lee et al., 2002)	Tubulation and vesiculation were observed on PtdIns(4,5)P_2_-containing liposomes, but to a much lesser extent on PtdIns(4,5)P_2_-free liposomes (Wu and Baumgart, 2014)	Normal
*Drosophila* amphiphysin	PtdIns(4,5)P_2_	Binds specifically to vesicles containing PtdIns(4,5)P_2_ and PtdIns(3,4,5)P_3_ but not other PtdInsPs (Yoon et al., 2012)	Tubulation requires PtdIns(4,5)P_2_, but not PS (Yoon et al., 2012)	Normal
Endophilin A1	PtdIns(4,5)P_2_	Binds specifically to vesicles containing PtdIns(4,5)P_2_ and PtdIns(3,4,5)P_3_, but not other PtdInsPs (Yoon et al., 2012)	Tubulation requires PtdIns(4,5)P_2_, but not PS (Yoon et al., 2012)	Normal
AtPmtA (bacterial)	CL and MMPE	Binds to cardiolipin-containing liposomes, but poorly to PA-containing liposomes (Danne et al., 2017b)	Tubulation was observed on CL-containing liposomes, but with less efficacy on PG-, PtdIns4P-, and PA-containing liposomes. The addition of MMPE to CL-containing liposomes bound to PmtA promotes vesiculation (Danne et al., 2017b)	Normal
Atg18 from yeast	PtdIns3P and PtdIns(3,5)P_2_	Binds to PtdIns3P-containing and PtdIns(3,5)P_2_-containing liposomes. Does not bind to PtdIns(4,5)P_2_-containing or PS-containing liposomes (Gopaldass et al., 2017)	Vesiculation occurs on GUVs containing PtdIns3P and PtdIns(3,5)P_2_. Scission was not observed on GUVs containing PtdIns(4,5)P_2_ (Gopaldass et al., 2017)	Normal
M2 from influenza virus	Cholesterol	M2 binds cholesterol specifically (Ekanayake et al., 2016; Elkins et al., 2017, 2018)	Cholesterol significantly augmented the capability of the M2 amphipathic helix (M2AH) in inducing bilayer pits. POPG does not have the ability of significantly impacting M2AH-induced membrane modulation (Pan et al., 2019)	Reverse

*The indicated lipids are specifically required for membrane fission reactions mediated by these proteins. Other lipids cannot replace these lipid cofactors to generate membrane curvature (i.e., tubulation and vesiculation). The topology of fission reaction for each protein is also shown.*

The lipid cofactor-binding sites were found in the AHs of few fission-inducing proteins: PtdIns(4,5)P_2_-binding site in epsin (Kweon et al., 2006; Lai et al., 2012), site that binds PtdIns(3,5)P_2_ and PtdIns3P in Atg18 (Gopaldass et al., 2017), and cholesterol-binding site in M2 (Elkins et al., 2017, 2018). It is not surprizing that anionic lipid-binding sites contain basic residues: Arg7, Arg8, Lys11 of epsin belong to the PtdIns(4,5)P_2_-binding site, whereas Arg285 and Arg286 of Atg18 are involved in the interaction with PtdIns(3,5)P_2_ and PtdIns3P. Accordingly, other fission-inducing proteins may contain few basic residues as anionic lipid cofactor-binding sites that are located close to each other in primary sequence.

In some membrane fission reactions involving AH-containing proteins, the role of specific lipids is not yet fully established. Possibly, cardiolipin is a cofactor for membrane fission mediated by Pex11 [peroxisome fission (Opaliński et al., 2011; Su et al., 2018)]; bacterial MGS (Eriksson et al., 2009; Ariöz et al., 2013), DGS (Edman et al., 2003; Eriksson et al., 2009), and MurG (van den Brink-van der Laan et al., 2003; Lind et al., 2007) proteins; and by MinD [bacterial division (Zhou and Lutkenhaus, 2003; Renner and Weibel, 2012)]. PA might be a lipid cofactor for endophilin B1 (also known as Bif-1) (Zhang et al., 2011) that contains two AHs (Kjaerulff et al., 2011) and plays a role in membrane fission (Rostovtseva et al., 2009; Takahashi et al., 2011, 2016). Possibly, PtdIns(4,5)P_2_ is a lipid cofactor in membrane vesiculation mediated by PICK1 (Pan et al., 2007; Karlsen et al., 2015). PS might be a lipid cofactor in the fission reaction mediated by ANKHD1 (Kitamata et al., 2019). Arf1 binds to PtdIns(4,5)P_2_ and PA (Randazzo, 1997; Manifava et al., 2001; Krauss et al., 2008), but the role of these interactions in Arf1-mediated membrane fission is not completely clear.

As we already mentioned, an AH-containing protein α-synuclein induces fragmentation of mitochondria (Kamp et al., 2010; Nakamura et al., 2011; O’Donnell et al., 2014; Martinez et al., 2018) and converts large vesicles (liposomes) into smaller vesicles (Varkey et al., 2010; Nakamura et al., 2011; Fakhree et al., 2019). Moreover, AH plays a role in the α-synuclein-driven generation of membrane curvature (Braun et al., 2014, 2017; Fakhree et al., 2019). Hence, we can consider α-synuclein as an AH-containing fission-inducing protein. Interestingly, α-synuclein binds to cardiolipin (Nakamura et al., 2008; Robotta et al., 2014; Ryan et al., 2018) and PA (Perrin et al., 2000; Nakamura et al., 2011; Mizuno et al., 2017) with high affinity. Moreover, this protein prefers negatively charged lipids cardiolipin (Nakamura et al., 2008) and PA (Nakamura et al., 2008; Mizuno et al., 2017) to another negatively charged lipid PS. The cone shape of cardiolipin, whose tail is wider than its headgroup (see below), might contribute to the affinity of α-synuclein to cardiolipin-containing bilayers (Ghio et al., 2019; and references therein). PA (Mizuno et al., 2017) and cardiolipin (Ryan et al., 2018) promote the formation of the AH of α-synuclein. Importantly, α-synuclein mediates fission of liposomes that contain both PC and cardiolipin, but not liposomes that contain only PC (Nakamura et al., 2011). We propose that cardiolipin and, possibly, also PA are cofactors in membrane fission mediated by AH-containing protein α-synuclein.

To the best of our knowledge, membrane fission mediated by small GTPase Sar1 does not need any lipid cofactors. It has been stressed that Arf1 and Sar1 use the energy of GTP hydrolysis, whereas no enzymatic activity has been found or predicted for Atg18 (Gopaldass et al., 2017). However, Atg18 membrane recruitment and oligomerization needs PtdIns3P and PtdIns(3,5)P_2_ as lipid cofactors (see Table 2), and synthesis of these lipids is energy-consuming. Hence, this fission process can be energized indirectly, via the energy invested into the synthesis of lipid cofactors (Gopaldass et al., 2017). Possibly, such indirect driving force of the reaction can also be considered for other AH-containing fission proteins.

Anionic lipid cofactors cannot be replaced with other anionic lipids: see Table 2. Tubulation mediated by endophilin A1 requires PtdIns(4,5)P_2_ but not PS (Yoon et al., 2012). PmtA tubulates liposomes containing cardiolipin, but is less efficient in tubulating liposomes containing PG, PtdIns4P or PA (Danne et al., 2017b). The AH-containing fission proteins epsin (Boucrot et al., 2012), PmtA (Danne et al., 2017b), Atg18 (Gopaldass et al., 2017) mediate fission of membranes containing their lipid cofactors, but they are inefficient with membranes containing other acidic lipids.

Electrostatic interaction between positively charged residues belonging to AH and negatively charged phospholipids was proposed to overcome the energetic cost of spreading lipids apart and thus to play an important role in the generation of membrane curvature needed for the fission event (Khattree et al., 2013; Roberts et al., 2013; Brady et al., 2015). Most probably, anionic lipid cofactors are required for the recruitment of AH-containing fission proteins. Furthermore, as pointed out above, the shallow penetration of some AHs leads to the generation of local membrane curvature through the pushing of the lipid headgroups apart (the “wedge” mechanism) (McMahon and Gallop, 2005; Campelo et al., 2008; Drin and Antonny, 2010; Baumgart et al., 2011; Lai et al., 2012; McMahon and Boucrot, 2015; and references therein). At least in some cases, the depth of the AH insertion into the membrane depends on the presence of negatively charged lipids in the membrane (Sani et al., 2012; Palomares-Jerez et al., 2013). Indeed, the interaction of curvature-inducing AHs with anionic lipids might contribute to the optimal depth of AH insertion.

As pointed out above, the presence of anionic lipids in the membrane often promotes the folding of AH (Davidson et al., 1998; Kweon et al., 2006; Fernandes et al., 2008; Horchani et al., 2014; Brady et al., 2015; Mizuno et al., 2017; Ryan et al., 2018). Thus, another mode for the anionic lipid to mediate membrane curvature generation and promote membrane fission is facilitating the folding of AH motif, once the protein segment comes to close contact with the membrane.

Simple electrostatic attraction between acidic lipids and basic residues of AHs does not explain the specificity of the action of lipid cofactors on fission proteins. AH-containing fission proteins epsin (Kweon et al., 2006), endophilin A1 (Yoon et al., 2012), amphiphysin 2 (Lee et al., 2002), PmtA (Danne et al., 2017b), Atg18 (Gopaldass et al., 2017) interact specifically with the membranes containing their lipid cofactors, but poorly with the membranes containing other anionic lipids. For example, PmtA binds cardiolipin-containing liposomes, but poorly binds PA-containing liposomes (Danne et al., 2017b). Most probably, a specific interaction of fission proteins with their lipid cofactors is necessary for the correct conformation and oligomeric structure of these proteins.

Reverse-topology membrane fission-inducing protein M2 from influenza A virus contains an AH, but uses neutral cholesterol as a cofactor in the membrane fission process (Elkins et al., 2017; Herneisen et al., 2017; Pan et al., 2019; and references therein), see Table 2. However, we should keep in mind that in the case of reverse topology fission, AHs insert into the proximal leaflet of the budding membrane-enclosed compartment, and not into the distal leaflet, as in the case of the normal-topology fission. Hence, the process of membrane remodeling is fundamentally different, and the requirements for the lipid cofactor might also be different.

Cholesterol may control the insertion depth of the M2 AH in the bilayer (Kim et al., 2015; Herneisen et al., 2017), thus, contributing to the optimal depth of the AH insertion, as we have discussed above for the interaction of the AHs of other fission-inducing proteins with their anionic lipid cofactors. Moreover, cholesterol was suggested to promote a more compact conformation of the M2 AH (Herneisen et al., 2017). This protein was shown also to cluster at the phase boundary of cholesterol-rich liquid-ordered phase and cholesterol-poor liquid-disordered phase (Roberts et al., 2013). Such a localization of a fission-inducing protein at the neck of the budding virus can generate the membrane curvature required for membrane fission (Elkins et al., 2017; Martyna et al., 2017). A similar mechanism might be applicable for few other membrane fission processes mediated by AH-containing proteins interacting with specific lipid cofactors.

Some fission-inducing proteins, in which no definitely established AHs have yet been identified, also require lipid cofactors for their activity. For example, PA is a lipid cofactor for CtBP1-S/BARS (Weigert et al., 1999; Pagliuso et al., 2016); PS is a lipid cofactor for Ebola virus VP40 (Soni and Stahelin, 2014; Adu-Gyamfi et al., 2015; Del Vecchio et al., 2018); PtdIns(4,5)P_2_ is a putative lipid cofactor for dynamin (Jost et al., 1998); cardiolipin is a probable lipid cofactor for FisB (Doan et al., 2013) and, moreover, activates mechanoenzyme Drp1 that plays a role in mitochondrial fission (Francy et al., 2017).

Lipid cofactors should not be confused with lipids that just recruit fission-inducing proteins to the membrane, but are not required for the fission reaction *per se*. Unlike lipids that just recruit proteins, lipid cofactors are indispensable for fission: membrane curvature generation cannot proceed efficiently without them, see Table 2. For example, PtdIns4P was suggested to have a direct role in the anchoring of the protein complex-bound CtBP1-S/BARS (Valente et al., 2013). However, only PA, but not PtdIns4P, allowed CtBP1-S/BARS to induce liposome tubulation (Yang et al., 2008). Hence, PA, but not PtdIns4P, can be considered a lipid cofactor in CtBP1-S/BARS-mediated fission. However, often the information concerning particular lipid is incomplete. In this case, it is difficult to figure out if this lipid a true cofactor or protein-recruiting lipid.

Interestingly, two fission-inducing proteins, *A. tumefaciens* PmtA and mammalian CtBP1-S/BARS, are known to play a role in the synthesis of their own lipid cofactors. The difference between PmtA and CtBP1-S/BARS is that PmtA is itself an enzyme that produces its own lipid cofactor MMPE (Danne et al., 2017b), whereas CtBP1-S/BARS binds to and activates an enzyme, LPAATδ that catalyzes the synthesis of PA required for CtBP1-S/BARS-mediated membrane fission (Pagliuso et al., 2016; Zhukovsky et al., 2019b). Such production of lipid cofactors promoted by the very fission-inducing proteins and then using them guarantees availability of these lipids where and when they are needed.

### Cardiolipin, PA, and PtdIns(4,5)P_2_ Act as Lipid Cofactors in Several Membrane Fission Reactions

We noted that the three negatively charged lipids PtdIns(4,5)P_2_, cardiolipin, and PA might play a role of lipid cofactors in several fission reactions mediated by different proteins. Cardiolipin is an unusual phospholipid that consists of two PA molecules connected with a glycerol backbone and thus contains two phosphates and four fatty acid tails. Cardiolipin is present in the membranes of most bacteria, whereas in mammalian and plant cells it is found mostly in the IMM and, to a lesser extent, in the OMM and in peroxisomes (Wriessnegger et al., 2007; Musatov and Sedlak, 2017; Pagliuso et al., 2018). PA containing a unique phosphomonoester headgroup is a minor phospholipid present in various organelles (Zhukovsky et al., 2019a). PtdIns(4,5)P_2_ is enriched in the PM of eukaryotic cells (Olivença et al., 2018; Dickson and Hille, 2019).

The role that cardiolipin and PA play in membrane fission reactions might be determined by their structure, namely combination of charge and shape (Danne et al., 2017b; Agrawal and Ramachandran, 2019; Zhukovsky et al., 2019a). Packing defects are locations on the membrane surface where the hydrophobic interior of the membrane is exposed to the solvent. AHs have a tendency to bind such defects induced by cone-shaped lipids, i.e., lipids whose tails are wider than their headgroups (Ouberai et al., 2013; Vanni et al., 2013; Lauwers et al., 2016). At physiological conditions, cardiolipin carries a charge −2 (Arnarez et al., 2016; Sathappa and Alder, 2016; Boyd et al., 2018; and references therein) and, moreover, has a cone shape (Arnarez et al., 2016; Carranza et al., 2017; Basu Ball et al., 2018; Su et al., 2018; and references therein). PA also has a cone shape (Kooijman et al., 2005). According to the electrostatic/hydrogen bond switch mechanism, the PA charge can change from −1 to −2 upon interaction with Lys or Arg (Kooijman et al., 2007). Hence, cone-shaped lipid PA, as cone-shaped lipid cardiolipin, is able to have charge −2. Combination of double negative charge and cone shape of cardiolipin and PA allows positively charged protein segments, such as AHs with basic residues on the hydrophilic face, to interact with the membranes containing these lipids. The combination of net charge and cone shape of cardiolipin seems to be responsible for the membrane-remodeling activity of PmtA (Danne et al., 2017b).

PtdIns(4,5)P_2_, like cardiolipin and PA, is a lipid cofactor in membrane fission reactions mediated by various proteins, such as epsin (Kweon et al., 2006; Boucrot et al., 2012; Lai et al., 2012), mammalian amphiphysin 2 (Lee et al., 2002; Wu and Baumgart, 2014), *Drosophila* amphiphysin (Yoon et al., 2012) and mammalian endophilin A1 (Yoon et al., 2012). Like cardiolipin and PA, PtdIns(4,5)P_2_ possesses a high negative charge. The charge of PtdIns(4,5)P_2_ depends on various factors, but at the physiological conditions, the charge of this lipid is approximately −4 (McLaughlin et al., 2002; Heo et al., 2006; Kooijman et al., 2009; and references therein). However, unlike cardiolipin and PA that have a cone shape, PtdIns(4,5)P_2_ and other phosphoinositides have an inverted-cone shape, i.e., their headgroups are wider than their tails (Martin, 2012; Suetsugu et al., 2014; McMahon and Boucrot, 2015; and references therein). Besides PtdIns(4,5)P_2_, other phosphoinositides also function as lipid cofactors in membrane fission processes, e.g., PtdIns3P and PtdIns(3,5)P_2_ for Atg18 (Gopaldass et al., 2017). We hypothesize that, despite of their shape, PtdIns(4,5)P_2_ and PtdIns(3,5)P_2_ are able to be lipid cofactors required for membrane fission reactions because of extremely high negative charge that recruits basic residues of the AHs of fission-inducing proteins to PBP-containing membranes due to the electrostatic attractions. If a PBP-containing membrane contains also cone-shaped lipids, membrane packing defects are present in such membrane, and some of these defects are present in the vicinity of PBP molecules. If a membrane contains cone-shaped lipids, membrane packing defects should be evenly distributed on the membrane surface (Vamparys et al., 2013). Co-localization of some of these defects with highly charged PBPs creates suitable condition for the recruitment of AHs belonging to the fission-inducing proteins.

Alternatively, we hypothesize that, while AH-containing fission-inducing proteins interacting with cardiolipin and PA play a major role in fission, AH-containing proteins that bind PtdIns(4,5)P_2_ often mediate membrane fission cooperatively with the proteins belonging to the dynamin superfamily (Boucrot et al., 2012, 2015; Meinecke et al., 2013). Possibly, interaction with cone-shaped lipids, such as cardiolipin and PA, is required for AH-containing proteins that mediate fission in the absence of proteins belonging to the dynamin superfamily, but is not required for AH-containing proteins that mediate fission cooperatively with the proteins belonging to this superfamily.

Of note, PS, phosphatidylinositol (PtdIns) and PG that possess net charge −1 at neutral pH (Li et al., 2015) are less frequently used as lipid cofactors in membrane fission processes, compared with PA, cardiolipin and PtdIns(4,5)P_2_ that possess higher negative charge.

### Putative Sequence Motifs That Specifically Recognize Cardiolipin, PA, and PtdIns(4,5)P_2_

It is reasonable to hypothesize that AHs of proteins interacting specifically with each of three lipids cardiolipin, PA, PtdIns(4,5)P_2_, might contain sequence motifs that recognize these lipids. Primary sequences of the AHs of fission-inducing proteins that were shown or suggested to use cardiolipin and PtdIns(4,5)P_2_ as cofactors are presented in Tables 3, 4. We found that (K/R)x_6_(F/Y) motif is present in all but one AHs of cardiolipin-binding fission-inducing proteins we know, whereas (K/R/H)(K/R/H)xx(K/R/H) motif is present in most AHs of PtdIns(4,5)P_2_-binding fission-inducing proteins we know. Here K is Lys, R is Arg, F is Phe, Y is Tyr, H is His, and x is any residue. Although it is quite possible that the (K/R)x_6_(F/Y) and (K/R/H)(K/R/H)xx(K/R/H) motifs in some of AHs of fission-inducing proteins are not involved in the specific interaction with lipids, we suppose that in most cases these two motifs are functionally essential.

**TABLE 3:** Amphipathic helices of fission-inducing proteins that use cardiolipin as a lipid cofactor.

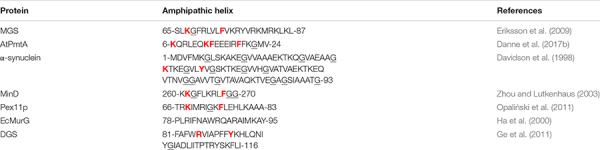

*Sequences are retrieved from indicated references. Residues belonging to the (K/R)x_6_(F/Y) motif are shown in red. Gly are underlined.*

**TABLE 4:** Amphipathic helices of fission-inducing proteins that use PtdIns(4,5)P_2_ as a lipid cofactor.

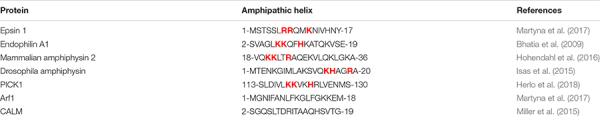

*Sequences are retrieved from indicated references. Residues belonging to the (K/R/H)(K/R/H)xx(K/R/H) motif are shown in red.*

#### Cardiolipin

Among proteins that are involved in membrane fission and are listed in the Tables 3, 4, the (K/R)x_6_(F/Y) motif is present in AHs of six out of seven cardiolipin-binding proteins, but absent in AHs of all seven PtdIns(4,5)P_2_-binding proteins. In general, (K/R)x_6_(F/Y) motifs in the AHs of various proteins are rare. Among 16 AHs shown in the Table 1 of Drin and Antonny (2010), this motif is present only in α-synuclein, a protein that binds cardiolipin (Nakamura et al., 2008; Robotta et al., 2014; Ryan et al., 2018). However, except proteins listed in the Table 3, this motif is present in the AHs of at least two proteins that interact with cardiolipin but, to the best of our knowledge, are not known to be involved in membrane fission: PmtA from *Bradyrhizobium japonicum* (Danne et al., 2017a) and ATG3 (Nath et al., 2014; Hervás et al., 2017). Arg14 and Phe21 belong to the (K/R)x_6_(F/Y) motif in the AH of PmtA from *B. japonicum* (Danne et al., 2017a), whereas Lys11 and Tyr18 belong to the (K/R)x_6_(F/Y) motif in the AH of Atg3 (Hervás et al., 2017). In the AH of cardiolipin-binding fission-inducing protein PmtA from *A. tumefaciens* (Danne et al., 2017b), both Lys12 and Phe19 belonging to the Kx_6_F motif are critical for membrane binding (Danne et al., 2015). Based on these observations, we make a conclusion that, most probably, many (K/R)x_6_(F/Y) pairs in the AHs of cardiolipin-binding proteins are functional cardiolipin-binding motifs.

**FIGURE 4:**
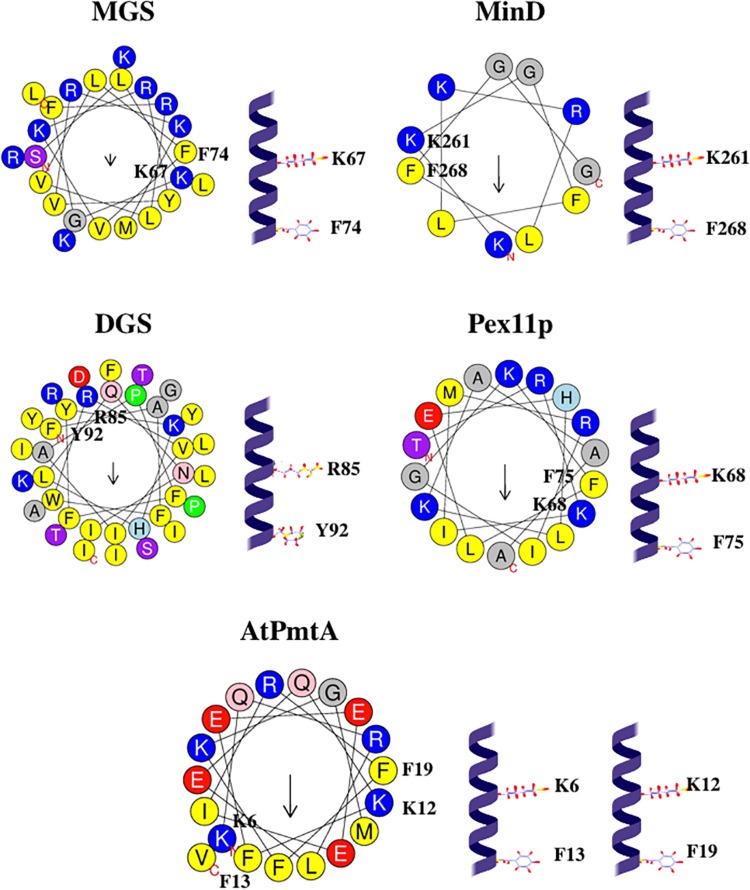
Configurations and helical wheel representations of some of the (K/R)x_6_(F/Y) motif-containing AHs belonging to the fission-inducing proteins that use cardiolipin as cofactor. Helical wheel projections were generated using Heliquest software (http://heliquest.ipmc.cnrs.fr; Gautier et al., 2008). Residues belonging to the (K/R)x_6_(F/Y) motifs are highlighted. Color coding for residues: yellow for hydrophobic, purple for Ser (S) and Thr (T), blue for Lys (K) and Arg (R), red for acidic, pink for Asn (N) and Gln (Q), gray for small residues (Ala, A and Gly, G), green for Pro (P), and light blue for His (H). The arrow in helical wheels corresponds to the hydrophobic moment. Species names and UniProt accession numbers: *Acholeplasma laidlawii* MGS, Q93P60; *Escherichia coli* MinD, P0AEZ3; *A. laidlawii* DGS, Q8KQL6; *Penicillium chrysogenum* Pex11p, B6GZG8; *Agrobacterium tumefaciens* AtPmtA, A0A2L2L7Q9.

In the protein α-helix, each amino acid residue corresponds to a 100° turn in the helix. (K/R)x_6_(F/Y) motif contains one basic residue (Arg or Lys) and one aromatic residue (Phe or Tyr) spaced roughly 700° in the circle, very close to 720° (two turns). Hence, basic residue and aromatic residue of the (K/R)x_6_(F/Y) motif are on the same face of the helix, two turns apart, see Figure 4. Such localization might be suitable for two residues to interact with the same cardiolipin molecule.

Kx_6_F motifs in MGS and MinD contain positively charged Lys followed by Gly. Interestingly, a positively charged residue preceding flexibility-conferring Gly may be considered a footprint for cardiolipin binding (Planas-Iglesias et al., 2015).

In (K/R)x_6_(F/Y) motifs within AHs of cardiolipin-binding proteins, Gly is often present close to basic (Lys or Arg) and aromatic (Phe or Tyr) residues, see Table 3. Hence, in most (K/R)x_6_(F/Y) motifs, three residues are present: positively charged residue (Lys or Arg), aromatic residue (Phe or Tyr), and Gly. Such a combination of these three kinds of residues is somewhat similar to the cardiolipin-binding motif (Y/W/F)(K/R)G (Ruprecht et al., 2014; Kunji et al., 2016; Duncan et al., 2018), where W is Trp and G is Gly. We suppose that, as suggested for the (Y/W/F)(K/R)G motif, aromatic residues (Phe or Tyr) in (K/R)x_6_(F/Y) motif might be involved in the interaction with acyl chain (Ruprecht et al., 2014), whereas neighboring Gly might be involved in the interaction with phosphate groups (Kunji et al., 2016; Duncan et al., 2018).

#### PtdIns(4,5)P_2_

Lys, Arg, and His are basic amino acid residues. Hence, all three residues belonging to the putative PtdIns(4,5)P_2_-binding (K/R/H)(K/R/H)xx(K/R/H) motif are positively charged. This motif is present in five out of seven AHs of fission-inducing PtdIns(4,5)P_2_-binding proteins we know, but in AHs of only two out of seven cardiolipin-binding fission-inducing proteins we know, see Tables 3, 4. AH of Arf1 does not contain (K/R/H)(K/R/H)xx(K/R/H) motif: see Table 4. However, if we consider Arg19 adjacent to this AH, we find that Arf1 contains a K^15^K^16^xxR^19^ sequence that conforms to the (K/R/H)(K/R/H)xx(K/R/H) motif. Double mutation of two Lys belonging to this K^15^K^16^xxR^19^ motif to Leu affected Arf1 binding to PtdIns(4,5)P_2_ but not to PA (Randazzo, 1997). Moreover, (K/R/H)(K/R/H)xx(K/R/H) motifs are present in the AHs of at least three PtdIns(4,5)P_2_-binding proteins that, to the best of our knowledge, are not known to be involved in membrane fission: Spo20 (Horchani et al., 2014), STIM2 (Bhardwaj et al., 2013), and SH3YL1 (Hasegawa et al., 2011). His75, Lys76, and His79 belong to the (K/R/H)(K/R/H)xx(K/R/H) motif in the AH of Spo20 (Horchani et al., 2014); K^732^K^733^PSK^736^ and K^742^K^743^KSK^746^ are two (K/R/H)(K/R/H)xx(K/R/H) motifs in the AH of STIM2 (Bhardwaj et al., 2013), whereas Lys14, Lys15, and Lys18 belong to the (K/R/H)(K/R/H)xx(K/R/H) motif in the AH of SH3YL1 (Hasegawa et al., 2011). Residues belonging to the (K/R/H)(K/R/H)xx(K/R/H) motif within AHs play a role in the interaction with PtdIns(4,5)P_2_: Arg7, Arg8, Lys11 in epsin 1 (Ford et al., 2002; Lai et al., 2012) and Lys14, Lys15, Lys18 in SH3YL1 (Hasegawa et al., 2011). We make a conclusion that, most probably, many (K/R/H)(K/R/H)xx(K/R/H) motifs are functional PtdIns(4,5)P_2_-binding motifs in the AHs of various proteins that interact with this lipid.

**FIGURE 5:**
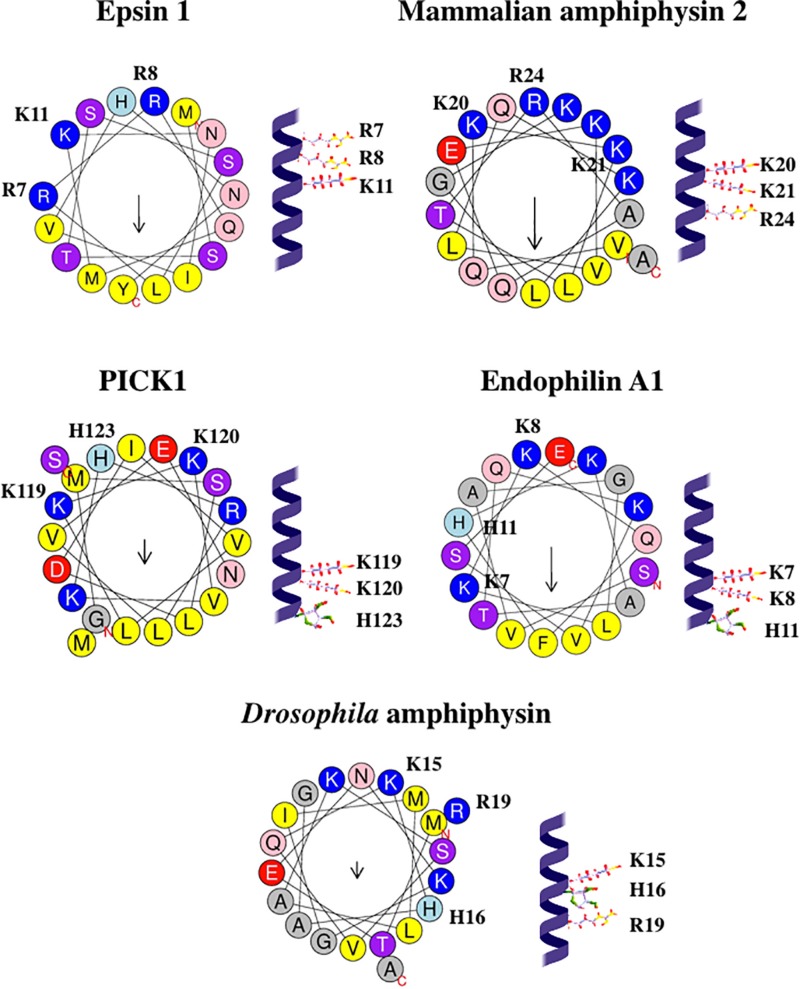
Configurations and helical wheel representations of the (K/R/H)(K/R/H)xx(K/R/H) motif-containing AHs belonging to the fission-inducing proteins that use PtdIns(4,5)P_2_ as cofactor. Helical wheel projections were generated using Heliquest software (http://heliquest.ipmc.cnrs.fr; Gautier et al., 2008). Residues belonging to the (K/R/H)(K/R/H)xx(K/R/H) motifs are highlighted. Color coding for residues: yellow for hydrophobic, purple for Ser (S) and Thr (T), blue for Lys (K) and Arg (R), red for acidic, pink for Asn (N) and Gln (Q), gray for small residues (Ala, A and Gly, G), green for Pro (P), and light blue for His (H). The arrow in helical wheels corresponds to the hydrophobic moment. Species names and UniProt accession numbers: *Homo sapiens* epsin 1, Q9Y6I3; *Rattus norvegicus* amphiphysin 2, O08839; *R. norvegicus* PICK1, Q9EP80; *R. norvegicus* endophilin A1, O35179; *Drosophila melanogaster* amphiphysin, Q7KLE5.

Taking into account that each residue corresponds to a 100° turn in the protein α-helix, all three basic residues belonging to the (K/R/H)(K/R/H)xx(K/R/H) motif are located on the same face of the helix: see Figure 5. Such localization might be favorable for the interaction of these residues with the same PtdIns(4,5)P_2_ molecule. Possibly, in the (K/R/H)(K/R/H)xx(K/R/H) motifs of various proteins, two adjacent positively charged residues interact with 1- and 4-phosphate groups of PtdIns(4,5)P_2_, similar to the two Arg belonging to the R^7^R^8^xxK^11^ motif in epsin 1 (Lai et al., 2012).

According to Nishimura et al. (2018), the BAR domain-containing proteins do not have any specific phosphoinositide-binding pockets. However, we hypothesize that (K/R/H)(K/R/H)xx(K/R/H) motif is a specific PtdIns(4,5)P_2_-binding motif in AHs (and, perhaps, other segments) of various proteins, including BAR domain-containing proteins. We predict that residues Lys7, Lys8, His11 in mammalian endophilin A1, residues Lys20, Lys21, Arg24 in mammalian amphiphysin 2, residues Lys15, His16, Arg19 in *Drosophila* amphiphysin are involved in the interaction with PtdIns(4,5)P_2_. We expect that mutations involving these residues will inhibit such interaction as well as membrane curvature generation and membrane fission mediated by these proteins.

#### Phosphatidic Acid

At least eight AHs of PA-binding proteins contain KxK (Lys – any residue – Lys) motifs, see Table 5. One of these proteins, α-synuclein, might mediate membrane fission (see above), whereas other seven proteins, Atg3 (Nath et al., 2014; Hervás et al., 2017), CDeT11-24 (Petersen et al., 2012), Opi1 (Loewen et al., 2004; Hofbauer et al., 2018), Spo20 (Horchani et al., 2014), DHN1 (Koag et al., 2003, 2009), CCT also known as Pcyt1a (Cornell, 2016), and Tam41 (Jiao et al., 2019), are not known to be involved in fission (to the best of our knowledge). In Atg3, residues Lys9 and Lys11, belonging to the KxK motif, are essential for membrane interaction (Hervás et al., 2017). In Opi1, mutation of five Lys (including Lys119 and Lys121 belonging to the KxK motif) to Arg leads to the loss of the preference for PA-containing over PS-containing liposomes (Hofbauer et al., 2018).

**TABLE 5:** Amphipathic helices of PA-binding proteins that contain KxK motifs.

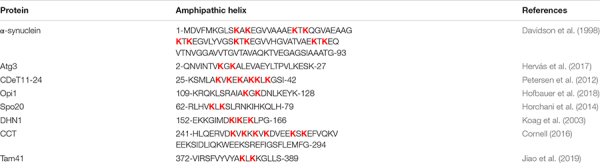

*Only one protein in this Table (α-synuclein) was suggested to mediate membrane fission. Sequences are retrieved from indicated references. Residues belonging to the KxK motif are shown in red.*

Amphipathic helices of few PA-binding proteins, including fission-inducing proteins endophilin B1 (Rostovtseva et al., 2009; Kjaerulff et al., 2011; Zhang et al., 2011) and Arf1 (Krauss et al., 2008; Beck et al., 2011; Martyna et al., 2017) do not contain KxK motifs. Moreover, the authors of Putta et al. (2016), Kassas et al. (2017), Zegarlińska et al. (2018), and Tanguy et al. (2019) stress that there is no known well-defined PA-binding motif or domain. However, here we hypothesize that KxK motif in AHs is involved in specific interaction with PA. It is possible that other sequence motifs recognizing PA will be discovered in the future.

**FIGURE 6:**
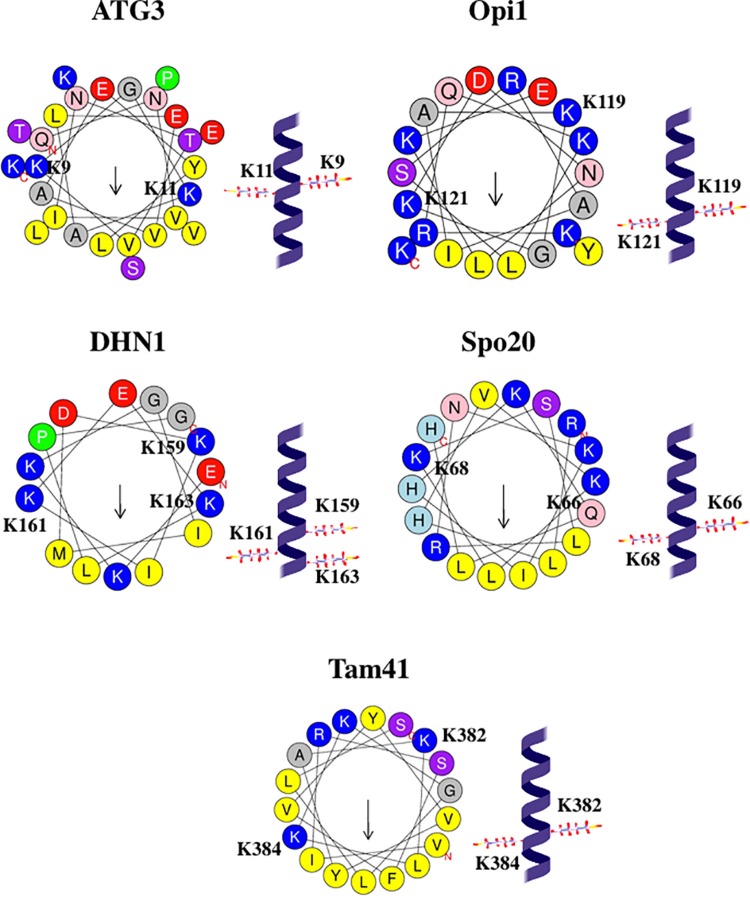
Configurations and helical wheel representations of some of the KxK motif-containing AHs belonging to the PA-binding proteins. Helical wheel projections were generated using Heliquest software (http://heliquest.ipmc.cnrs.fr; Gautier et al., 2008). Residues belonging to the KxK motifs are highlighted. Color coding for residues: yellow for hydrophobic, purple for Ser (S) and Thr (T), blue for Lys (K) and Arg (R), red for acidic, pink for Asn (N) and Gln (Q), gray for small residues (Ala, A and Gly, G), green for Pro (P), and light blue for His (H). The arrow in helical wheels corresponds to the hydrophobic moment. Species names and UniProt accession numbers: *Homo sapiens* ATG3, Q9NT62; *Saccharomyces cerevisiae* Opi1, P21957; *Zea mays* DHN1, P12950; *S. cerevisiae* Spo20, Q04359; *Schizosaccharomyces pombe* Tam41, O74339.

If KxK motif is present in the protein α-helix (each amino acid residue corresponds to a 100° turn in the helix), two Lys belonging to the KxK motif are on two opposite faces of the helix, see Figure 6. Hence, in the AH, KxK motif usually places two Lys at the polar/non-polar interface. Such a position of basic residues is typical for AHs that interact with membranes (Mishra and Palgunachari, 1996; Davidson et al., 1998; Polozov et al., 1998; Mozsolits et al., 2004), especially with membranes that contain acidic phospholipids (Polozov et al., 1998). Most Lys residues in the AH of α-synuclein belong to the KxK motifs, and, interestingly, Perrin et al. (2000) stress that such localization of Lys along the polar/non-polar interface might be important for α-synuclein interaction with PA-containing membranes. At first sight, such localization of two Lys, at the opposite sides of an α-helix, is not optimal for the interaction with the same lipid molecule. However, Hofbauer et al. (2018) underline that KRK motif in Opi1 interacts specifically with PA. Moreover, this motif forms a “three-finger grip” that tightly binds the PA phosphate headgroup but fails to accommodate the larger PS headgroup (Hofbauer et al., 2018). We suppose that, similarly, KxK motif in the AH of other proteins might form a “two-finger grip” that binds specifically the PA phosphate headgroup, although with somewhat lower affinity compared with KRK motif, if the residue between two Lys is not positively charged.

As we pointed out, the penetration of AHs into ca. 40% of monolayer thickness is optimal for generation of membrane curvature and driving membrane fission (Campelo et al., 2008; Zemel et al., 2008; Boucrot et al., 2012; Hanna et al., 2016). We hypothesize that the specific interaction of lipid cofactor with sequence motif ensures optimal depth of the insertion of AH into lipid bilayer. Insertion of AH approximately at the level of membrane hydrophilic-hydrophobic interface is optimal for generating membrane curvature (Li, 2018). The KxK motifs within PA-associated AH places two Lys close to this interface (see Figure 6) and, hence, is able to ensure shallow insertion of this AH into the membrane and generation of the high membrane curvature, required for driving membrane fission. Moreover, in few AHs of cardiolipin-binding proteins, such as AHs from MGS (Eriksson et al., 2009), AtPmtA (Danne et al., 2017b), Pex11p (Opaliński et al., 2011), the orientation of the (K/R)x_6_(F/Y) motif ensures the position of these two residues (basic Lys or Arg and aromatic Phe or Tyr) close to the hydrophilic-hydrophobic interface, so that basic residue is somewhat closer to the hydrophobic face of the helix, and aromatic residue is somewhat closer to the hydrophilic face of the helix, see Figure 4. We suggest that such orientation also ensures shallow insertion of the AH, that is optimal for driving fission.

Interestingly, α-synuclein that might use cardiolipin and, possibly, also PA as lipid cofactors in fission reaction (see above) contains, in its AH, putative cardiolipin-binding Kx_6_Y motif as well as five copies of putative PA-binding KxK motifs, see Tables 3, 5. Moreover, Atg3 that is not known to be involved in any membrane fission process, interacts with both cardiolipin and PA (Nath et al., 2014; Hervás et al., 2017), and contains, in its AH, putative cardiolipin-binding K^11^x_6_Y^18^ motif overlapping with the putative PA-binding K^9^xK^11^ motif.

We predict that mutations of residues belonging to the KxK motifs in AHs of PA-binding proteins as well as (K/R)x_6_(F/Y) motifs in AHs of cardiolipin-binding proteins will inhibit the binding of these proteins to the membranes containing these lipids. For proteins that possess an ability to generate membrane curvature or to mediate membrane fission, these processes will also be inhibitied in the mutants.

Interestingly, Lys in cardiolipin-binding and PA-binding AHs are much more frequent than Arg, see Tables 3, 5. Mutation of five Lys to Arg in Opi1 leads to the loss of the preference for PA-containing over PS-containing liposomes (Hofbauer et al., 2018). Due to its longer aliphatic side chain, Lys penetrate more deeply into lipid bilayer, and thus interaction of lipids with Lys might be more stable than interaction of lipids with Arg (Davidson et al., 1998). Furthermore, the increase in PA charge induced by Lys is higher than that induced by Arg (Kooijman et al., 2007). For these reasons, the presence of Lys in sequence motifs that recognize cardiolipin and PA is preferable to the presence of Arg.

Amphipathic helices of some of the fission-inducing proteins that use cardiolipin, PtdIns(4,5)P_2_ and PA as cofactors do not contain, correspondingly, (K/R)x_6_(F/Y) motif, (K/R/H)(K/R/H)xx(K/R/H) motif, and KxK motif. Other residues yet to be identified are therefore involved in the interaction with these lipids.

### Characteristics of Lipid Cofactors Involved in Membrane Fission Processes in Different Organelles

We noted that in many membrane fission processes mediated by AH-containing proteins in a particular organelle or bacterium, the most abundant lipids in this organelle (or bacterium) possessing high negative charge (more negative than −1) play a role of lipid cofactors. Moreover, fission-inducing proteins often contain in their AHs sequence motifs that recognize these lipids. The following highly charged lipids are over-represented in specific organelles and hence might play the role of lipid cofactors with various fission-inducing proteins: cardiolipin in bacteria, mitochondria (Musatov and Sedlak, 2017; Pagliuso et al., 2018) and peroxisomes (Wriessnegger et al., 2007); PtdIns3P and PtdIns(3,4)P_2_ in the endosomes; PtdIns4P in the Golgi apparatus; PtdIns5P in the nuclear envelope; PtdIns(4,5)P_2_ in the PM; PtdIns(3,5)P_2_ in endosomes, lysosomes and vacuoles (Takatori et al., 2016; Olivença et al., 2018; Dickson and Hille, 2019).

Knowing the primary sequence of the AH of a fission-inducing proteins allows to anticipate which lipid is used by this protein as a cofactor in fission reactions, and which organelle this protein functions in, see Table 6. For example, eukaryotic fission protein containing (K/R)x_6_(F/Y) motif in its AH might be expected to use cardiolipin as cofactor and, hence, to mediate fission of mitochondria or peroxisomes. On the other hand, a fission-inducing protein that does not contain (K/R)x_6_(F/Y) motif but contains a (K/R/H)(K/R/H)xx(K/R/H) motif in its AH might use PtdIns(4,5)P_2_ as cofactor and, hence, might be involved in endocytosis.

**TABLE 6:** Typical lipid cofactors that are used by fission-inducing proteins in different organelles and in bacterial cells, and putative sequence motifs within amphipathic helices of these proteins that specifically bind their lipid cofactors.

**Organelles**	**Lipid cofactors**	**Charge**	**Motif**	**Examples**
*Eukaryotic cell*				
Mitochondrion	Cardiolipin	−2	(K/R)x_6_(F/Y)	α-synuclein (Nakamura et al., 2008, 2011)
Peroxisome	Cardiolipin	−2	(K/R)x_6_(F/Y)	Pex11 (Opaliński et al., 2011; Su et al., 2018)
Endosome, vacuole, lysosome	PtdIns(3,5)P_2_	−4	–	Atg18 (Gopaldass et al., 2017)
Plasma membrane	PtdIns(4,5)P_2_	−4	(K/R/H)(K/R/H)xx(K/R/H)	Epsin (Boucrot et al., 2012; Lai et al., 2012) Endophilin A1 (Yoon et al., 2012)
*Bacterial cell*				
	Cardiolipin	−2	(K/R)x_6_(F/Y)	AtPmtA (Danne et al., 2017b) MGS (Eriksson et al., 2009; Ariöz et al., 2013)

As pointed out above, we hypothesize that the most abundant organelle-specific lipid possessing high negative charge might play a role of a lipid cofactor in membrane fission processes.

We have already mentioned AH-containing proteins (such as α-synuclein, Pex11, PmtA, MGS, DGS, epsin, endophilin A1) that mediate fission in different organelles and follow this rule, see Table 6. Most probably, a similar pattern also applies to fission-inducing proteins in general. For example, cardiolipin is a putative lipid cofactor for FisB that mediates fission during sporulation in a bacterium *B. subtilis* (Doan et al., 2013). FisB is not known to contain any AH. Moreover, cardiolipin activates the mechanoenzyme Drp1 that plays an important role in mitochondrial fission (Francy et al., 2017). Besides being cofactor for fission mediated by AH-containing proteins such as epsin (Kweon et al., 2006; Boucrot et al., 2012; Lai et al., 2012) and endophilin A1 (Yoon et al., 2012), PtdIns(4,5)P_2_ is a putative cofactor for the mechanoenzyme dynamin (Jost et al., 1998) that is responsible for endocytosis, i.e., fission at PM. PtdIns4P, whose charge is between −2 and −3 at physiological pH (van Paridon et al., 1986), was suggested to play a direct role in membrane fission of Golgi membranes mediated by CtBP1-S/BARS (Yang et al., 2008; Valente et al., 2013). Moreover, besides being cofactor for vacuole fission mediated by AH-containing fission-inducing protein Atg18 (Gopaldass et al., 2017), PtdIns(3,5)P_2_ plays an important role in various fission processes in vacuoles and endo-lysosomes (Dove et al., 2009; Miner et al., 2019; and references therein). We are not aware of any role that PtdIns5P plays in any fission process. However, this phenomenon might be expected in an organelle such as the nuclear envelope, in which the content of this lipid is high, compared with other highly charged lipids. This type of reasoning can be applied to other phosphoinositides enriched in different organelles.

## Oligomerization of Amphipathic Helix-Containing Fission-Inducing Proteins

An oligomeric structure is required for membrane fission and membrane curvature generation mediated by various AH-containing fission-inducing proteins. For example, Arf1 (Beck et al., 2008, 2011), Sar1 (Hariri et al., 2014), epsin 1 (Lai et al., 2012), endophilin A1 (Gallop et al., 2006; Mim et al., 2012; Yoon et al., 2012; Capraro et al., 2013), endophilin A2 (Gortat et al., 2012), *Drosophila* amphiphysin (Peter et al., 2004; Yoon et al., 2012), ANKHD1 (Kitamata et al., 2019), GDAP1 (Huber et al., 2016) form dimers. Endophilin B1 (Rostovtseva et al., 2009), PICK1 (Karlsen et al., 2015) and influenza virus M2 (Rossman et al., 2010) form tetramers (dimers of dimers). In some cases, dimers may direct the formation of higher order oligomers, such as, for example, in the case of epsin 1 (Yoon et al., 2010; Lai et al., 2012) and endophilin A1 (Gallop et al., 2006; Yoon et al., 2012). Moreover, dimers of Sar1 (Hariri et al., 2014), epsin 1 (Lai et al., 2012), and endophilin A1 (Mim et al., 2012; Capraro et al., 2013) were reported to form ordered lattices. Human Pex11B (Yoshida et al., 2015), fungal Pex11p (Su et al., 2018), Snf7 (Saksena et al., 2009), caveolin-1 (Fernandez et al., 2002), and α-synuclein (Nakamura et al., 2011) form oligomers. The protein mutations that disrupt their oligomerization inhibit the ability of Arf1 (Beck et al., 2008, 2011), epsin 1 (Yoon et al., 2010), and endophilin A2 (Gortat et al., 2012) to deform or vesiculate membranes.

Sometimes, lipid cofactors required for membrane fission events promote oligomerization of AH-containing fission-inducing proteins. Thus, PtdIns(3,4)P_2_ induces Atg18 oligomerization (Gopaldass et al., 2017), whereas cholesterol prevents disruption of influenza virus M2 tetramers (Ekanayake et al., 2016) and shifts these tetramers toward a more compact conformation (Herneisen et al., 2017). In general, lipid cofactors such as PtdIns(4,5)P_2_ for endophilin A1 and *Drosophila* amphiphysin (Yoon et al., 2012), can promote membrane penetration of the AHs and thus induce protein oligomerization upon membrane binding.

Formation of the oligomers of some AH-containing fission-inducing proteins, such as epsin 1 (Yoon et al., 2010), endophilin A1 (Mim et al., 2012), peroxins Pex11B (Yoshida et al., 2015) and Pex11p (Su et al., 2018), influenza virus M2 (Ekanayake et al., 2016), is driven, at least partially, by direct interaction of their AHs. Membrane curvature generated by the insertion of AH of fission-inducing protein into the membrane is amplified upon protein oligomerization due to the concerted penetration of multiple AHs, ultimately causing membrane tubulation or fission (Campelo et al., 2008; Yoon et al., 2010; Lai et al., 2012; Hariri et al., 2014; Pan et al., 2019).

Interestingly, oligomerization of endophilin B1 is induced by chaperone activity of the apoptosis regulator Bax (Rostovtseva et al., 2009). Bax does not stably associate with endophilin B1 oligomers. Despite of this, even when the concentration of Bax is 10 times lower than the concentration of endophilin B1, Bax induces endophilin B1 oligomerization, a structure required for membrane fission. As result, endophilin B1 with Bax induces massive vesiculation of liposomes, whereas endophilin B1 alone and Bax alone do not have such an effect (Rostovtseva et al., 2009). Interestingly, in normal cells, endophilin B1 and Bax do not interact. However, during apoptosis they bind transiently and, due to the membrane rearrangements mediated by oligomeric endophilin B1, they promote release of cytochrome C from mitochondria (Rostovtseva et al., 2009).

## Diseases Associated With Mutations in the Amphipathic Helices of Fission-Inducing Proteins

The role that AHs play in the function of fission-inducing proteins is emphasized by the fact that few naturally occurring mutations of residues belonging to the AHs are associated with human diseases. Some of these mutants are defective in inducing membrane curvature, whereas other mutants generate membrane curvature more vigorously than wt proteins. Both kinds of mutations might contribute to the development of diseases.

Amphiphysin 2 (also known as BIN1) contains an AH (Nicot et al., 2007; Böhm et al., 2014; Hohendahl et al., 2016) and induces membrane vesiculation (Wu and Baumgart, 2014). Deletion mutation ΔLys21 and substitution mutations Arg24Cys and Lys35Asn (Nicot et al., 2007; Böhm et al., 2014) within the AH Val18-Ala36 (Hohendahl et al., 2016) of amphiphysin 2 cause CNM. Unlike wt amphiphysin 2, none of these three mutants induce membrane tubulation (Nicot et al., 2007; Böhm et al., 2014). Nicot et al. (2007) and Hohendahl et al. (2016) underlined that these mutations change the charge of the hydrophilic face of the AH, and such change might disrupt the interaction with negatively charged lipids of the membrane and, in turn, may lead to a defect in the ability to generate membrane curvature.

Expression of GDAP1 induces fission of peroxisomes and mitochondria, and this fission is critically dependent on the AH Val292-Phe309 (Huber et al., 2016). The Asn297Lys mutation into the AH is associated with Charcot-Marie-Tooth disease (Moroni et al., 2009).

α-Synuclein induces mitochondrial fragmentation (Kamp et al., 2010; Nakamura et al., 2011; O’Donnell et al., 2014; Martinez et al., 2018) possibly explained by its role in membrane fission (Varkey et al., 2010; Nakamura et al., 2011; Fakhree et al., 2019). α-Synuclein contains a long AH that forms on membrane binding (George et al., 1995; Davidson et al., 1998; Drin and Antonny, 2010; Braun et al., 2017), and the insertion of this AH into membranes contributes to membrane curvature generation (Braun et al., 2014, 2017; Fakhree et al., 2019). The role of α-synuclein in mitochondrial fission was suggested to be related to the pathogenesis of Parkinson’s disease (Nakamura et al., 2011; Pozo Devoto and Falzone, 2017). Indeed, a few naturally occurring mutations of residues belonging to the α-synuclein AH (Pozo Devoto et al., 2017) are associated with Parkinson’s disease: Ala30Pro (Krüger et al., 1998), Glu46Lys (Zarranz et al., 2004), His50Gln (Appel-Cresswell et al., 2013), Gly51Asp (Kiely et al., 2013; Lesage et al., 2013), Ala53Thr (Polymeropoulos et al., 1997), and Ala53Glu (Pasanen et al., 2014). Overexpression of the Ala53Thr α-synuclein mutant led to more significant reductions of mitochondrial size than the overexpression of wt α-synuclein (Pozo Devoto et al., 2017). Uncontrolled mitochondrial fragmentation might contribute to the development of Parkinson’s disease (Varkey et al., 2010; Panchal and Tiwari, 2019). Hence, the role of Ala53Thr mutation in the development of Parkinson’s disease might be, at least partially, explained by the extensive mitochondrial fragmentation mediated by this mutant involving residue belonging to the AH.

In the future, the discovery of new naturally occurring mutations of residues within the AHs of fission-inducing proteins will facilitate the ultimate definition of the role that the interaction of AHs with the membranes play in the pathogenesis of the related diseases.

## Conclusion

In this review, we have considered fission-inducing proteins that contain AHs. Other fission-inducing proteins are known (Campelo and Malhotra, 2012; Frolov et al., 2015; Renard et al., 2015) indicating that the molecular events finally causing a bilayer to divide may follow different strategies. This would parallel the case of membrane fusion, where the structures of fusion-inducing proteins belonging to different classes are very different from each other and mechanisms of membrane rearrangements mediated by these proteins are also dissimilar (Kielian, 2014; Podbilewicz, 2014). The aspects that remain to be elucidated in both membrane fusion and fission are indeed the very last steps of these processes. While it is now relatively clear how the hemifission (or hemifusion) intermediate is obtained by the fission- or fusion-inducing proteins (see above and Chernomordik and Kozlov, 2008; Podbilewicz, 2014), the disruption of this structure and formation of the two bilayers (or their fusion) is an energy-demanding event not yet fully clarified. Protein rearrangements, lipid modifications or synthesis, GTP/GDP can all potentially contribute to this energy requirement. How they function, or if other molecules or sources of energy intervene, is still matter of debate. These are fascinating aspects in the membrane dynamics field that we are confident will be solved in the near future thanks to the continuously improved knowledge of the systems, together with the new morphological and instrumental tools being developed.

## Author Contributions

MZ, AL, DC, and CV wrote the manuscript. AF and CV made the figures. MZ and AF made the tables.

## Conflict of Interest

The authors declare that the research was conducted in the absence of any commercial or financial relationships that could be construed as a potential conflict of interest.

## Abbreviations

AH: amphipathic helix
ANKHD1: ankyrin repeat and KH domain-containing protein 1
Arf: ADP ribosylation factor
ArfGAP1: ADPribosylation factor GTPase-activating protein 1
Atg: autophagy-related protein
ATP: adenosine triphosphate
AtPmtA: Agrobacterium tumefaciens phospholipid N-methyltransferase
BAR: Bin/Amphiphysin/Rvs
BDLP1: bacterial dynaminlike protein 1
Bif-1: Bax-interacting factor 1
BIN1: bridging integrator-1
CALM: Clathrin Assembly Lymphoid-Myeloid leukemia protein
CCT: cytidine triphosphate:phosphocholine cytidylyltransferase
CCVs: clathrin-coated vesicles
CdvC: Cell division protein C
CL: cardiolipin
CME: clathrin-mediated endocytosis
CNM: centronuclear myopathy
COP: coat protein
CtBP1-S/BARS: C-terminal binding protein 1 shorter isoform/brefeldin A ADP-ribosylated substrate
DGS: diglucosyldiacylglycerol synthase
DHN1: dehydrin 1
Dnm2: dynamin2
DRP: dynamin-related protein
EcMurG: Escherichia coli MurG
EHD1: Eps15-homology domain-containing protein 1
ENTH/ANTH: Epsin N-Terminal Homology/AP180 N-Terminal Homology
EPR: electron paramagnetic resonance
ER: endoplasmic reticulum
ESCRT: endosomal sorting complexes required for transport
F-BAR: FCH-BAR
FEME: fast endophilin-mediated endocytosis
FisB: fission protein B
GDAP1: Ganglioside-induced Differentiation Associated Protein 1
GDP: guanosine diphosphate
GMAP-210: Golgi Microtubule-Associated Protein 210
GTP: guanosine triphosphate
GUVs: giant unilamellar vesicles
I-BAR: Inverse BAR
IMM: inner mitochondrial membrane
INF2: inverted formin-2
LPAAT: lysophosphatidic acid acyltransferase
MFF: mitochondrial fission factor
MGS: monoglucosyldiacylglycerol synthase
MiD49 and MiD51: mitochondrial dynamics proteins 49 and 51
MinD: septum site-determining protein MinD
MMPE: monomethyl-phosphatidylethanolamine
MurG: N-acetylmuramyl-(pentapeptide) pyrophosphoryl-undecaprenol N-acetylglucosamine transferase
N-BAR: N-terminal amphipathic helixcontaining BAR
OMM: outer mitochondrial membrane
Opi1: overproduction of inositol 1
PA: phosphatidic acid
PBP: phosphatidylinositol bisphosphate
PC: phosphatidylcholine
Pcyt1a: cytidine triphosphate:phosphocholine cytidylyltransferase alpha
Pex11: peroxisomal membrane protein 11
PG: phosphatidylglycerol
PICK1: protein interacting with C kinase 1
PM: plasma membrane
PmtA: phospholipid N-methyltransferase
POPG: 1-palmitoyl-2-oleoyl-sn-glycero-3-phosphatidylglycerol
PROPPIN: β-propellers that bind polyphosphoinositides
PS: phosphatidylserine
PtdIns: phosphatidylinositol
PtdIns3P: phosphatidylinositol 3-phosphate
PtdIns4P: phosphatidylinositol 4-phosphate
PtdIns5P: phosphatidylinositol 5-phosphate
PtdIns(3,4)P2: phosphatidylinositol 3,4-bisphosphate
PtdIns(3,5)P2: phosphatidylinositol 3,5-bisphosphate
PtdIns(3,4,5)P2: phosphatidylinositol 3,4,5-trisphosphate
PtdIns(4,5)P2: phosphatidylinositol 4,5-bisphosphate
Sar: secretion-associated Ras-related protein
SH3YL1: SRC homology 3 domain containing Ysc84-like 1
SNCA: α-synuclein
Snf7: sucrose non-fermenting protein 7
Spo20: sporulationspecific protein 20
STIM2: stromal interaction molecule 2
Tam41: translocator assembly and maintenance protein 41
VHS: Vps27, Hrs and STAM
VP40: viral protein 40
Vps4: Vacuolar protein sorting-associated protein 4A
wt: wild type

## References

[B1] Adachi Y. Itoh K. Yamada T. Cerveny K. L. Suzuki T. L. Macdonald P. (2016). Coincident phosphatidic acid interaction restrains Drp1 in mitochondrial division. *Mol. Cell* 63 1034–1043. 10.1016/j.molcel.2016.08.013 27635761 PMC5028122

[B2] Adolf F. Herrmann A. Hellwig A. Beck R. Brügger B. Wieland F. T. (2013). Scission of COPI and COPII vesicles is independent of GTP hydrolysis. *Traffic* 14 922–932. 10.1111/tra.12084 23691917

[B3] Adu-Gyamfi E. Johnson K. A. Fraser M. E. Scott J. L. Soni S. P. Jones K. R. (2015). Host cell plasma membrane phosphatidylserine regulates the assembly and budding of Ebola virus. *J. Virol.* 89 9440–9453. 10.1128/jvi.01087-15 26136573 PMC4542376

[B4] Agrawal A. Ramachandran R. (2019). Exploring the links between lipid geometry and mitochondrial fission: emerging concepts. *Mitochondrion.* 10.1016/j.mito.2019.07.010 [Epub ahead of print] 31351921

[B5] Ahmed I. Akram Z. Iqbal H. M. N. Munn A. L. (2019). The regulation of Endosomal Sorting Complex Required for Transport and accessory proteins in multivesicular body sorting and enveloped viral budding - an overview. *Int. J. Biol. Macromol.* 127 1–11. 10.1016/j.ijbiomac.2019.01.015 30615963

[B6] Ahmed S. Bu W. Lee R. T. C. Maurer-Stroh S. Goh W. I. (2010). F-BAR domain proteins: families and function. *Commun. Integr. Biol.* 3 116–121. 10.4161/cib.3.2.10808 20585502 PMC2889966

[B7] Albesa-Jové D. Giganti D. Jackson M. Alzari P. M. Guerin M. E. (2014). Structure–function relationships of membrane-associated GT-B glycosyltransferases. *Glycobiology* 24 108–124. 10.1093/glycob/cwt101 24253765 PMC3907083

[B8] Ambroso M. R. Hegde B. G. Langen R. (2014). Endophilin A1 induces different membrane shapes using a conformational switch that is regulated by phosphorylation. *Proc. Natl. Acad. Sci. U.S.A.* 111 6982–6987. 10.1073/pnas.1402233111 24778241 PMC4024918

[B9] Antonny B. Beraud-Dufour S. Chardin P. Chabre M. (1997). N-terminal hydrophobic residues of the G-protein ADP-ribosylation factor-1 insert into membrane phospholipids upon GDP to GTP exchange. *Biochemistry* 36 4675–4684. 10.1021/bi962252b 9109679

[B10] Antonny B. Burd C. De Camilli P. Chen E. Daumke O. Faelber K. (2016). Membrane fission by dynamin: what we know and what we need to know. *EMBO J.* 35 2270–2284. 27670760 10.15252/embj.201694613 PMC5090216

[B11] Appel-Cresswell S. Vilarino-Guell C. Encarnacion M. Sherman H. Yu I. Shah B. (2013). α-synuclein p.H50Q, a novel pathogenic mutation for Parkinson’s disease. *Mov. Disord.* 28 811–813. 10.1002/mds.25421 23457019

[B12] Ariotti N. Rae J. Leneva N. Ferguson C. Loo D. Okano S. (2015). Molecular characterization of caveolin-induced membrane curvature. *J. Biol. Chem.* 290 24875–24890. 10.1074/jbc.m115.644336 26304117 PMC4598997

[B13] Ariöz C. Gotzke H. Lindholm L. Eriksson J. Edwards K. Daley D. O. (2014). Heterologous overexpression of a monotopic glucosyltransferase (MGS) induces fatty acid remodeling in *Escherichia coli* membranes. *Biochim. Biophys. Acta* 1838 1862–1870. 10.1016/j.bbamem.2014.04.001 24726609

[B14] Ariöz C. Ye W. Bakali A. Ge C. Liebau J. Götzke H. (2013). Anionic lipid binding to the foreign protein MGS provides a tight coupling between phospholipid synthesis and protein overexpression in *Escherichia coli*. *Biochemistry* 52 5533–5544. 10.1021/bi400616n 23869703

[B15] Arnarez C. Marrink S. J. Periole X. (2016). Molecular mechanism of cardiolipin-mediated assembly of respiratory chain supercomplexes. *Chem. Sci.* 7 4435–4443. 10.1039/c5sc04664e 30155091 PMC6014297

[B16] Bacia K. Futai E. Prinz S. Meister A. Daum S. Glatte D. (2011). Multibudded tubules formed by COPII on artificial liposomes. *Sci. Rep.* 1:17. 10.1038/srep00017 PMC3216505 22355536

[B17] Bailer S. M. (2017). Venture from the interior-herpesvirus pUL31 escorts capsids from nucleoplasmic replication compartments to sites of primary envelopment at the inner nuclear membrane. *Cells* 6:E46. 10.3390/cells6040046 PMC5755504 29186822

[B18] Barneda D. Planas-Iglesias J. Gaspar M. L. Mohammadyani D. Prasannan S. Dormann D. (2015). The brown adipocyte protein CIDEA promotes lipid droplet fusion via a phosphatidic acid-binding amphipathic helix. *eLife* 4:e07485. 10.7554/eLife.07485 PMC4755750 26609809

[B19] Bashkirov P. V. Akimov S. A. Evseev A. I. Schmid S. L. Zimmerberg J. Frolov V. A. (2008). GTPase cycle of dynamin is coupled to membrane squeeze and release, leading to spontaneous fission. *Cell* 135 1276–1286. 10.1016/j.cell.2008.11.028 19084269 PMC2768395

[B20] Basu Ball W. Neff J. K. Gohil V. M. (2018). The role of nonbilayer phospholipids in mitochondrial structure and function. *FEBS Lett.* 592 1273–1290. 10.1002/1873-3468.12887 29067684 PMC5918238

[B21] Baumgart T. Capraro B. R. Zhu C. Das S. L. (2011). Thermodynamics and mechanics of membrane curvature generation and sensing by proteins and lipids. *Annu. Rev. Phys. Chem.* 62 483–506. 10.1146/annurev.physchem.012809.103450 21219150 PMC4205088

[B22] Beck R. Prinz S. Diestelkotter-Bachert P. Rohling S. Adolf F. Hoehner K. (2011). Coatomer and dimeric ADP ribosylation factor 1 promote distinct steps in membrane scission. *J. Cell Biol.* 194 765–777. 10.1083/jcb.201011027 21893600 PMC3171119

[B23] Beck R. Sun Z. Adolf F. Rutz C. Bassler J. Wild K. (2008). Membrane curvature induced by Arf1-GTP is essential for vesicle formation. *Proc. Natl. Acad. Sci. U.S.A.* 105 11731–11736. 10.1073/pnas.0805182105 18689681 PMC2575275

[B24] Bhardwaj R. Müller H. M. Nickel W. Seedorf M. (2013). Oligomerization and Ca^2 +^/calmodulin control binding of the ER Ca^2 +^-sensors STIM1 and STIM2 to plasma membrane lipids. *Biosci. Rep.* 33:e00077. 10.1042/BSR20130089 PMC3814058 24044355

[B25] Bhatia V. K. Madsen K. L. Bolinger P. Y. Kunding A. Hedegård P. Gether U. (2009). Amphipathic motifs in BAR domains are essential for membrane curvature sensing. *EMBO J.* 28 3303–3314. 10.1038/emboj.2009.261 19816406 PMC2776096

[B26] Bielli A. Haney C. J. Gabreski G. Watkins S. C. Bannykh S. I. Aridor M. (2005). Regulation of Sar1 NH_2_ terminus by GTP binding and hydrolysis promotes membrane deformation to control COPII vesicle fission. *J. Cell Biol.* 171 919–924. 10.1083/jcb.200509095 16344311 PMC2171319

[B27] Bigalke J. M. Heldwein E. E. (2015). Structural basis of membrane budding by the nuclear egress complex of herpesviruses. *EMBO J.* 34 2921–2936. 10.15252/embj.201592359 26511020 PMC4687684

[B28] Bitto N. J. Kaparakis-Liaskos M. (2017). The therapeutic benefit of bacterial membrane vesicles. *Int. J. Mol. Sci.* 18:E1287. 10.3390/ijms18061287 PMC5486109 28621731

[B29] Böhm J. Biancalana V. Malfatti E. Dondaine N. Koch C. Vasli N. (2014). Adult-onset autosomal dominant centronuclear myopathy due to BIN1 mutations. *Brain* 137 3160–3170. 10.1093/brain/awu272 25260562

[B30] Bohuszewicz O. Liu J. Low H. H. (2016). Membrane remodelling in bacteria. *J. Struct. Biol.* 196 3–14. 10.1016/j.jsb.2016.05.010 27265614 PMC6168058

[B31] Bonazzi M. Spanò S. Turacchio G. Cericola C. Valente C. Colanzi A. (2005). CtBP3/BARS drives membrane fission in dynamin-independent transport pathways. *Nat. Cell Biol.* 7 570–580. 10.1038/ncb1260 15880102

[B32] Bottanelli F. Kilian N. Ernst A. M. Rivera-Molina F. Schroeder L. K. Kromann E. B. (2017). A novel physiological role for ARF1 in the formation of bidirectional tubules from the Golgi. *Mol. Biol. Cell* 28 1676–1687. 10.1091/mbc.e16-12-0863 28428254 PMC5469610

[B33] Boucrot E. Ferreira A. P. A. Almeida-Souza L. Debard S. Vallis Y. Howard G. (2015). Endophilin marks and controls a clathrin-independent endocytic pathway. *Nature* 517 460–465. 10.1038/nature14067 25517094

[B34] Boucrot E. Pick A. Çamdere G. Liska N. Evergren E. McMahon H. T. (2012). Membrane fission is promoted by insertion of amphipathic helices and is restricted by crescent BAR domains. *Cell* 149 124–136. 10.1016/j.cell.2012.01.047 22464325 PMC3465558

[B35] Boyd K. J. Alder N. N. May E. R. (2018). Molecular dynamics analysis of cardiolipin and monolysocardiolipin on bilayer properties. *Biophys. J.* 114 2116–2127. 10.1016/j.bpj.2018.04.001 29742405 PMC5961467

[B36] Brady J. P. Claridge J. K. Smith P. G. Schnell J. R. (2015). A conserved amphipathic helix is required for membrane tubule formation by Yop1p. *Proc. Natl. Acad. Sci. U.S.A.* 112 E639–E648. 25646439 10.1073/pnas.1415882112 PMC4343081

[B37] Braun A. R. Lacy M. M. Ducas V. C. Rhoades E. Sachs J. N. (2014). α-Synuclein-induced membrane remodeling is driven by binding affinity, partition depth, and interleaflet order asymmetry. *J. Am. Chem. Soc.* 136 9962–9972. 10.1021/ja5016958 24960410 PMC4105054

[B38] Braun A. R. Lacy M. M. Ducas V. C. Rhoades E. Sachs J. N. (2017). α-Synuclein’s uniquely long amphipathic helix enhances its membrane binding and remodeling capacity. *J. Membr. Biol.* 250 183–193. 10.1007/s00232-017-9946-1 28239748 PMC5394797

[B39] Brooks A. Shoup D. Kustigian L. Puchalla J. Carr C. M. Rye H. S. (2015). Single particle fluorescence burst analysis of epsin induced membrane fission. *PLoS One* 10:e0119563. 10.1371/journal.pone.0119563 25799353 PMC4370887

[B40] Buchkovich N. J. Henne W. M. Tang S. Emr S. D. (2013). Essential N-terminal insertion motif anchors the ESCRT-III filament during MVB vesicle formation. *Dev. Cell* 27 201–214. 10.1016/j.devcel.2013.09.009 24139821

[B41] Campelo F. Malhotra V. (2012). Membrane fission: the biogenesis of transport carriers. *Annu. Rev. Biochem.* 81 407–427. 10.1146/annurev-biochem-051710-094912 22463692

[B42] Campelo F. McMahon H. T. Kozlov M. M. (2008). The hydrophobic insertion mechanism of membrane curvature generation by proteins. *Biophys. J.* 95 2325–2339. 10.1529/biophysj.108.133173 18515373 PMC2517036

[B43] Capraro B. R. Shi Z. Wu T. Chen Z. Dunn J. M. Rhoades E. (2013). Kinetics of endophilin N-BAR domain dimerization and membrane interactions. *J. Biol. Chem.* 288 12533–12543. 10.1074/jbc.m112.435511 23482561 PMC3642301

[B44] Carman P. J. Dominguez R. (2018). BAR domain proteins-a linkage between cellular membranes, signaling pathways, and the actin cytoskeleton. *Biophys. Rev.* 10 1587–1604. 10.1007/s12551-018-0467-7 30456600 PMC6297083

[B45] Carranza G. Angius F. Ilioaia O. Solgadi A. Miroux B. Arechaga I. (2017). Cardiolipin plays an essential role in the formation of intracellular membranes in *Escherichia coli*. *Biochim. Biophys. Acta Biomembr.* 1859 1124–1132. 10.1016/j.bbamem.2017.03.006 28284722

[B46] Caspi Y. Dekker C. (2018). Dividing the archaeal way: the ancient Cdv cell-division machinery. *Front. Microbiol.* 9:174 10.3389/fmicb.2018.00174 PMC5840170 29551994

[B47] Cevher-Keskin B. (2013). ARF1 and SAR1 GTPases in endomembrane trafficking in plants. *Int. J. Mol. Sci.* 14 18181–18199. 10.3390/ijms140918181 24013371 PMC3794775

[B48] Chen Z. Zhu C. Kuo C. J. Robustelli J. Baumgart T. (2016). The N-terminal amphipathic helix of endophilin does not contribute to its molecular curvature generation capacity. *J. Am. Chem. Soc.* 138 14616–14622. 10.1021/jacs.6b06820 27755867 PMC5562367

[B49] Chernomordik L. V. Kozlov M. M. (2008). Mechanics of membrane fusion. *Nat. Struct. Mol. Biol.* 15 675–683. 10.1038/nsmb.1455 18596814 PMC2548310

[B50] Chiaruttini N. Roux A. (2017). Dynamic and elastic shape transitions in curved ESCRT-III filaments. *Curr. Opin. Cell Biol.* 47 126–135. 10.1016/j.ceb.2017.07.002 28728013

[B51] Chong S. S. Y. Taneva S. G. Lee J. M. C. Cornell R. B. (2014). The curvature sensitivity of a membrane-binding amphipathic helix can be modulated by the charge on a flanking region. *Biochemistry* 53 450–461. 10.1021/bi401457r 24397368

[B52] Chowdhury S. Otomo C. Leitner A. Ohashi K. Aebersold R. Lander G. C. (2018). Insights into autophagosome biogenesis from structural and biochemical analyses of the ATG2A-WIPI4 complex. *Proc. Natl. Acad. Sci. U.S.A.* 115 E9792–E9801. 30185561 10.1073/pnas.1811874115 PMC6196511

[B53] Chuartzman S. G. Nevo R. Shimoni E. Charuvi D. Kiss V. Ohad I. (2008). Thylakoid membrane remodeling during state transitions in Arabidopsis. *Plant Cell* 20 1029–1039. 10.1105/tpc.107.055830 18398051 PMC2390732

[B54] Colanzi A. Hidalgo Carcedo C. Persico A. Cericola C. Turacchio G. Bonazzi M. (2007). The Golgi mitotic checkpoint is controlled by BARS-dependent fission of the Golgi ribbon into separate stacks in G2. *EMBO J.* 26 2465–2476. 10.1038/sj.emboj.7601686 17431394 PMC1868899

[B55] Corda D. Colanzi A. Luini A. (2006). The multiple activities of CtBP/BARS proteins: the Golgi view. *Trends Cell Biol.* 16 167–173. 10.1016/j.tcb.2006.01.007 16483777

[B56] Corda D. Iurisci C. Berrie C. P. (2002). Biological activities and metabolism of the lysophosphoinositides and glycerophosphoinositols. *Biochim. Biophys. Acta* 1582 52–69. 10.1016/s1388-1981(02)00137-3 12069810

[B57] Cornell R. B. (2016). Membrane lipid compositional sensing by the inducible amphipathic helix of CCT. *Biochim. Biophys. Acta* 1861 847–861. 10.1016/j.bbalip.2015.12.022 26747646

[B58] Cornell R. B. Taneva S. G. (2006). Amphipathic helices as mediators of the membrane interaction of amphitropic proteins, and as modulators of bilayer physical properties. *Curr. Protein Pept. Sci.* 7 539–552. 10.2174/138920306779025675 17168787

[B59] Daly J. L. Cullen P. J. (2018). Endoplasmic reticulum-endosome contact sites: specialized interfaces for orchestrating endosomal tubule fission? *Biochemistry* 57 6738–6740. 10.1021/acs.biochem.8b01176 30500172

[B60] Danne L. Aktas M. Gleichenhagen J. Grund N. Wagner D. Schwalbe H. (2015). Membrane-binding mechanism of a bacterial phospholipid N-methyltransferase. *Mol. Microbiol.* 95 313–331. 10.1111/mmi.12870 25403021

[B61] Danne L. Aktas M. Grund N. Bentler T. Erdmann R. Narberhaus F. (2017a). Dissection of membrane-binding and -remodeling regions in two classes of bacterial phospholipid N-methyltransferases. *Biochim. Biophys. Acta Biomembr.* 1859 2279–2288. 10.1016/j.bbamem.2017.09.013 28912104

[B62] Danne L. Aktas M. Unger A. Linke W. A. Erdmann R. Narberhaus F. (2017b). Membrane remodeling by a bacterial phospholipid-methylating enzyme. *mBio* 8:e02082-16. 10.1128/mBio.02082-16 PMC5312082 28196959

[B63] Daumke O. Praefcke G. J. K. (2016). Invited review: mechanisms of GTP hydrolysis and conformational transitions in the dynamin superfamily. *Biopolymers* 105 580–593. 10.1002/bip.22855 27062152 PMC5084822

[B64] Davidson W. S. Jonas A. Clayton D. F. George J. M. (1998). Stabilization of α-synuclein secondary structure upon binding to synthetic membranes. *J. Biol. Chem.* 273 9443–9449. 10.1074/jbc.273.16.9443 9545270

[B65] De Craene J.-O. Ripp R. Lecompte O. Thompson J. D. Poch O. Friant S. (2012). Evolutionary analysis of the ENTH/ANTH/VHS protein superfamily reveals a coevolution between membrane trafficking and metabolism. *BMC Genomics* 13:297. 10.1186/1471-2164-13-297 22748146 PMC3473312

[B66] Del Vecchio K. Frick C. T. Gc J. B. Oda S.-I. Gerstman B. S. Saphire E. O. (2018). A cationic, C-terminal patch and structural rearrangements in Ebola virus matrix VP40 protein control its interactions with phosphatidylserine. *J. Biol. Chem.* 293 3335–3349. 10.1074/jbc.m117.816280 29348171 PMC5836117

[B67] Dickson E. J. Hille B. (2019). Understanding phosphoinositides: rare, dynamic, and essential membrane phospholipids. *Biochem. J.* 476 1–23. 10.1042/bcj20180022 30617162 PMC6342281

[B68] Doan T. Coleman J. Marquis K. A. Meeske A. J. Burton B. M. Karatekin E. (2013). FisB mediates membrane fission during sporulation in Bacillus subtilis. *Genes Dev.* 27 322–334. 10.1101/gad.209049.112 23388828 PMC3576517

[B69] Dodonova S. O. Aderhold P. Kopp J. Ganeva I. Röhling S. Hagen W. J. H. (2017). 9Å structure of the COPI coat reveals that the Arf1 GTPase occupies two contrasting molecular environments. *eLife* 6:e26691. 10.7554/eLife.26691 PMC5482573 28621666

[B70] Dove S. K. Dong K. Kobayashi T. Williams F. K. Michell R. H. (2009). Phosphatidylinositol 3,5-bisphosphate and Fab1p/PIKfyve underPPIn endo-lysosome function. *Biochem. J.* 419 1–13. 10.1042/bj20081950 19272020

[B71] Drin G. Antonny B. (2010). Amphipathic helices and membrane curvature. *FEBS Lett.* 584 1840–1847. 10.1016/j.febslet.2009.10.022 19837069

[B72] Ducas V. C. Rhoades E. (2012). Quantifying interactions of β-synuclein and γ-synuclein with model membranes. *J. Mol. Biol.* 423 528–539. 10.1016/j.jmb.2012.08.008 22922472 PMC3582368

[B73] Duncan A. L. Ruprecht J. J. Kunji E. R. S. Robinson A. J. (2018). Cardiolipin dynamics and binding to conserved residues in the mitochondrial ADP/ATP carrier. *Biochim. Biophys. Acta Biomembr.* 1860 1035–1045. 10.1016/j.bbamem.2018.01.017 29366674 PMC5988563

[B74] Duncan M. C. Payne G. S. (2003). ENTH/ANTH domains expand to the Golgi. *Trends Cell Biol.* 13 211–215. 10.1016/s0962-8924(03)00076-x 12742163

[B75] Edman M. Berg S. Storm P. Wikström M. Vikström S. Öhman A. (2003). Structural features of glycosyltransferases synthesizing major bilayer and nonbilayer-prone membrane lipids in *Acholeplasma laidlawii* and *Streptococcus pneumoniae*. *J. Biol. Chem.* 278 8420–8428. 10.1074/jbc.m211492200 12464611

[B76] Eisenberg D. Weiss R. M. Terwilliger T. C. (1982). The helical hydrophobic moment: a measure of the amphiphilicity of a helix. *Nature* 299 371–374. 10.1038/299371a0 7110359

[B77] Eisenberg D. Weiss R. M. Terwilliger T. C. (1984). The hydrophobic moment detects periodicity in protein hydrophobicity. *Proc. Natl. Acad. Sci. U.S.A.* 81 140–144. 10.1073/pnas.81.1.140 6582470 PMC344626

[B78] Ekanayake E. V. Fu R. Cross T. A. (2016). Structural influences: cholesterol, drug, and proton binding to full-length Influenza A M2 protein. *Biophys. J.* 110 1391–1399. 10.1016/j.bpj.2015.11.3529 27028648 PMC4816700

[B79] Elkins M. R. Sergeyev I. V. Hong M. (2018). Determining cholesterol binding to membrane proteins by cholesterol ^13^C labeling in yeast and dynamic nuclear polarization NMR. *J. Am. Chem. Soc.* 140 15437–15449. 10.1021/jacs.8b09658 30338997 PMC6361393

[B80] Elkins M. R. Williams J. K. Gelenter M. D. Dai P. Kwon B. Sergeyev I. V. (2017). Cholesterol-binding site of the influenza M2 protein in lipid bilayers from solid-state NMR. *Proc. Natl. Acad. Sci. U.S.A.* 114 12946–12951. 10.1073/pnas.1715127114 29158386 PMC5724280

[B81] Ellen A. F. Zolghadr B. Driessen A. M. J. Albers S.-V. (2010). Shaping the archaeal cell envelope. *Archaea* 2010:608243. 10.1155/2010/608243 PMC2910488 20671907

[B82] Eriksson H. M. Wessman P. Ge C. Edwards K. Wieslander Å. (2009). Massive formation of intracellular membrane vesicles in *Escherichia coli* by a monotopic membrane-bound lipid glycosyltransferase. *J. Biol. Chem.* 284 33904–33914. 10.1074/jbc.m109.021618 19767390 PMC2797161

[B83] Fakhree M. A. A. Engelbertink S. A. J. van Leijenhorst-Groener K. A. Blum C. Claessens M. M. A. E. (2019). Cooperation of helix insertion and lateral pressure to remodel membranes. *Biomacromolecules* 20 1217–1223. 10.1021/acs.biomac.8b01606 30653915 PMC6581421

[B84] Falanga A. Cantisani M. Pedone C. Galdiero S. (2009). Membrane fusion and fission: enveloped viruses. *Protein Pept. Lett.* 16 751–759. 10.2174/092986609788681760 19601904

[B85] Farsad K. Ringstad N. Takei K. Floyd S. R. Rose K. De Camilli P. (2001). Generation of high curvature membranes mediated by direct endophilin bilayer interactions. *J. Cell Biol.* 155 193–200. 10.1083/jcb.200107075 11604418 PMC2198845

[B86] Feng Y. He D. Yao Z. Klionsky D. J. (2014). The machinery of macroautophagy. *Cell Res.* 24 24–41. 10.1038/cr.2013.168 24366339 PMC3879710

[B87] Fernandes F. Loura L. M. S. Chichón F. J. Carrascosa J. L. Fedorov A. Prieto M. (2008). Role of helix 0 of the N-BAR domain in membrane curvature generation. *Biophys. J.* 94 3065–3073. 10.1529/biophysj.107.113118 18199667 PMC2275701

[B88] Fernandez I. Ying Y. Albanesi J. Anderson R. G. W. (2002). Mechanism of caveolin filament assembly. *Proc. Natl. Acad. Sci. U.S.A.* 99 11193–11198. 10.1073/pnas.172196599 12167674 PMC123232

[B89] Ferreira A. P. A. Boucrot E. (2018). Mechanisms of carrier formation during clathrin-independent endocytosis. *Trends Cell Biol.* 28 188–200. 10.1016/j.tcb.2017.11.004 29241687

[B90] Ford M. G. J. Mills I. G. Peter B. J. Vallis Y. Praefcke G. J. K. Evans P. R. (2002). Curvature of clathrin-coated pits driven by epsin. *Nature* 419 361–366. 10.1038/nature01020 12353027

[B91] Ford M. G. J. Chappie J. S. (2019). The structural biology of the dynamin-related proteins: new insights into a diverse, multitalented family. *Traffic* 20 717–740. 10.1111/tra.12676 31298797 PMC6876869

[B92] Francy C. A. Clinton R. W. Fröhlich C. Murphy C. Mears J. A. (2017). Cryo-EM studies of Drp1 reveal cardiolipin interactions that activate the helical oligomer. *Sci. Rep.* 7:10744. 10.1038/s41598-017-11008-3 PMC5587723 28878368

[B93] Frolov V. A. Escalada A. Akimov S. A. Shnyrova A. V. (2015). Geometry of membrane fission. *Chem. Phys. Lipids* 185 129–140. 10.1016/j.chemphyslip.2014.07.006 25062896

[B94] Gallop J. L. Jao C. C. Kent H. M. Butler P. J. G. Evans P. R. Langen R. (2006). Mechanism of endophilin N-BAR domain-mediated membrane curvature. *EMBO J.* 25 2898–2910. 10.1038/sj.emboj.7601174 16763559 PMC1500843

[B95] Gatta A. T. Carlton J. G. (2019). The ESCRT-machinery: closing holes and expanding roles. *Curr. Opin. Cell Biol.* 59 121–132. 10.1016/j.ceb.2019.04.005 31132588

[B96] Gautier R. Douguet D. Antonny B. Drin G. (2008). HELIQUEST: a web server to screen sequences with specific α-helical properties. *Bioinformatics* 24 2101–2102. 10.1093/bioinformatics/btn392 18662927

[B97] Ge C. Georgiev A. Öhman A. Wieslander Å. Kelly A. A. (2011). Tryptophan residues promote membrane association for a plant lipid glycosyltransferase involved in phosphate stress. *J. Biol. Chem.* 286 6669–6684. 10.1074/jbc.m110.138495 21156807 PMC3057776

[B98] Ge C. Gómez-Llobregat J. Skwark M. J. Ruysschaert J.-M. Wieslander Å. Lindén M. (2014). Membrane remodeling capacity of a vesicle-inducing glycosyltransferase. *FEBS J.* 281 3667–3684. 10.1111/febs.12889 24961908

[B99] Genet G. Boyé K. Mathivet T. Ola R. Zhang F. Dubrac A. (2019). Endophilin-A2 dependent VEGFR2 endocytosis promotes sprouting angiogenesis. *Nat. Commun.* 10:2350. 10.1038/s41467-019-10359-x PMC6538628 31138815

[B100] George J. M. Jin H. Woods W. S. Clayton D. F. (1995). Characterization of a novel protein regulated during the critical period for song learning in the zebra finch. *Neuron* 15 361–372. 10.1016/0896-6273(95)90040-3 7646890

[B101] Ghio S. Camilleri A. Caruana M. Ruf V. C. Schmidt F. Leonov A. (2019). Cardiolipin promotes pore-forming activity of α-synuclein oligomers in mitochondrial membranes. *ACS Chem. Neurosci.* 10 3815–3829. 10.1021/acschemneuro.9b00320 31356747

[B102] Gifford S. M. Meyer P. (2015). Enzyme function is regulated by its localization. *Comput. Biol. Chem.* 59(Pt B), 113–122. 10.1016/j.compbiolchem.2015.08.004 26278972

[B103] Giménez-Andrés M. Čopič A. Antonny B. (2018). The many faces of amphipathic helices. *Biomolecules* 8:45. 10.3390/biom8030045 29976879 PMC6164224

[B104] Gopaldass N. Fauvet B. Lashuel H. Roux A. Mayer A. (2017). Membrane scission driven by the PROPPIN Atg18. *EMBO J.* 36 3274–3291. 10.15252/embj.201796859 29030482 PMC5686546

[B105] Gortat A. San-Roman M. J. Vannier C. Schmidt A. A. (2012). Single point mutation in Bin/Amphiphysin/Rvs (BAR) sequence of endophilin impairs dimerization, membrane shaping, and Src homology 3 domain-mediated partnership. *J. Biol. Chem.* 287 4232–4247. 10.1074/jbc.m111.325837 22167186 PMC3281743

[B106] Graham T. R. Kozlov M. M. (2010). Interplay of proteins and lipids in generating membrane curvature. *Curr. Opin. Cell Biol.* 22 430–436. 10.1016/j.ceb.2010.05.002 20605711 PMC3770468

[B107] Ha S. Walker D. Shi Y. Walker S. (2000). The 1.9 Å crystal structure of Escherichia coli MurG, a membrane-associated glycosyltransferase involved in peptidoglycan biosynthesis. *Protein Sci.* 9 1045–1052. 10.1110/ps.9.6.1045 10892798 PMC2144650

[B108] Hagen C. Dent K. C. Zeev-Ben-Mordehai T. Grange M. Bosse J. B. Whittle C. (2015). Structural basis of vesicle formation at the inner nuclear membrane. *Cell* 163 1692–1701. 10.1016/j.cell.2015.11.029 26687357 PMC4701712

[B109] Hanna M. G. T. Mela I. Wang L. Henderson R. M. Chapman E. R. Edwardson J. M. (2016). Sar1 GTPase activity is regulated by membrane curvature. *J. Biol. Chem.* 291 1014–1027. 10.1074/jbc.m115.672287 26546679 PMC4714187

[B110] Hariri H. Bhattacharya N. Johnson K. Noble A. J. Stagg S. M. (2014). Insights into the mechanisms of membrane curvature and vesicle scission by the small GTPase Sar1 in the early secretory pathway. *J. Mol. Biol.* 426 3811–3826. 10.1016/j.jmb.2014.08.023 25193674 PMC4254083

[B111] Hasegawa J. Tokuda E. Tenno T. Tsujita K. Sawai H. Hiroaki H. (2011). SH3YL1 regulates dorsal ruffle formation by a novel phosphoinositide-binding domain. *J. Cell Biol.* 193 901–916. 10.1083/jcb.201012161 21624956 PMC3105542

[B112] Heo W. D. Inoue T. Park W. S. Kim M. L. Park B. O. Wandless T. J. (2006). PI(3,4,5)P_3_ and PI(4,5)P_2_ lipids target proteins with polybasic clusters to the plasma membrane. *Science* 314 1458–1461. 10.1126/science.1134389 17095657 PMC3579512

[B113] Herlo R. Lund V. K. Lycas M. D. Jansen A. M. Khelashvili G. Andersen R. C. (2018). An amphipathic helix directs cellular membrane curvature sensing and function of the BAR domain protein PICK1. *Cell Rep.* 23 2056–2069. 10.1016/j.celrep.2018.04.074 29768204

[B114] Herneisen A. L. Sahu I. D. McCarrick R. M. Feix J. B. Lorigan G. A. Howard K. P. (2017). A budding-defective M2 mutant exhibits reduced membrane interaction, insensitivity to cholesterol, and perturbed interdomain coupling. *Biochemistry* 56 5955–5963. 10.1021/acs.biochem.7b00924 29034683 PMC6112238

[B115] Hervás J. H. Landajuela A. Antón Z. Shnyrova A. V. Goñi F. M. Alonso A. (2017). Human ATG3 binding to lipid bilayers: role of lipid geometry, and electric charge. *Sci. Rep.* 7:15614. 10.1038/s41598-017-15057-6 PMC5688168 29142222

[B116] Hidalgo Carcedo C. Bonazzi M. Spanò S. Turacchio G. Colanzi A. Luini A. (2004). Mitotic Golgi partitioning is driven by the membrane-fissioning protein CtBP3/BARS. *Science* 305 93–96. 10.1126/science.1097775 15232108

[B117] Hofbauer H. F. Gecht M. Fischer S. C. Seybert A. Frangakis A. S. Stelzer E. H. K. (2018). The molecular recognition of phosphatidic acid by an amphipathic helix in Opi1. *J. Cell Biol.* 217 3109–3126. 10.1083/jcb.201802027 29941475 PMC6122994

[B118] Hohendahl A. Roux A. Galli V. (2016). Structural insights into the centronuclear myopathy-associated functions of BIN1 and dynamin 2. *J. Struct. Biol.* 196 37–47. 10.1016/j.jsb.2016.06.015 27343996 PMC5039012

[B119] Hohendahl A. Talledge N. Galli V. Shen P. S. Humbert F. De Camilli P. (2017). Structural inhibition of dynamin-mediated membrane fission by endophilin. *eLife* 6:e26856. 10.7554/eLife.26856 PMC5663480 28933693

[B120] Horchani H. de Saint-Jean M. Barelli H. Antonny B. (2014). Interaction of the Spo20 membrane-sensor motif with phosphatidic acid and other anionic lipids, and influence of the membrane environment. *PLoS One* 9:e113484. 10.1371/journal.pone.0113484 25426975 PMC4245137

[B121] Hoyer M. J. Chitwood P. J. Ebmeier C. C. Striepen J. F. Qi R. Z. Old W. M. (2018). A novel class of ER membrane proteins regulates ER-associated endosome fission. *Cell* 175 254 e14–265 e14. 30220460 10.1016/j.cell.2018.08.030 PMC6195207

[B122] Huber N. Bieniossek C. Wagner K. M. Elsässer H.-P. Suter U. Berger I. (2016). Glutathione-conjugating and membrane-remodeling activity of GDAP1 relies on amphipathic C-terminal domain. *Sci. Rep.* 6:36930. 10.1038/srep36930 PMC5107993 27841286

[B123] Irajizad E. Ramachandran R. Agrawal A. (2019). Geometric instability catalyzes mitochondrial fission. *Mol. Biol. Cell* 30 160–168. 10.1091/mbc.e18-01-0018 30379601 PMC6337907

[B124] Isas J. M. Ambroso M. R. Hegde P. B. Langen J. Langen R. (2015). Tubulation by amphiphysin requires concentration-dependent switching from wedging to scaffolding. *Structure* 23 873–881. 10.1016/j.str.2015.02.014 25865245 PMC4437528

[B125] Isermann P. Lammerding J. (2017). Consequences of a tight squeeze: nuclear envelope rupture and repair. *Nucleus* 8 268–274. 10.1080/19491034.2017.1292191 28287898 PMC5499899

[B126] Jao C. C. Hegde B. G. Chen J. Haworth I. S. Langen R. (2008). Structure of membrane-bound α-synuclein from site-directed spin labeling and computational refinement. *Proc. Natl. Acad. Sci. U.S.A.* 105 19666–19671. 10.1073/pnas.0807826105 19066219 PMC2605001

[B127] Jao C. C. Hegde B. G. Gallop J. L. Hegde P. B. McMahon H. T. Haworth I. S. (2010). Roles of amphipathic helices and the Bin/Amphiphysin/Rvs (BAR) domain of endophilin in membrane curvature generation. *J. Biol. Chem.* 285 20164–20170. 10.1074/jbc.m110.127811 20418375 PMC2888429

[B128] Jiao H. Yin Y. Liu Z. (2019). Structures of the mitochondrial CDP-DAG synthase Tam41 suggest a potential lipid substrate pathway from membrane to the active site. *Structure* 27 1258 e4–1269 e4. 31178220 10.1016/j.str.2019.04.017

[B129] Jimah J. R. Hinshaw J. E. (2019). Structural insights into the mechanism of dynamin superfamily proteins. *Trends Cell Biol.* 29 257–273. 10.1016/j.tcb.2018.11.003 30527453 PMC9623552

[B130] Jost M. Simpson F. Kavran J. M. Lemmon M. A. Schmid S. L. (1998). Phosphatidylinositol-4,5-bisphosphate is required for endocytic coated vesicle formation. *Curr. Biol.* 8 1399–1402. 9889104 10.1016/s0960-9822(98)00022-0

[B131] Kaksonen M. Roux A. (2018). Mechanisms of clathrin-mediated endocytosis. *Nat. Rev. Mol. Cell Biol.* 19 313–326. 29410531 10.1038/nrm.2017.132

[B132] Kamp F. Exner N. Lutz A. K. Wender N. Hegermann J. Brunner B. (2010). Inhibition of mitochondrial fusion by α-synuclein is rescued by PINK1, Parkin and DJ-1. *EMBO J.* 29 3571–3589. 10.1038/emboj.2010.223 20842103 PMC2964170

[B133] Kaneda M. van Oostende-Triplet C. Chebli Y. Testerink C. Bednarek S. Y. Geitmann A. (2019). Plant AP180 N-terminal homolog (ANTH) proteins are involved in clathrin-dependent endocytosis during pollen tube growth in *Arabidopsis thaliana*. *Plant Cell Physiol*. 60 1316–1330. 30796435 10.1093/pcp/pcz036

[B134] Karlsen M. L. Thorsen T. S. Johner N. Ammendrup-Johnsen I. Erlendsson S. Tian X. (2015). Structure of dimeric and tetrameric complexes of the BAR domain protein PICK1 determined by small-angle X-Ray scattering. *Structure* 23 1258–1270. 10.1016/j.str.2015.04.020 26073603 PMC4502314

[B135] Kassas N. Tanguy E. Thahouly T. Fouillen L. Heintz D. Chasserot-Golaz S. (2017). Comparative characterization of phosphatidic acid sensors and their localization during frustrated phagocytosis. *J. Biol. Chem.* 292 4266–4279. 10.1074/jbc.m116.742346 28115519 PMC5354483

[B136] Khattree N. Ritter L. M. Goldberg A. F. X. (2013). Membrane curvature generation by a C-terminal amphipathic helix in peripherin-2/rds, a tetraspanin required for photoreceptor sensory cilium morphogenesis. *J. Cell Sci.* 126 4659–4670. 10.1242/jcs.126888 23886945 PMC3795338

[B137] Kielian M. (2014). Mechanisms of virus membrane fusion proteins. *Annu. Rev. Virol.* 1 171–189. 10.1146/annurev-virology-031413-085521 26958720

[B138] Kiely A. P. Asi Y. T. Kara E. Limousin P. Ling H. Lewis P. (2013). α-Synucleinopathy associated with G51D *SNCA* mutation: a link between Parkinson’s disease and multiple system atrophy? *Acta Neuropathol.* 125 753–769. 10.1007/s00401-013-1096-7 23404372 PMC3681325

[B139] Kim S. S. Upshur M. A. Saotome K. Sahu I. D. McCarrick R. M. Feix J. B. (2015). Cholesterol-dependent conformational exchange of the C-terminal domain of the Influenza A M2 protein. *Biochemistry* 54 7157–7167. 10.1021/acs.biochem.5b01065 26569023 PMC4734095

[B140] Kirkham M. Nixon S. J. Howes M. T. Abi-Rached L. Wakeham D. E. Hanzal-Bayer M. (2008). Evolutionary analysis and molecular dissection of caveola biogenesis. *J. Cell Sci.* 121 2075–2086. 10.1242/jcs.024588 18505796

[B141] Kitamata M. Hanawa-Suetsugu K. Maruyama K. Suetsugu S. (2019). Membrane-deformation ability of ANKHD1 is involved in the early endosome enlargement. *iScience* 17 101–118. 10.1016/j.isci.2019.06.020 31255983 PMC6606961

[B142] Kjaerulff O. Brodin L. Jung A. (2011). The structure and function of endophilin proteins. *Cell Biochem. Biophys.* 60 137–154. 10.1007/s12013-010-9137-5 21184288

[B143] Klupp B. G. Hellberg T. Rönfeldt S. Franzke K. Fuchs W. Mettenleiter T. C. (2018). Function of the nonconserved N-Terminal domain of pseudorabies virus pUL31 in nuclear egress. *J. Virol.* 92:e00566-18. 10.1128/JVI.00566-18 PMC6052286 29793954

[B144] Knorr R. L. Lipowsky R. Dimova R. (2015). Autophagosome closure requires membrane scission. *Autophagy* 11 2134–2137. 10.1080/15548627.2015.1091552 26466816 PMC4824592

[B145] Knorr R. L. Mizushima N. Dimova R. (2017). Fusion and scission of membranes: ubiquitous topological transformations in cells. *Traffic* 18 758–761. 10.1111/tra.12509 28799689

[B146] Koag M. C. Fenton R. D. Wilkens S. Close T. J. (2003). The binding of maize DHN1 to lipid vesicles. Gain of structure and lipid specificity. *Plant Physiol.* 131 309–316. 10.1104/pp.011171 12529538 PMC166810

[B147] Koag M.-C. Wilkens S. Fenton R. D. Resnik J. Vo E. Close T. J. (2009). The K-segment of maize DHN1 mediates binding to anionic phospholipid vesicles and concomitant structural changes. *Plant Physiol.* 150 1503–1514. 10.1104/pp.109.136697 19439573 PMC2705017

[B148] Konno T. Ross O. A. Puschmann A. Dickson D. W. Wszolek Z. K. (2016). Autosomal dominant Parkinson’s disease caused by *SNCA* duplications. *Parkinson. Relat. Disord.* 22(Suppl. 1), S1–S6. 10.1016/j.parkreldis.2015.09.007 PMC4820832 26350119

[B149] Kooijman E. E. Chupin V. Fuller N. L. Kozlov M. M. de Kruijff B. Burger K. N. J. (2005). Spontaneous curvature of phosphatidic acid and lysophosphatidic acid. *Biochemistry* 44 2097–2102. 10.1021/bi0478502 15697235

[B150] Kooijman E. E. King K. E. Gangoda M. Gericke A. (2009). Ionization properties of phosphatidylinositol polyphosphates in mixed model membranes. *Biochemistry* 48 9360–9371. 10.1021/bi9008616 19725516

[B151] Kooijman E. E. Tieleman D. P. Testerink C. Munnik T. Rijkers D. T. S. Burger K. N. J. (2007). An electrostatic/hydrogen bond switch as the basis for the specific interaction of phosphatidic acid with proteins. *J. Biol. Chem.* 282 11356–11364. 10.1074/jbc.m609737200 17277311

[B152] Kotani T. Kirisako H. Koizumi M. Ohsumi Y. Nakatogawa H. (2018). The Atg2-Atg18 complex tethers pre-autophagosomal membranes to the endoplasmic reticulum for autophagosome formation. *Proc. Natl. Acad. Sci. U.S.A.* 115 10363–10368. 10.1073/pnas.1806727115 30254161 PMC6187169

[B153] Kozlov M. M. McMahon H. T. Chernomordik L. V. (2010). Protein-driven membrane stresses in fusion and fission. *Trends Biochem. Sci.* 35 699–706. 10.1016/j.tibs.2010.06.003 20638285 PMC3556487

[B154] Kozlovsky Y. Kozlov M. M. (2003). Membrane fission: model for intermediate structures. *Biophys. J.* 85 85–96. 10.1016/s0006-3495(03)74457-9 12829467 PMC1303068

[B155] Krauss M. Jia J.-Y. Roux A. Beck R. Wieland F. T. De Camilli P. (2008). Arf1-GTP-induced tubule formation suggests a function of Arf family proteins in curvature acquisition at sites of vesicle budding. *J. Biol. Chem.* 283 27717–27723. 10.1074/jbc.m804528200 18693248 PMC3762545

[B156] Krüger R. Kuhn W. Müller T. Woitalla D. Graeber M. Kösel S. (1998). Ala30Pro mutation in the gene encoding α-synuclein in Parkinson’s disease. *Nat. Genet.* 18 106–108. 9462735 10.1038/ng0298-106

[B157] Kunji E. R. S. Aleksandrova A. King M. S. Majd H. Ashton V. L. Cerson E. (2016). The transport mechanism of the mitochondrial ADP/ATP carrier. *Biochim. Biophys. Acta* 1863 2379–2393. 27001633 10.1016/j.bbamcr.2016.03.015

[B158] Kweon D.-H. Shin Y.-K. Shin J. Y. Lee J.-H. Lee J.-B. Seo J.-H. (2006). Membrane topology of helix 0 of the Epsin N-terminal homology domain. *Mol. Cells* 21 428–435. 16819307

[B159] Lai C.-L. Jao C. C. Lyman E. Gallop J. L. Peter B. J. McMahon H. T. (2012). Membrane binding and self-association of the epsin N-terminal homology domain. *J. Mol. Biol.* 423 800–817. 10.1016/j.jmb.2012.08.010 22922484 PMC3682188

[B160] Lauwers E. Goodchild R. Verstreken P. (2016). Membrane lipids in presynaptic function and disease. *Neuron* 90 11–25. 10.1016/j.neuron.2016.02.033 27054615

[B161] Lavedan C. (1998). The synuclein family. *Genome Res.* 8 871–880. 10.1101/gr.8.9.871 9750188

[B162] Lee E. Marcucci M. Daniell L. Pypaert M. Weisz O. A. Ochoa G.-C. (2002). Amphiphysin 2 (Bin1) and T-tubule biogenesis in muscle. *Science* 297 1193–1196. 10.1126/science.1071362 12183633

[B163] Lee M. C. S. Orci L. Hamamoto S. Futai E. Ravazzola M. Schekman R. (2005). Sar1p N-terminal helix initiates membrane curvature and completes the fission of a COPII vesicle. *Cell* 122 605–617. 10.1016/j.cell.2005.07.025 16122427

[B164] Legendre-Guillemin V. Wasiak S. Hussain N. K. Angers A. McPherson P. S. (2004). ENTH/ANTH proteins and clathrin-mediated membrane budding. *J. Cell Sci.* 117 9–18. 10.1242/jcs.00928 14657269

[B165] Lesage S. Anheim M. Letournel F. Bousset L. Honoré A. Rozas N. (2013). G51D α-synuclein mutation causes a novel parkinsonian-pyramidal syndrome. *Ann. Neurol.* 73 459–471. 10.1002/ana.23894 23526723

[B166] Li J. Wang X. Zhang T. Wang C. Huang Z. Luo X, et al. (2015). A review on phospholipids and their main applications in drug delivery systems. *Asian J. Pharmaceutic. Sci.* 10 81–98. 10.1016/j.ajps.2014.09.004

[B167] Li W.-W. Li J. Bao J.-K. (2012). Microautophagy: lesser-known self-eating. *Cell Mol. Life Sci.* 69 1125–1136. 10.1007/s00018-011-0865-5 22080117 PMC11114512

[B168] Li Z. -L. (2018). Molecular dynamics simulations of membrane deformation induced by amphiphilic helices of Epsin, Sar1p, and Arf1. *Chin. Phys. B* 27:038703 10.1088/1674-1056/27/3/038703

[B169] Liberali P. Kakkonen E. Turacchio G. Valente C. Spaar A. Perinetti G. (2008). The closure of Pak1-dependent macropinosomes requires the phosphorylation of CtBP1/BARS. *EMBO J.* 27 970–981. 10.1038/emboj.2008.59 18354494 PMC2323256

[B170] Lind J. Rämö T. Klement M. L. Bárány-Wallje E. Epand R. M. Epand R. F. (2007). High cationic charge and bilayer interface-binding helices in a regulatory lipid glycosyltransferase. *Biochemistry* 46 5664–5677. 10.1021/bi700042x 17444657

[B171] Loewen C. J. R. Gaspar M. L. Jesch S. A. Delon C. Ktistakis N. T. Henry S. A. (2004). Phospholipid metabolism regulated by a transcription factor sensing phosphatidic acid. *Science* 304 1644–1647. 10.1126/science.1096083 15192221

[B172] Loftus A. F. Hsieh V. L. Parthasarathy R. (2012). Modulation of membrane rigidity by the human vesicle trafficking proteins Sar1A and Sar1B. *Biochem. Biophys. Res. Commun.* 426 585–589. 10.1016/j.bbrc.2012.08.131 22974979

[B173] Loncle N. Agromayor M. Martin-Serrano J. Williams D. W. (2015). An ESCRT module is required for neuron pruning. *Sci. Rep.* 5:8461. 10.1038/srep08461 PMC4327575 25676218

[B174] Lorenz M. Vollmer B. Unsay J. D. Klupp B. G. García-Sáez A. J. Mettenleiter T. C. (2015). A single herpesvirus protein can mediate vesicle formation in the nuclear envelope. *J. Biol. Chem.* 290 6962–6974. 10.1074/jbc.m114.627521 25605719 PMC4358120

[B175] Löw C. Weininger U. Lee H. Schweimer K. Neundorf I. Beck-Sickinger A. G. (2008). Structure and dynamics of helix-0 of the N-BAR domain in lipid micelles and bilayers. *Biophys. J.* 95 4315–4323. 10.1529/biophysj.108.134155 18658220 PMC2567947

[B176] Lundmark R. Doherty G. J. Vallis Y. Peter B. J. McMahon H. T. (2008). Arf family GTP loading is activated by, and generates, positive membrane curvature. *Biochem J* 414 189–194. 10.1042/bj20081237 18597672 PMC2518064

[B177] Madsen J. J. Grime J. M. A. Rossman J. S. Voth G. A. (2018). Entropic forces drive clustering and spatial localization of influenza A M2 during viral budding. *Proc. Natl. Acad. Sci. U.S.A.* 115 E8595–E8603. 30150411 10.1073/pnas.1805443115 PMC6140502

[B178] Manifava M. Thuring J. W. J. F. Lim Z.-Y. Packman L. Holmes A. B. Ktistakis N. T. (2001). Differential binding of traffic-related proteins to phosphatidic acid- or phosphatidylinositol (4,5)- bisphosphate-coupled affinity reagents. *J. Biol. Chem.* 276 8987–8994. 10.1074/jbc.m010308200 11124268

[B179] Martens S. McMahon H. T. (2008). Mechanisms of membrane fusion: disparate players and common principles. *Nat. Rev. Mol. Cell Biol.* 9 543–556. 10.1038/nrm2417 18496517

[B180] Martin T. F. J. (2012). Role of PI(4,5)P_2_ in vesicle exocytosis and membrane fusion. *Subcell Biochem.* 59 111–130. 10.1007/978-94-007-3015-1_4 22374089 PMC3978774

[B181] Martinez J. H. Alaimo A. Gorojod R. M. Porte Alcon S. Fuentes F. Coluccio Leskow F. (2018). Drp-1 dependent mitochondrial fragmentation and protective autophagy in dopaminergic SH-SY5Y cells overexpressing α-synuclein. *Mol. Cell Neurosci.* 88 107–117. 10.1016/j.mcn.2018.01.004 29414102

[B182] Martyna A. Bahsoun B. Badham M. D. Srinivasan S. Howard M. J. Rossman J. S. (2017). Membrane remodeling by the M2 amphipathic helix drives influenza virus membrane scission. *Sci. Rep.* 7:44695. 10.1038/srep44695 PMC5357790 28317901

[B183] Masuda M. Takeda S. Sone M. Ohki T. Mori H. Kamioka Y. (2006). Endophilin BAR domain drives membrane curvature by two newly identified structure-based mechanisms. *EMBO J.* 25 2889–2897. 10.1038/sj.emboj.7601176 16763557 PMC1500852

[B184] McDargh Z. A. Deserno M. (2018). Dynamin’s helical geometry does not destabilize membranes during fission. *Traffic* 19 328–335. 10.1111/tra.12555 29437294

[B185] McLaughlin S. Wang J. Gambhir A. Murray D. (2002). PIP_2_ and proteins: interactions, organization, and information flow. *Annu. Rev. Biophys. Biomol. Struct.* 31 151–175. 10.1146/annurev.biophys.31.082901.134259 11988466

[B186] McMahon H. T. Boucrot E. (2015). Membrane curvature at a glance. *J. Cell Sci.* 128 1065–1070. 10.1242/jcs.114454 25774051 PMC4359918

[B187] McMahon H. T. Gallop J. L. (2005). Membrane curvature and mechanisms of dynamic cell membrane remodelling. *Nature* 438 590–596. 10.1038/nature04396 16319878

[B188] Meinecke M. Boucrot E. Camdere G. Hon W.-C. Mittal R. McMahon H. T. (2013). Cooperative recruitment of dynamin and BIN/Amphiphysin/Rvs (BAR) domain-containing proteins leads to GTP-dependent membrane scission. *J. Biol. Chem.* 288 6651–6661. 10.1074/jbc.m112.444869 23297414 PMC3585104

[B189] Melero A. Chiaruttini N. Karashima T. Riezman I. Funato K. Barlowe C. (2018). Lysophospholipids facilitate COPII vesicle formation. *Curr. Biol.* 28 1950.e6–1958 e6. 29887313 10.1016/j.cub.2018.04.076 PMC6013297

[B190] Mettlen M. Chen P.-H. Srinivasan S. Danuser G. Schmid S. L. (2018). Regulation of clathrin-mediated endocytosis. *Annu. Rev. Biochem.* 87 871–896. 29661000 10.1146/annurev-biochem-062917-012644 PMC6383209

[B191] Miller S. E. Mathiasen S. Bright N. A. Pierre F. Kelly B. T. Kladt N. (2015). CALM regulates clathrin-coated vesicle size and maturation by directly sensing and driving membrane curvature. *Dev. Cell* 33 163–175. 10.1016/j.devcel.2015.03.002 25898166 PMC4406947

[B192] Mim C. Cui H. Gawronski-Salerno J. A. Frost A. Lyman E. Voth G. A. (2012). Structural basis of membrane bending by the N-BAR protein endophilin. *Cell* 149 137–145. 10.1016/j.cell.2012.01.048 22464326 PMC3319357

[B193] Miner G. E. Sullivan K. D. Guo A. Jones B. C. Hurst L. R. Ellis E. C. (2019). Phosphatidylinositol 3,5-bisphosphate regulates the transition between *trans*-SNARE complex formation and vacuole membrane fusion. *Mol. Biol. Cell* 30 201–208. 10.1091/mbc.e18-08-0505 30427760 PMC6589561

[B194] Mishra V. K. Palgunachari M. N. (1996). Interaction of model class A_1_, class A_2_, and class Y amphipathic helical peptides with membranes. *Biochemistry* 35 11210–11220. 10.1021/bi960760f 8780526

[B195] Mizuno S. Sasai H. Kume A. Takahashi D. Satoh M. Kado S. (2017). Dioleoyl-phosphatidic acid selectively binds to α-synuclein and strongly induces its aggregation. *FEBS Lett.* 591 784–791. 10.1002/1873-3468.12592 28186641

[B196] Monroe N. Han H. Gonciarz M. D. Eckert D. M. Karren M. A. Whitby F. G. (2014). The oligomeric state of the active Vps4 AAA ATPase. *J. Mol. Biol.* 426 510–525. 10.1016/j.jmb.2013.09.043 24161953 PMC3919030

[B197] Moroni I. Morbin M. Milani M. Ciano C. Bugiani M. Pagliano E. (2009). Novel mutations in the *GDAP1* gene in patients affected with early-onset axonal Charcot-Marie-Tooth type 4A. *Neuromuscul. Disord.* 19 476–480. 10.1016/j.nmd.2009.04.014 19500985

[B198] Mozsolits H. Lee T.-H. Clayton A. H. Sawyer W. H. Aguilar M.-I. (2004). The membrane-binding properties of a class A amphipathic peptide. *Eur. Biophys. J.* 33 98–108. 10.1007/s00249-003-0332-9 12879312

[B199] Musatov A. Sedlák E. (2017). Role of cardiolipin in stability of integral membrane proteins. *Biochimie* 142 102–111. 10.1016/j.biochi.2017.08.013 28842204

[B200] Nakamura K. (2013). α-Synuclein and mitochondria: partners in crime? *Neurotherapeutics* 10 391–399. 10.1007/s13311-013-0182-9 23512373 PMC3701775

[B201] Nakamura K. Nemani V. M. Azarbal F. Skibinski G. Levy J. M. Egami K. (2011). Direct membrane association drives mitochondrial fission by the Parkinson disease-associated protein α-synuclein. *J. Biol. Chem.* 286 20710–20726. 10.1074/jbc.m110.213538 21489994 PMC3121472

[B202] Nakamura K. Nemani V. M. Wallender E. K. Kaehlcke K. Ott M. Edwards R. H. (2008). Optical reporters for the conformation of α-synuclein reveal a specific interaction with mitochondria. *J. Neurosci.* 28 12305–12317. 10.1523/jneurosci.3088-08.2008 19020024 PMC6671709

[B203] Nath S. Dancourt J. Shteyn V. Puente G. Fong W. M. Nag S. (2014). Lipidation of the LC3/GABARAP family of autophagy proteins relies on a membrane-curvature-sensing domain in Atg3. *Nat. Cell Biol.* 16 415–424. 10.1038/ncb2940 24747438 PMC4111135

[B204] Nicot A.-S. Toussaint A. Tosch V. Kretz C. Wallgren-Pettersson C. Iwarsson E. (2007). Mutations in amphiphysin 2 (*BIN*1) disrupt interaction with dynamin 2 and cause autosomal recessive centronuclear myopathy. *Nat. Genet.* 39 1134–1139. 10.1038/ng2086 17676042

[B205] Nishimura T. Morone N. Suetsugu S. (2018). Membrane re-modelling by BAR domain superfamily proteins via molecular and non-molecular factors. *Biochem. Soc. Trans.* 46 379–389. 10.1042/bst20170322 29540508

[B206] O’Donnell K. C. Lulla A. Stahl M. C. Wheat N. D. Bronstein J. M. Sagasti A. (2014). Axon degeneration and PGC-1α-mediated protection in a zebrafish model of α-synuclein toxicity. *Dis. Model. Mech.* 7 571–582. 10.1242/dmm.013185 24626988 PMC4007408

[B207] Olgiati S. Thomas A. Quadri M. Breedveld G. J. Graafland J. Eussen H. (2015). Early-onset parkinsonism caused by α-synuclein gene triplication: clinical and genetic findings in a novel family. *Parkinson. Relat. Disord.* 21 981–986. 10.1016/j.parkreldis.2015.06.005 26077166

[B208] Olivença D. V. Uliyakina I. Fonseca L. L. Amaral M. D. Voit E. O. Pinto F. R. (2018). A mathematical model of the phosphoinositide pathway. *Sci. Rep.* 8:3904. 10.1038/s41598-018-22226-8 PMC5834545 29500467

[B209] Opaliński Ł. Kiel J. A. K. W. Williams C. Veenhuis M. van der Klei I. J. (2011). Membrane curvature during peroxisome fission requires Pex11. *EMBO J.* 30 5–16. 10.1038/emboj.2010.299 21113128 PMC3020119

[B210] Osawa T. Alam J. M. Noda N. N. (2019). Membrane-binding domains in autophagy. *Chem. Phys. Lipids* 218 1–9. 10.1016/j.chemphyslip.2018.11.001 30414879

[B211] Osteryoung K. W. Pyke K. A. (2014). Division and dynamic morphology of plastids. *Annu. Rev. Plant Biol.* 65 443–472. 10.1146/annurev-arplant-050213-035748 24471836

[B212] Ouberai M. M. Wang J. Swann M. J. Galvagnion C. Guilliams T. Dobson C. M. (2013). α-Synuclein senses lipid packing defects and induces lateral expansion of lipids leading to membrane remodeling. *J. Biol. Chem.* 288 20883–20895. 10.1074/jbc.m113.478297 23740253 PMC3774359

[B213] Pagliuso A. Cossart P. Stavru F. (2018). The ever-growing complexity of the mitochondrial fission machinery. *Cell Mol. Life Sci.* 75 355–374. 10.1007/s00018-017-2603-0 28779209 PMC5765209

[B214] Pagliuso A. Valente C. Giordano L. L. Filograna A. Li G. Circolo D. (2016). Golgi membrane fission requires the CtBP1-S/BARS-induced activation of lysophosphatidic acid acyltransferase δ. *Nat. Commun.* 7:12148. 10.1038/ncomms12148 PMC4945875 27401954

[B215] Palomares-Jerez M. F. Nemesio H. Franquelim H. G. Castanho M. A. R. B. Villalaín J. (2013). N-terminal AH2 segment of protein NS4B from hepatitis C virus. Binding to and interaction with model biomembranes. *Biochim. Biophys. Acta* 1828 1938–1952. 10.1016/j.bbamem.2013.04.020 23639583

[B216] Pan J. Dalzini A. Song L. (2019). Cholesterol and phosphatidylethanolamine lipids exert opposite effects on membrane modulations caused by the M2 amphipathic helix. *Biochim. Biophys. Acta Biomembr.* 1861 201–209. 10.1016/j.bbamem.2018.07.013 30071193 PMC6260955

[B217] Pan L. Wu H. Shen C. Shi Y. Jin W. Xia J. (2007). Clustering and synaptic targeting of PICK1 requires direct interaction between the PDZ domain and lipid membranes. *EMBO J.* 26 4576–4587. 10.1038/sj.emboj.7601860 17914463 PMC2063473

[B218] Panchal K. Tiwari A. K. (2019). Mitochondrial dynamics, a key executioner in neurodegenerative diseases. *Mitochondrion* 47 151–173. 10.1016/j.mito.2018.11.002 30408594

[B219] Pannuzzo M. McDargh Z. A. Deserno M. (2018). The role of scaffold reshaping and disassembly in dynamin driven membrane fission. *eLife* 7:e39441. 10.7554/eLife.39441 PMC6355196 30561335

[B220] Park S.-Y. Yang J.-S. Li Z. Deng P. Zhu X. Young D. (2019). The late stage of COPI vesicle fission requires shorter forms of phosphatidic acid and diacylglycerol. *Nat. Commun.* 10:3409. 10.1038/s41467-019-11324-4 PMC6667475 31363100

[B221] Parton R. G. Hanzal-Bayer M. Hancock J. F. (2006). Biogenesis of caveolae: a structural model for caveolin-induced domain formation. *J. Cell Sci.* 119 787–796. 10.1242/jcs.02853 16495479

[B222] Pasanen P. Myllykangas L. Siitonen M. Raunio A. Kaakkola S. Lyytinen J. (2014). Novel α-synuclein mutation A53E associated with atypical multiple system atrophy and Parkinson’s disease-type pathology. *Neurobiol. Aging* 35:2180 e1-e5. 10.1016/j.neurobiolaging.2014.03.024 24746362

[B223] Perrin R. J. Woods W. S. Clayton D. F. George J. M. (2000). Interaction of human α-Synuclein and Parkinson’s disease variants with phospholipids. Structural analysis using site-directed mutagenesis. *J. Biol. Chem.* 275 34393–34398. 10.1074/jbc.m004851200 10952980

[B224] Peter B. J. Kent H. M. Mills I. G. Vallis Y. Butler P. J. G. Evans P. R. (2004). BAR domains as sensors of membrane curvature: the amphiphysin BAR structure. *Science* 303 495–499. 10.1126/science.1092586 14645856

[B225] Petersen J. Eriksson S. K. Harryson P. Pierog S. Colby T. Bartels D. (2012). The lysine-rich motif of intrinsically disordered stress protein CDeT11-24 from *Craterostigma plantagineum* is responsible for phosphatidic acid binding and protection of enzymes from damaging effects caused by desiccation. *J. Exp. Bot.* 63 4919–4929. 10.1093/jxb/ers173 22791833 PMC3428009

[B226] Piper R. C. Katzmann D. J. (2007). Biogenesis and function of multivesicular bodies. *Annu. Rev. Cell Dev. Biol.* 23 519–547. 10.1146/annurev.cellbio.23.090506.123319 17506697 PMC2911632

[B227] Planas-Iglesias J. Dwarakanath H. Mohammadyani D. Yanamala N. Kagan V. E. Klein-Seetharaman J. (2015). Cardiolipin interactions with proteins. *Biophys. J.* 109 1282–1294. 10.1016/j.bpj.2015.07.034 26300339 PMC4576322

[B228] Podbilewicz B. (2014). Virus and cell fusion mechanisms. *Annu. Rev. Cell Dev. Biol.* 30 111–139. 10.1146/annurev-cellbio-101512-122422 25000995

[B229] Polozov I. V. Polozova A. I. Mishra V. K. Anantharamaiah G. M. Segrest J. P. Epand R. M. (1998). Studies of kinetics and equilibrium membrane binding of class A and class L model amphipathic peptides. *Biochim. Biophys. Acta* 1368 343–354. 10.1016/s0005-2736(97)00210-1 9459611

[B230] Polymeropoulos M. H. Lavedan C. Leroy E. Ide S. E. Dehejia A. Dutra A. (1997). Mutation in the α-synuclein gene identified in families with Parkinson’s disease. *Science* 276 2045–2047. 10.1126/science.276.5321.2045 9197268

[B231] Pozo Devoto V. M. Dimopoulos N. Alloatti M. Pardi M. B. Saez T. M. Otero M. G. (2017). αSynuclein control of mitochondrial homeostasis in human-derived neurons is disrupted by mutations associated with Parkinson’s disease. *Sci. Rep.* 7:5042. 10.1038/s41598-017-05334-9 PMC5506004 28698628

[B232] Pozo Devoto V. M. Falzone T. L. (2017). Mitochondrial dynamics in Parkinson’s disease: a role for α-synuclein? *Dis. Model. Mech.* 10 1075–1087. 10.1242/dmm.026294 28883016 PMC5611962

[B233] Prokic I. Cowling B. S. Laporte J. (2014). Amphiphysin 2 (BIN1) in physiology and diseases. *J. Mol. Med.* 92 453–463. 10.1007/s00109-014-1138-1 24590001

[B234] Putta P. Rankenberg J. Korver R. A. van Wijk R. Munnik T. Testerink C. (2016). Phosphatidic acid binding proteins display differential binding as a function of membrane curvature stress and chemical properties. *Biochim. Biophys. Acta* 1858 2709–2716. 10.1016/j.bbamem.2016.07.014 27480805

[B235] Ramachandran R. (2018). Mitochondrial dynamics: the dynamin superfamily and execution by collusion. *Semin. Cell Dev. Biol.* 76 201–212. 10.1016/j.semcdb.2017.07.039 28754444 PMC5785577

[B236] Ramachandran R. Schmid S. L. (2018). The dynamin superfamily. *Curr. Biol.* 28 R411–R416. 10.1016/j.semcdb.2017.07.039 29689225

[B237] Randazzo P. A. (1997). Functional interaction of ADP-ribosylation factor 1 with phosphatidylinositol 4,5-bisphosphate. *J. Biol. Chem.* 272 7688–7692. 9065426

[B238] Renard H.-F. Johannes L. Morsomme P. (2018). Increasing diversity of biological membrane fission mechanisms. *Trends Cell Biol.* 28 274–286. 10.1016/j.tcb.2017.12.001 29307448

[B239] Renard H.-F. Simunovic M. Lemière J. Boucrot E. Garcia-Castillo M. D. Arumugam S. (2015). Endophilin-A2 functions in membrane scission in clathrin-independent endocytosis. *Nature* 517 493–496. 10.1038/nature14064 25517096 PMC4342003

[B240] Renner L. D. Weibel D. B. (2012). MinD and MinE interact with anionic phospholipids and regulate division plane formation in *Escherichia coli*. *J. Biol. Chem.* 287 38835–38844. 10.1074/jbc.m112.407817 23012351 PMC3493925

[B241] Roberts K. L. Leser G. P. Ma C. Lamb R. A. (2013). The amphipathic helix of influenza A virus M2 protein is required for filamentous bud formation and scission of filamentous and spherical particles. *J. Virol.* 87 9973–9982. 10.1128/jvi.01363-13 23843641 PMC3754012

[B242] Robotta M. Gerding H. R. Vogel A. Hauser K. Schildknecht S. Karreman C. (2014). α-synuclein binds to the inner membrane of mitochondria in an α-helical conformation. *Chembiochem* 15 2499–2502. 10.1002/cbic.201402281 25209675

[B243] Rossman J. S. Jing X. Leser G. P. Lamb R. A. (2010). Influenza virus M2 protein mediates ESCRT-independent membrane scission. *Cell* 142 902–913. 10.1016/j.cell.2010.08.029 20850012 PMC3059587

[B244] Rossman J. S. Lamb R. A. (2013). Viral membrane scission. *Annu. Rev. Cell Dev. Biol.* 29 551–569. 10.1146/annurev-cellbio-101011-155838 24099087 PMC4286373

[B245] Rostovtseva T. K. Boukari H. Antignani A. Shiu B. Banerjee S. Neutzner A. (2009). Bax activates endophilin B1 oligomerization and lipid membrane vesiculation. *J. Biol. Chem.* 284 34390–34399. 10.1074/jbc.m109.021873 19805544 PMC2797207

[B246] Ruprecht J. J. Hellawell A. M. Harding M. Crichton P. G. McCoy A. J. Kunji E. R. S. (2014). Structures of yeast mitochondrial ADP/ATP carriers support a domain-based alternating-access transport mechanism. *Proc. Natl. Acad. Sci. U.S.A.* 111 E426–434. 24474793 10.1073/pnas.1320692111 PMC3910652

[B247] Ryan T. Bamm V. V. Stykel M. G. Coackley C. L. Humphries K. M. Jamieson-Williams R. (2018). Cardiolipin exposure on the outer mitochondrial membrane modulates α-synuclein. *Nat. Commun.* 9:817. 10.1038/s41467-018-03241-9 PMC5827019 29483518

[B248] Saksena S. Wahlman J. Teis D. Johnson A. E. Emr S. D. (2009). Functional reconstitution of ESCRT-III assembly and disassembly. *Cell* 136 97–109. 10.1016/j.cell.2008.11.013 19135892 PMC4104304

[B249] Sandvig K. Kavaliauskiene S. Skotland T. (2018). Clathrin-independent endocytosis: an increasing degree of complexity. *Histochem. Cell Biol.* 150 107–118. 10.1007/s00418-018-1678-5 29774430 PMC6096564

[B250] Sani M.-A. Whitwell T. C. Separovic F. (2012). Lipid composition regulates the conformation and insertion of the antimicrobial peptide maculatin 1.1. *Biochim. Biophys. Acta* 1818 205–211. 10.1016/j.bbamem.2011.07.015 21801711

[B251] Sathappa M. Alder N. N. (2016). The ionization properties of cardiolipin and its variants in model bilayers. *Biochim. Biophys. Acta* 1858 1362–1372. 10.1016/j.bbamem.2016.03.007 26965987 PMC4897776

[B252] Scacioc A. Schmidt C. Hofmann T. Urlaub H. Kühnel K. Pérez-Lara A. (2017). Structure based biophysical characterization of the PROPPIN Atg18 shows Atg18 oligomerization upon membrane binding. *Sci. Rep.* 7:14008. 10.1038/s41598-017-14337-5 PMC5656675 29070817

[B253] Schöneberg J. Lee I.-H. Iwasa J. H. Hurley J. H. (2017). Reverse-topology membrane scission by the ESCRT proteins. *Nat. Rev. Mol. Cell Biol.* 18 5–17. 10.1038/nrm.2016.121 27703243 PMC5198518

[B254] Schrader M. Costello J. L. Godinho L. F. Azadi A. S. Islinger M. (2016). Proliferation and fission of peroxisomes - An update. *Biochim. Biophys. Acta* 1863 971–983. 10.1016/j.bbamcr.2015.09.024 26409486

[B255] Schwechheimer C. Kuehn M. J. (2015). Outer-membrane vesicles from Gram-negative bacteria: biogenesis and functions. *Nat. Rev. Microbiol.* 13 605–619. 10.1038/nrmicro3525 26373371 PMC5308417

[B256] Scott I. Youle R. J. (2010). Mitochondrial fission and fusion. *Essays Biochem.* 47 85–98. 20533902 10.1042/bse0470085 PMC4762097

[B257] Segrest J. P. De Loof H. Dohlman J. G. Brouillette C. G. Anantharamaiah G. M. (1990). Amphipathic helix motif: Classes and properties. *Proteins* 8 103–117. 10.1002/prot.340080202 2235991

[B258] Segrest J. P. Jones M. K. De Loof H. Brouillette C. G. Venkatachalapathi Y. V. Anantharamaiah G. M. (1992). The amphipathic helix in the exchangeable apolipoproteins: a review of secondary structure and function. *J. Lipid Res.* 33 141–166. 1569369

[B259] Sen A. Madhivanan K. Mukherjee D. Aguilar R. C. (2012). The epsin protein family: coordinators of endocytosis and signaling. *Biomol. Concepts* 3 117–126. 22942912 10.1515/bmc-2011-0060 PMC3431554

[B260] Simunovic M. Evergren E. Callan-Jones A. Bassereau P. (2019). Curving cells inside and out: roles of BAR domain proteins in membrane shaping and its cellular implications. *Annu. Rev. Cell Dev. Biol.* 35 111–129 10.1146/annurev-cellbio-100617-060558 31340125

[B261] Simunovic M. Manneville J.-B. Renard H.-F. Evergren E. Raghunathan K. Bhatia D. (2017). Friction mediates scission of tubular membranes scaffolded by BAR proteins. *Cell* 170 172.e11–184.e11. 28648660 10.1016/j.cell.2017.05.047 PMC5576516

[B262] Snead W. T. Hayden C. C. Gadok A. K. Zhao C. Lafer E. M. Rangamani P. (2017). Membrane fission by protein crowding. *Proc. Natl. Acad. Sci. U.S.A.* 114 E3258–E3267. 28373566 10.1073/pnas.1616199114 PMC5402459

[B263] Snead W. T. Stachowiak J. C. (2018). Structure *versus* stochasticity-the role of molecular crowding and intrinsic disorder in membrane fission. *J. Mol. Biol.* 430 2293–2308. 10.1016/j.jmb.2018.03.024 29627460 PMC6045432

[B264] Snead W. T. Zeno W. F. Kago G. Perkins R. W. Richter J. B. Zhao C. (2019). BAR scaffolds drive membrane fission by crowding disordered domains. *J. Cell Biol.* 218 664–682. 10.1083/jcb.201807119 30504247 PMC6363457

[B265] Soni S. P. Stahelin R. V. (2014). The Ebola virus matrix protein VP40 selectively induces vesiculation from phosphatidylserine-enriched membranes. *J. Biol. Chem.* 289 33590–33597. 10.1074/jbc.m114.586396 25315776 PMC4246110

[B266] Spanò S. Silletta M. G. Colanzi A. Alberti S. Fiucci G. Valente C. (1999). Molecular cloning and functional characterization of brefeldin A-ADP-ribosylated substrate. A novel protein involved in the maintenance of the Golgi structure. *J. Biol. Chem.* 274 17705–17710. 10.1074/jbc.274.25.17705 10364211

[B267] Stanishneva-Konovalova T. B. Derkacheva N. I. Polevova S. V. Sokolova O. S. (2016). The role of BAR domain proteins in the regulation of membrane dynamics. *Acta Nat.* 8 60–69. PMC5199207 28050267

[B268] Stoten C. L. Carlton J. G. (2018). ESCRT-dependent control of membrane remodelling during cell division. *Semin. Cell Dev. Biol.* 74 50–65. 10.1016/j.semcdb.2017.08.035 28843980 PMC6015221

[B269] Su J. Thomas A. S. Grabietz T. Landgraf C. Volkmer R. Marrink S. J. (2018). The N-terminal amphipathic helix of Pex11p self-interacts to induce membrane remodelling during peroxisome fission. *Biochim. Biophys. Acta Biomembr.* 1860 1292–1300. 10.1016/j.bbamem.2018.02.029 29501607

[B270] Suetsugu S. Kurisu S. Takenawa T. (2014). Dynamic shaping of cellular membranes by phospholipids and membrane-deforming proteins. *Physiol. Rev.* 94 1219–1248. 10.1152/physrev.00040.2013 25287863

[B271] Sundborger A. Soderblom C. Vorontsova O. Evergren E. Hinshaw J. E. Shupliakov O. (2011). An endophilin-dynamin complex promotes budding of clathrin-coated vesicles during synaptic vesicle recycling. *J. Cell Sci.* 124 133–143. 10.1242/jcs.072686 21172823 PMC3001412

[B272] Suresh S. Edwardson J. M. (2010). The endophilin N-BAR domain perturbs the structure of lipid bilayers. *Biochemistry* 49 5766–5771. 10.1021/bi100760e 20527805

[B273] Takahashi Y. Meyerkord C. L. Hori T. Runkle K. Fox T. E. Kester M. (2011). Bif-1 regulates Atg9 trafficking by mediating the fission of Golgi membranes during autophagy. *Autophagy* 7 61–73. 10.4161/auto.7.1.14015 21068542 PMC3039731

[B274] Takahashi Y. Tsotakos N. Liu Y. Young M. M. Serfass J. Tang Z. (2016). The Bif-1-Dynamin 2 membrane fission machinery regulates Atg9-containing vesicle generation at the Rab11-positive reservoirs. *Oncotarget* 7 20855–20868. 26980706 10.18632/oncotarget.8028 PMC4991497

[B275] Takatori S. Tatematsu T. Cheng J. Matsumoto J. Akano T. Fujimoto T. (2016). Phosphatidylinositol 3,5-Bisphosphate-rich membrane domains in endosomes and lysosomes. *Traffic* 17 154–167. 10.1111/tra.12346 26563567

[B276] Takatori S. Tomita T. (2018). AP180 N-Terminal Homology (ANTH) and Epsin N-Terminal Homology (ENTH) domains: physiological functions and involvement in disease. *Adv. Exp. Med. Biol.* 10.1007/5584_2018_218 [Epub ahead of print] 29774507

[B277] Takeda T. Kozai T. Yang H. Ishikuro D. Seyama K. Kumagai Y. (2018). Dynamic clustering of dynamin-amphiphysin helices regulates membrane constriction and fission coupled with GTP hydrolysis. *eLife* 7:e30246. 10.7554/eLife.30246 PMC5780043 29357276

[B278] Tamura N. Nishimura T. Sakamaki Y. Koyama-Honda I. Yamamoto H. Mizushima N. (2017). Differential requirement for ATG2A domains for localization to autophagic membranes and lipid droplets. *FEBS Lett.* 591 3819–3830. 10.1002/1873-3468.12901 29113029

[B279] Tandler B. Hoppel C. L. Mears J. A. (2018). Morphological Pathways of mitochondrial division. *Antioxidants* 7:E30. 10.3390/antiox7020030 PMC5836020 29462856

[B280] Tang Z. Takahashi Y. Chen C. Liu Y. He H. Tsotakos N. (2017). Atg2A/B deficiency switches cytoprotective autophagy to non-canonical caspase-8 activation and apoptosis. *Cell Death Differ.* 24 2127–2138. 10.1038/cdd.2017.133 28800131 PMC5686350

[B281] Tanguy E. Wang Q. Moine H. Vitale N. (2019). Phosphatidic Acid: from pleiotropic functions to neuronal pathology. *Front. Cell Neurosci.* 13:2. 10.3389/fncel.2019.00002 30728767 PMC6351798

[B282] Taschenberger G. Toloe J. Tereshchenko J. Akerboom J. Wales P. Benz R. (2013). β-synuclein aggregates and induces neurodegeneration in dopaminergic neurons. *Ann. Neurol.* 74 109–118. 10.1002/ana.23905 23536356

[B283] Tilokani L. Nagashima S. Paupe V. Prudent J. (2018). Mitochondrial dynamics: overview of molecular mechanisms. *Essays Biochem.* 62 341–360. 10.1042/ebc20170104 30030364 PMC6056715

[B284] Uebe R. Schüler D. (2016). Magnetosome biogenesis in magnetotactic bacteria. *Nat. Rev. Microbiol.* 14 621–637. 10.1038/nrmicro.2016.99 27620945

[B285] Uttenweiler A. Mayer A. (2008). Microautophagy in the yeast Saccharomyces cerevisiae. *Methods Mol Biol* 445 245–259. 10.1007/978-1-59745-157-4_16 18425455

[B286] Valente C. Luini A. Corda D. (2013). Components of the CtBP1/BARS-dependent fission machinery. *Histochem. Cell Biol.* 140 407–421. 10.1007/s00418-013-1138-1 23996193

[B287] Valente C. Spanò S. Luini A. Corda D. (2005). Purification and functional properties of the membrane fissioning protein CtBP3/BARS. *Methods Enzymol.* 404 296–316. 10.1016/s0076-6879(05)04027-9 16413278

[B288] Valente C. Turacchio G. Mariggiò S. Pagliuso A. Gaibisso R. Di Tullio G. (2012). A 14-3-3γ dimer-based scaffold bridges CtBP1-S/BARS to PI(4)KIIIβ to regulate post-Golgi carrier formation. *Nat. Cell Biol.* 14 343–354. 10.1038/ncb2445 22366688

[B289] Vamparys L. Gautier R. Vanni S. Bennett W. F. Tieleman D. P. Antonny B. (2013). Conical lipids in flat bilayers induce packing defects similar to that induced by positive curvature. *Biophys. J.* 104 585–593. 10.1016/j.bpj.2012.11.3836 23442909 PMC3566444

[B290] van den Brink-van der Laan E. Boots J.-W. P. Spelbrink R. E. J. Kool G. M. Breukink E. Killian J. A. (2003). Membrane interaction of the glycosyltransferase MurG: a special role for cardiolipin. *J. Bacteriol.* 185 3773–3779. 10.1128/jb.185.13.3773-3779.2003 12813070 PMC161595

[B291] van Paridon P. A. de Kruijff B. Ouwerkerk R. Wirtz K. W. A. (1986). Polyphosphoinositides undergo charge neutralization in the physiological pH range: a ^31^P-NMR study. *Biochim. Biophys. Acta* 877 216–219. 10.1016/0005-2760(86)90137-2 3013316

[B292] Vanni S. Vamparys L. Gautier R. Drin G. Etchebest C. Fuchs P. F. J. (2013). Amphipathic lipid packing sensor motifs: probing bilayer defects with hydrophobic residues. *Biophys. J.* 104 575–584. 10.1016/j.bpj.2012.11.3837 23442908 PMC3566459

[B293] Varkey J. Isas J. M. Mizuno N. Jensen M. B. Bhatia V. K. Jao C. C. (2010). Membrane curvature induction and tubulation are common features of synucleins and apolipoproteins. *J. Biol. Chem.* 285 32486–32493. 10.1074/jbc.m110.139576 20693280 PMC2952250

[B294] Vedyaykin A. D. Ponomareva E. V. Khodorkovskii M. A. Borchsenius S. N. Vishnjakov I. E. (2019). Mechanisms of bacterial cell division. *Microbiology* 88 245–260.

[B295] Velikkakath A. K. G. Nishimura T. Oita E. Ishihara N. Mizushima N. (2012). Mammalian Atg2 proteins are essential for autophagosome formation and important for regulation of size and distribution of lipid droplets. *Mol. Biol. Cell* 23 896–909. 10.1091/mbc.e11-09-0785 22219374 PMC3290647

[B296] Votteler J. Sundquist W. I. (2013). Virus budding and the ESCRT pathway. *Cell Host Microbe* 14 232–241. 10.1016/j.chom.2013.08.012 24034610 PMC3819203

[B297] Weigert R. Silletta M. G. Spanò S. Turacchio G. Cericola C. Colanzi A. (1999). CtBP/BARS induces fission of Golgi membranes by acylating lysophosphatidic acid. *Nature* 402 429–433. 10.1038/46587 10586885

[B298] Wilz L. Fan W. Zhong Q. (2011). Membrane curvature response in autophagy. *Autophagy* 7 1249–1250. 10.4161/auto.7.10.16738 21738011 PMC3210310

[B299] Wriessnegger T. Gübitz G. Leitner E. Ingolic E. Cregg J. de la Cruz B. J. (2007). Lipid composition of peroxisomes from the yeast Pichia pastoris grown on different carbon sources. *Biochim. Biophys. Acta* 1771 455–461. 10.1016/j.bbalip.2007.01.004 17293161

[B300] Wu T. Baumgart T. (2014). BIN1 membrane curvature sensing and generation show autoinhibition regulated by downstream ligands and PI(4,5)P_2_. *Biochemistry* 53 7297–7309. 10.1021/bi501082r 25350771 PMC4245986

[B301] Wu Y. Matsui H. Tomizawa K. (2009). Amphiphysin I and regulation of synaptic vesicle endocytosis. *Acta Med. Okayama* 63 305–323. 20035287 10.18926/AMO/31822

[B302] Yang J.-S. Gad H. Lee S. Y. Mironov A. Zhang L. Beznoussenko G. V. (2008). A role for phosphatidic acid in COPI vesicle fission yields insights into Golgi maintenance. *Nat. Cell Biol.* 10 1146–1153. 10.1038/ncb1774 18776900 PMC2756218

[B303] Yang J.-S. Lee S. Y. Spanò S. Gad H. Zhang L. Nie Z. (2005). A role for BARS at the fission step of COPI vesicle formation from Golgi membrane. *EMBO J.* 24 4133–4143. 10.1038/sj.emboj.7600873 16292346 PMC1356313

[B304] Yang J.-S. Valente C. Polishchuk R. S. Turacchio G. Layre E. Moody D. B. (2011). COPI acts in both vesicular and tubular transport. *Nat. Cell Biol.* 13 996–1003. 10.1038/ncb2273 21725317 PMC3149785

[B305] Yoon Y. Tong J. Lee P. J. Albanese A. Bhardwaj N. Källberg M. (2010). Molecular basis of the potent membrane-remodeling activity of the epsin 1 N-terminal homology domain. *J. Biol. Chem.* 285 531–540. 10.1074/jbc.m109.068015 19880963 PMC2804201

[B306] Yoon Y. Zhang X. Cho W. (2012). Phosphatidylinositol 4,5-bisphosphate (PtdIns(4,5)P_2_) specifically induces membrane penetration and deformation by Bin/Amphiphysin/Rvs (BAR) domains. *J. Biol. Chem.* 287 34078–34090. 10.1074/jbc.m112.372789 22888025 PMC3464517

[B307] Yorimitsu T. Sato K. Takeuchi M. (2014). Molecular mechanisms of Sar/Arf GTPases in vesicular trafficking in yeast and plants. *Front. Plant Sci.* 5:411. 10.3389/fpls.2014.00411 25191334 PMC4140167

[B308] Yoshida Y. (2018). Insights into the mechanisms of chloroplast division. *Int. J. Mol. Sci.* 19:E733. 10.3390/ijms19030733 PMC5877594 29510533

[B309] Yoshida Y. Mogi Y. (2019). How do plastids and mitochondria divide? *Microscopy* 68 45–56. 10.1093/jmicro/dfy132 30476140

[B310] Yoshida Y. Niwa H. Honsho M. Itoyama A. Fujiki Y. (2015). Pex11 mediates peroxisomal proliferation by promoting deformation of the lipid membrane. *Biol. Open* 4 710–721. 10.1242/bio.201410801 25910939 PMC4467191

[B311] Yu S. Melia T. J. (2017). The coordination of membrane fission and fusion at the end of autophagosome maturation. *Curr. Opin. Cell Biol.* 47 92–98. 10.1016/j.ceb.2017.03.010 28463755 PMC5537011

[B312] Zarranz J. J. Alegre J. Gómez-Esteban J. C. Lezcano E. Ros R. Ampuero I. (2004). The new mutation, E46K, of α-synuclein causes Parkinson and Lewy body dementia. *Ann. Neurol.* 55 164–173. 10.1002/ana.10795 14755719

[B313] Zegarlińska J. Piaścik M. Sikorski A. F. Czogalla A. (2018). Phosphatidic acid - a simple phospholipid with multiple faces. *Acta Biochim. Pol.* 65 163–171. 10.18388/abp.2018_2592 29913482

[B314] Zemel A. Ben-Shaul A. May S. (2008). Modulation of the spontaneous curvature and bending rigidity of lipid membranes by interfacially adsorbed amphipathic peptides. *J. Phys. Chem. B* 112 6988–6996. 10.1021/jp711107y 18479112

[B315] Zhang C. Li A. Gao S. Zhang X. Xiao H. (2011). The TIP30 protein complex, arachidonic acid and coenzyme A are required for vesicle membrane fusion. *PLoS One* 6:e21233. 10.1371/journal.pone.0021233 21731680 PMC3123320

[B316] Zhou H. Lutkenhaus J. (2003). Membrane binding by MinD involves insertion of hydrophobic residues within the C-terminal amphipathic helix into the bilayer. *J. Bacteriol.* 185 4326–4335. 10.1128/jb.185.15.4326-4335.2003 12867440 PMC165746

[B317] Zhukovsky M. A. Filograna A. Luini A. Corda D. Valente C. (2019a). Phosphatidic acid in membrane rearrangements. *FEBS Lett*. 593 2428–2451 10.1002/1873-3468.13563 31365767

[B318] Zhukovsky M. A. Filograna A. Luini A. Corda D. Valente C. (2019b). The structure and function of acylglycerophosphate acyltransferase 4/Lysophosphatidic acid acyltransferase delta (AGPAT4/LPAATδ). *Front. Cell Dev. Biol.* 7:147. 10.3389/fcell.2019.00147 31428612 PMC6688108

